# Targeting SARS-CoV-2 Main Protease for Treatment of COVID-19: Covalent Inhibitors Structure–Activity Relationship Insights and Evolution Perspectives

**DOI:** 10.1021/acs.jmedchem.2c01005

**Published:** 2022-09-28

**Authors:** Gabriele La Monica, Alessia Bono, Antonino Lauria, Annamaria Martorana

**Affiliations:** Dipartimento di Scienze e Tecnologie Biologiche Chimiche e Farmaceutiche, University of Palermo, Viale delle Scienze, Ed. 17, I-90128 Palermo, Italy

## Abstract

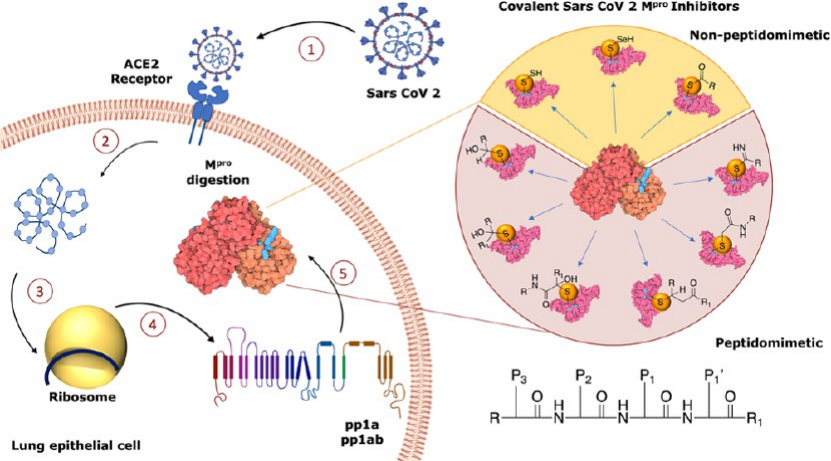

The viral main protease is one of the most attractive targets among all key enzymes involved in the SARS-CoV-2 life cycle. Covalent inhibition of the cysteine^145^ of SARS-CoV-2 M^PRO^ with selective antiviral drugs will arrest the replication process of the virus without affecting human catalytic pathways. In this Perspective, we analyzed the in silico, in vitro, and in vivo data of the most representative examples of covalent SARS-CoV-2 M^PRO^ inhibitors reported in the literature to date. In particular, the studied molecules were classified into eight different categories according to their reactive electrophilic warheads, highlighting the differences between their reversible/irreversible mechanism of inhibition. Furthermore, the analyses of the most recurrent pharmacophoric moieties and stereochemistry of chiral carbons were reported. The analyses of noncovalent and covalent in silico protocols, provided in this Perspective, would be useful for the scientific community to discover new and more efficient covalent SARS-CoV-2 M^PRO^ inhibitors.

## 1 Introduction

The ongoing coronavirus disease-19 (COVID-19) global pandemic is etiologically caused by the highly infectious severe acute respiratory syndrome coronavirus 2 (SARS-CoV-2).^1,2^ SARS-CoV-2 is a β-coronavirus able to infect animals and humans, causing respiratory diseases.^3^

In this outbreak scenario, in order to contain the infection, the scientific community suggests strong social restraint measures and active development of vaccines. More than 20 vaccines reached clinical trials, demonstrating their efficacy in the prevention of severe COVID-19 syndrome.^4^ Considering the rapid mutation rate of this virus family, the long-term efficacy and safety of them is currently debated; for this reason, a more specific pharmacological treatment is needed to complement vaccines both in the prophylaxis and in the treatment of COVID-19.^5^ Moreover, there is an urgent need to develop effective therapeutic strategies against SARS-CoV-2; in this light, drug repurposing has been an effective approach over the last 2 years, based in particular on the comparative overview between CoVs. nafamostat/camostat,^6^ umifenovir,^7^ chloroquine/hydroxychloroquine,^8^ lopinavir/ritonavir,^9^ and remdesivir,^10^ which are just a few examples of drugs approved for other therapeutic applications that could be used to treat COVID-19.

To date, with the goal of developing a selective antiviral drug against SARS-CoV-2, the research community worldwide evaluated several structural/nonstructural viral proteins as possible SARS-CoV-2 druggable targets. Among these, the main protease (M^PRO^) emerged as the most attractive target for drug design.^11^

### 1.1 SARS-CoV-2 M^PRO^: A Covalently Druggable Target

**Figure 1:**
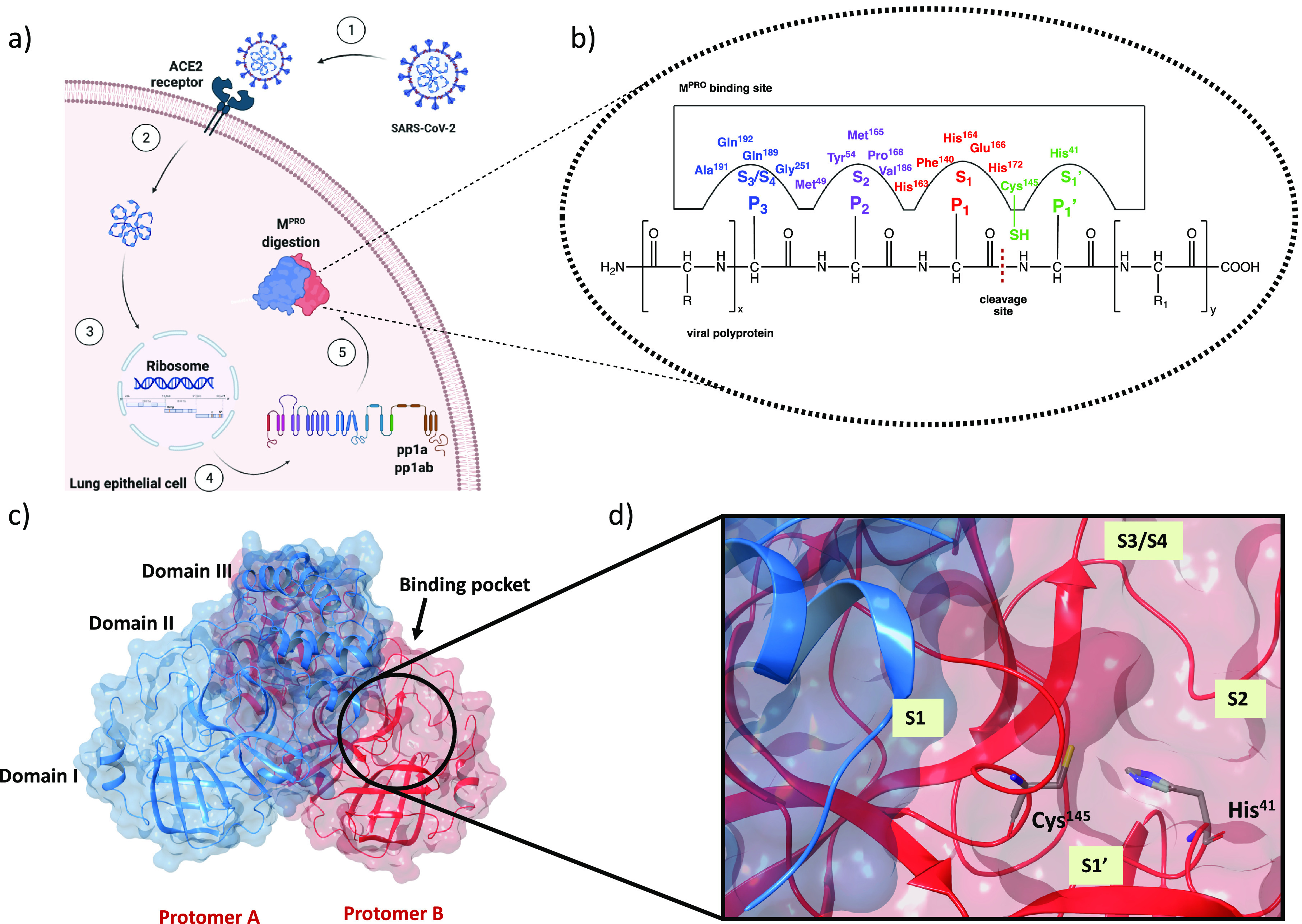
(a) Sequence of events necessary to activate the digestion process of M^PRO^: (1) virus entry into lung epithelial cell by interaction with ACE2 receptor, (2) release of the genomic material of SARS-CoV-2, (3 and 4) virus protein synthesis in human ribosomes, and (5) M^PRO^ digestion. (b) SARS-CoV-2 M^PRO^ binding site with viral polyproteins. (c) X-ray structure of dimeric SARS-CoV-2 M^PRO^ (PDB code 6Y2F). (d) Focus on the catalytic site, with the four regions S1, S1′, S2, and S3/S4 highlighted.^14^

The main protease M^PRO^, also known as 3C-like protease (3CL^PRO^), is a key cysteinyl enzyme involved in the digestion of the polyproteins pp1a and pp1ab. It acts at 11 conserved sites with the subsequent release of 12 nonstructural proteins (Nsp, essential for the replication/transcription process), Figure 1a.

In the viral polyproteins, the amino acids from the N-terminus to the C-terminus are indicated as −P3–P2–P1↓P1′–, where P1 is generally a Gln and the arrow ↓ corresponds to the amide bond cleavage site between P1 and a small P1′ amino acid, such as serine, glycine, or alanine residues. M^PRO^ recognizes the glutamine amino acid residues and cleaves the peptide bond between Gln and the adjacent amino acid, Figure 1b. The inhibition of its enzymatic activity could drive blockage of viral replication.^12^

Structurally, M^PRO^ is a homodimer, and each protomer consists of 306 amino acids divided into 3 domains (I, II, and III): domain I (8–101) comprises 6 β-strands and an α-helix, while domain II (102–184) and domain III (201–303) include 6 β-strands and 5 α-helices, respectively. All domains are connected by long loop regions (Figure 1c).^13^ The binding pocket is organized into four subsites, S1′, S1, S2, and S3/S4, which are occupied by the portions P1′, P1, P2, and P3 of the viral polyproteins, respectively (Figure 1d).

In region S1′, between domains I and II, the catalytic site is located, characterized by the catalytic dyad (Cys^145^ and His^41^). During hydrolysis of the peptide bond, His^41^ activates the nucleophilic −SH of Cys^145^ by deprotonation with subsequent stabilization of the adduct by the so-called “oxyanion hole”, formed by the Gly^143^ and Cys^145^ backbones. The S1 region, characterized by the side chains of Phe^140^, His^163^, His^164^, Glu^166^, and His^172^, is highly specific for the glutamine residue.^15^ Region S2 consists of hydrophobic amino acids, such as Met^49^, Tyr^54^, Met^165^, Pro^168^, Val^186^, and finally, regions S3/S4, which are particularly exposed to the solvent, involve Gln^189^, Ala^191^, Gln^192^, and Gly^251^ residues. The peculiar structure of the binding pocket suggests SARS-CoV-2 M^PRO^ as a druggable target for small molecules.^5,12^ In this scenario, many efforts have been devoted to identifying new selective peptidomimetic inhibitors of SARS-CoV-2 M^PRO^.^5^

**Figure 2:**
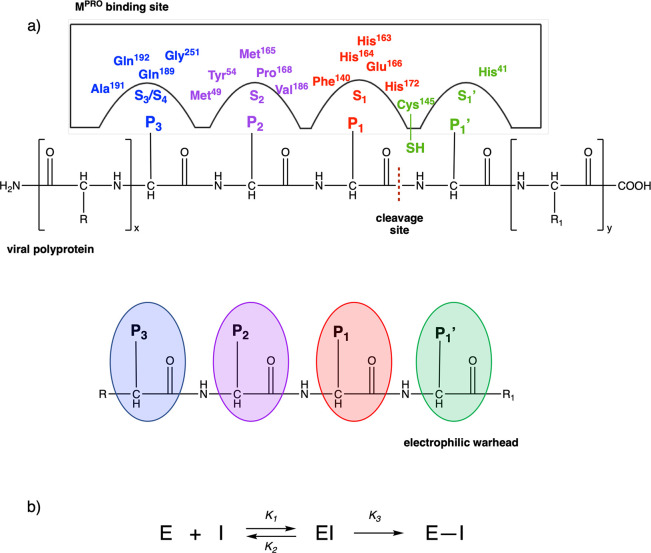
(a) Covalent inhibition of SARS-CoV-2 M^PRO^. P1′, P1, P2, and P3 labels reflect the chemical analogies with the viral substrate. Warheads P1′ are in green, while fragments P1, P2, and P3 are shown in red, purple, and blue, respectively. Subregions of the binding pocket are labeled with S numbering complementary to the fragments of the inhibitor. (b) Kinetic scheme of covalent inhibition. E, I, EI, and E–I stand for enzyme, inhibitor, noncovalent enzyme–inhibitor complex, and covalent enzyme–inhibitor complex, respectively.

An interesting approach has been provided by the covalent inhibition of the SARS-CoV-2 M^PRO^ cysteinyl protease:^16−18^ the catalytic sulfur of Cys^145^ is covalently trapped by an electrophilic moiety P1′, a reactive warhead that mimics the amide peptide of the viral polyproteins. To be successful in the covalent inhibition, the inhibitors must meet basic structural requirements: a P1 portion, usually a cyclic glutamine analogue, capable of interacting with amino acids such as His^163^, Glu^166^, and His^172^ via hydrogen bonds and hydrophobic interactions; P2 and P3 moieties, projected into hydrophobic pockets (S2 and S3/S4) and characterized by substituent groups capable of favorable hydrophobic interactions with the amino acids of these sites (Figure 2a).

In this perspective, the kinetic scheme of covalent inhibition deserves special attention. As shown in Figure 2b, the process of enzymatic inhibition involves two steps: (1) the inhibitor (I) reversibly binds to the active site of the enzyme (E) and forms a noncovalent enzyme–inhibitor complex (EI); (2) due to the nucleophilic attack of the catalytic Cys^145^, the noncovalent complex evolves into a more stable complex (E–I), which generally irreversibly inactivates the catalytic activity. From a kinetic point of view, the first phase is correlated with the equilibrium-binding constant *K*_i_ (*K*_i_ = *k*_2_/*k*_1_), while the second stage is influenced by the inactivation constant for covalent bond formation *k*_3_ (*K*_inact_).^19^

The covalent inhibition strategy has already been pursued against other proteases, including MERS and SARS-CoV-1, with remarkable results.^20^ Indeed, compared to traditional noncovalent agents, the electrophilic warhead inhibitors provide better efficacy, higher potency, longer residence time in the binding site of the receptor, sustained pharmacological action, and the ability to overcome resistance.^16−18^

In the last 2 years, the whole scientific community has invested a lot of time and resources to design new covalent SARS-CoV-2 M^PRO^ inhibitors, and in this context, some small molecules have already reached the most advanced phases of drug development. PF-07321332, a nitrile compound developed by Pfizer as a covalent SARS-CoV-2 M^PRO^ inhibitor, entered clinical trials in combination with ritonavir showing promising results in phase 2/3 (PAXLOVID) and was recently approved by the Committee for Medicinal Products for Human use of the EMA for the treatment of COVID-19.^21−23^

Considering the increasing interest of medicinal chemistry researchers in the design of new more powerful and selective covalent SARS-CoV-2 M^PRO^ inhibitors, the main purpose of this Perspective is to provide an overview of the most representative examples reported to date in the literature in this field. In detail, the collected inhibitors were classified into different categories according to their reactive electrophilic group, underling the differences between their mechanism of inhibition.

Furthermore, with the support of the numerous available X-ray structures of these compounds in complex with the SARS-CoV-2 M^PRO^ protease, an analysis of the most recurrent pharmacophoric portions is reported to evaluate the most suitable fragments for interaction with the different subsites of the catalytic cleft of the main protease. Biological data, such as inhibition of M^PRO^ activity, antiviral effect in vitro, and in vivo results, are examined. This in-depth analysis aims to support the design of promising small molecules and the discovery of new, more selective SARS-CoV-2 M^PRO^ covalent inhibitors.

## 2 Electrophilic Warheads in Covalent SARS-CoV-2 M^PRO^ Inhibition

**Figure 3:**
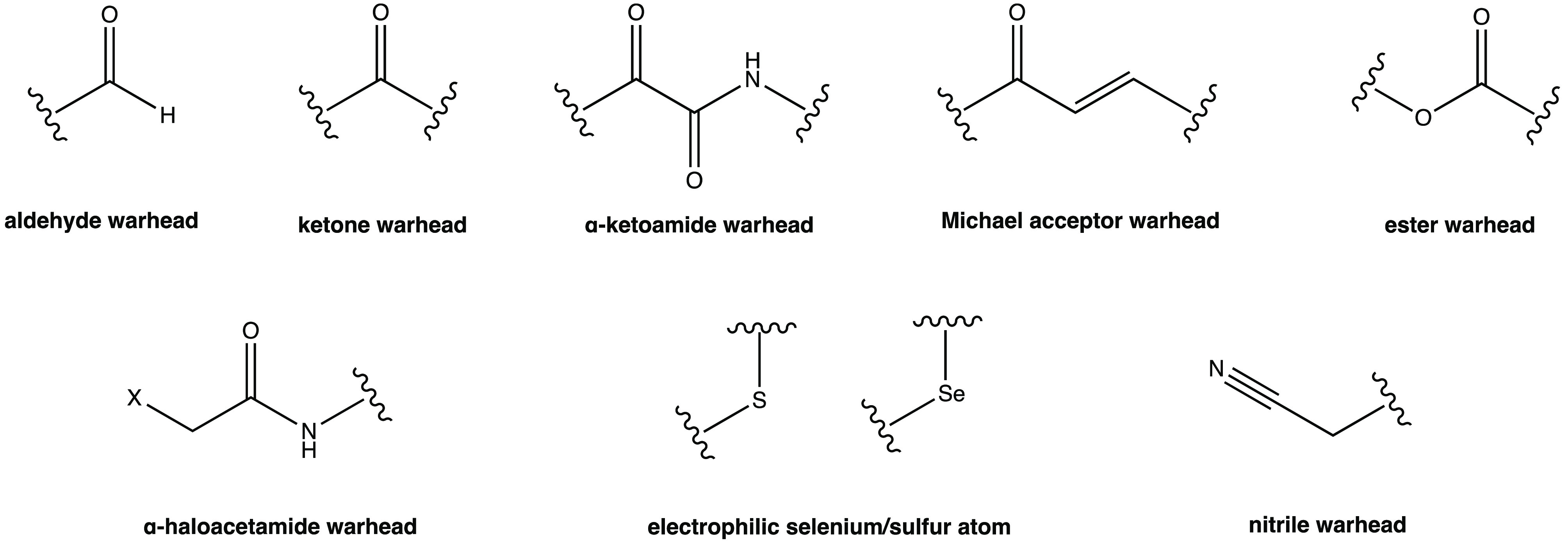
Electrophilic warheads characterizing covalent SARS-CoV-2 M^PRO^ inhibitors.

In this work, a detailed analysis of the most interesting covalent SARS-CoV-2 M^PRO^ inhibitors is reported. According to the nature of the electrophilic warheads, the small molecules were divided into eight groups: aldehydes, ketones, α-ketoamides, Michael acceptors, α-haloacetamides, nitriles, esters, and molecules containing an electrophilic selenium/sulfur atom (Figure 3). Most of the analyzed compounds exhibited a dipeptidomimetic or tripeptidomimetic structure (such as carbonyl, α-ketoamide, Michael acceptor, α-haloacetamide, and nitrile derivatives), although some examples of nonpeptidomimetic SARS-CoV-2 M^PRO^ inhibitors were also reported (such as activated ester derivatives, natural compounds, or ebsulfur/ebselen derivatives). The differences between their mechanisms of action and the capability to form reversible/irreversible adducts were examined. To support the discussion and analysis of the binding mechanism, the X-ray structures of the most representative ligand–protein complexes were described in detail.

### 2.1 Carbonyl Warhead

**Figure 4:**
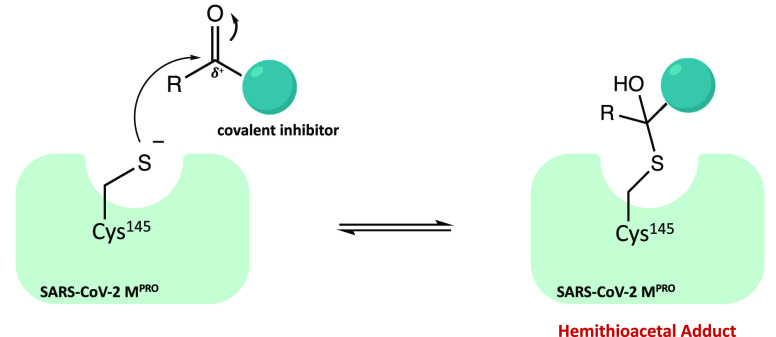
General mechanism of action of covalent carbonyl inhibitors.

Carbonyl groups, such as aldehydes and ketones, are considered the most promising warheads in the design of new covalent inhibitors of SARS-CoV-2 M^pro^. From a mechanistic point of view, their capability to form a covalent bond depends on the electrophilicity of the carbonyl carbon, which is susceptible to the nucleophilic addition of the cysteine-SH, leading to the formation of a reversible hemithioacetal adduct. The high similarity between the latter and the intermediate formed by the natural substrate during the enzymatic catalytic cycle ensures high stability of the inhibitor–protein complex and a longer residence time.^24^Figure 4 shows the general mechanism of covalent inhibition for this class of compounds.

In the panorama of covalent inhibitors with selective activity against SARS-CoV-2 M^PRO^, there are numerous examples of the carbonyl warhead, which is the most abundant in this class of small molecules.

#### 2.1.1 Aldehyde Warhead

**Figure 5:**
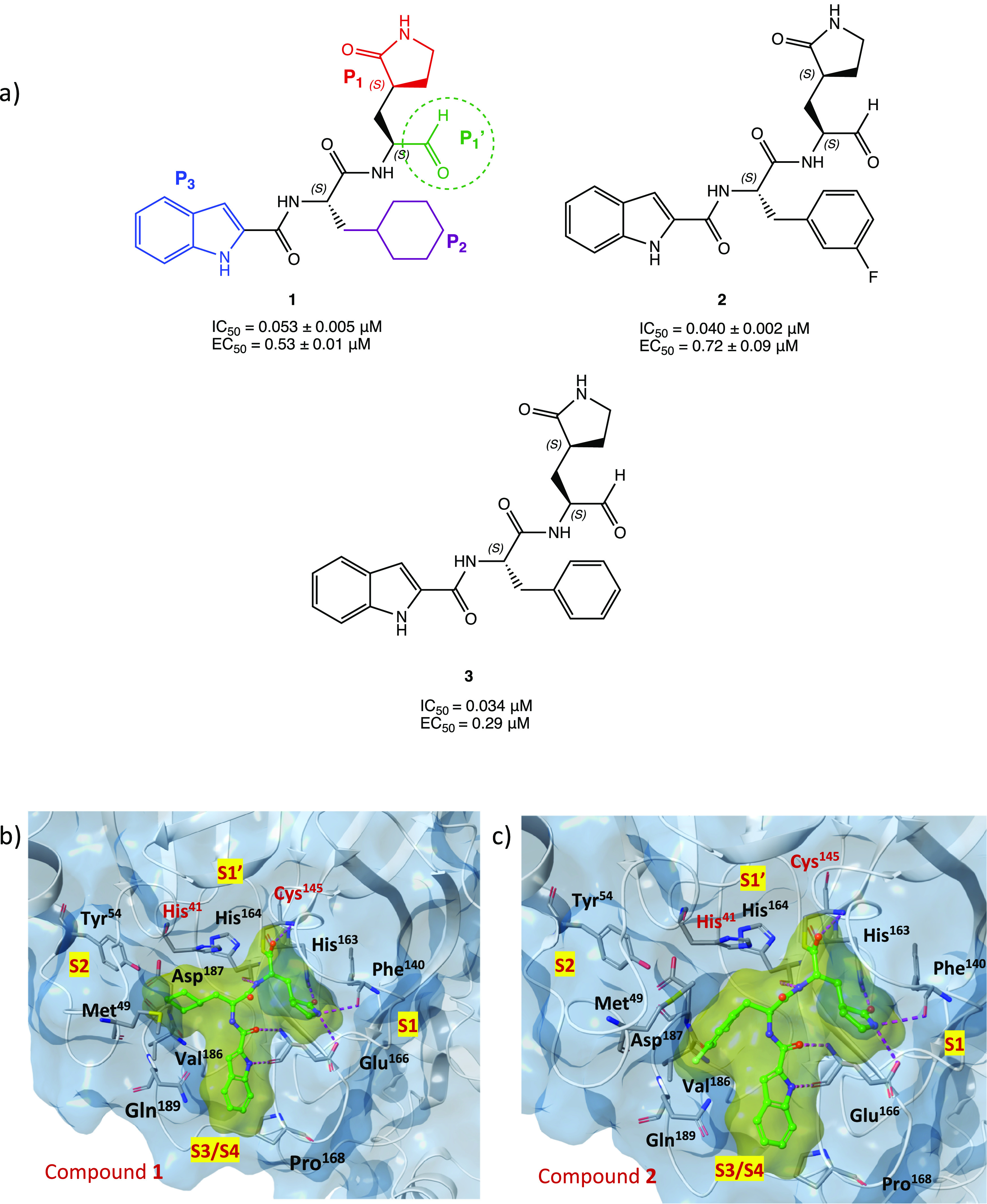
(a) Chemical structures of peptidomimetic aldehyde derivatives **1**–**3**; for derivative **1**, as an example for the whole series, the four key moieties P1′, P1, P2, and P3 for the interaction with SARS-CoV-2 M^PRO^ are highlighted. (b) X-ray structure of SARS-CoV-2 M^PRO^ in complex with compound **1** (PDB code 6LZE). (c) X-ray structure of SARS-CoV-2 M^PRO^ in complex with compound **2** (PDB code 6M0K). In both crystal structures, the amino acids involved in the interaction with the ligand and the four cavities of the binding pocket (S1′, S1, S2, and S3/S4) are shown.^25−27^

Among the covalent SARS-CoV-2 M^PRO^ inhibitors with an aldehyde warhead, compounds **1** and **2** were rationally designed as two of the first peptidomimetic derivatives (Figure 5a). In vitro inhibition assays proved excellent inhibitory activity against SARS-CoV-2 M^PRO^ (IC_50_ of 0.053 ± 0.005 and 0.040 ± 0.002 μM, respectively), strong anti-SARS-CoV-2 infectivity (EC_50_ of 0.53 ± 0.01 and 0.72 ± 0.09 μM, respectively), low cytotoxicity, and favorable pharmacokinetic and toxicity properties in vivo. Interestingly, the X-ray structures of both compounds in complex with M^PRO^, resolved at 1.5 Å, allowed a detailed analysis of the binding mode and mechanism of action of the proposed leads (Figure 5b and 5c shows SARS-CoV-2 M^PRO^ in complex with **1** and **2**, PDB codes 6LZE and 6M0K, respectively). To graphically illustrate the importance of each ligand moiety in stabilizing the complex, the four binding site cavities and the corresponding amino acids involved in the key interactions were highlighted. From the analysis, a similar binding mode of the two ligands was found: in both complexes, formation of the covalent bond between the aldehyde group (P1′) and the −SH of Cys^145^ at S1′ is enhanced by the additional H bond between the hemithioacetal group −OH and the cysteinyl backbone; the (*S*)-γ-lactam group (P1) is deeply inserted into the S1 site and, mimicking the glutamine residue of the natural substrates, can form three H bonds with three key amino acids of this cleft (one between His^163^ and the lactamic oxygen, two between Phe^140^ and Glu^166^ and the lactamic NH); the indole group (P3) is located at the surface (S3/S4 pocket) of the protein and interacts with Glu^166^. The only observed difference in the binding mode is represented by the P2 fragments, which differ between the two compounds (cyclohexyl and 3-fluorophenyl groups, respectively). Indeed, the 3-fluorophenyl moiety is more downrotated than the P2 in compound **1**. However, despite this difference in orientation, both fragments are deeply inserted into the S2 cavity and form extensive hydrophobic interactions.^25^

Furthermore, in light of the interesting results, derivative **1** was chosen as a representative compound to gain insight into the mechanism of action of aldehyde inhibitors at the atomistic level using QM/MM simulations: this type of molecular simulation permitted us to elucidate the mechanism of covalent bond formation and to highlight the proton transfer processes that take place within the catalytic dyad prior to the nucleophilic attack.^26^

In the search of new inhibitors of rhinovirus and enterovirus proteases, the peptidomimetic derivative **3** was synthesized (Figure 5), as an analogue of **2**, without the 3-F substitution on the phenyl ring. Tested against SARS-CoV-2 M^PRO^, compound **3** exhibited interesting inhibitory activity with an IC_50_ value of 0.034 μM and antiviral activity with EC_50_ = 0.29 μM. Structure–activity relationship (SAR) studies confirmed the importance of the γ-lactamic pentacyclic system and the indole ring: substitution with other heterocycles, such as quinoline, showed a drastic decrease in activity.^27^

**Figure 6:**
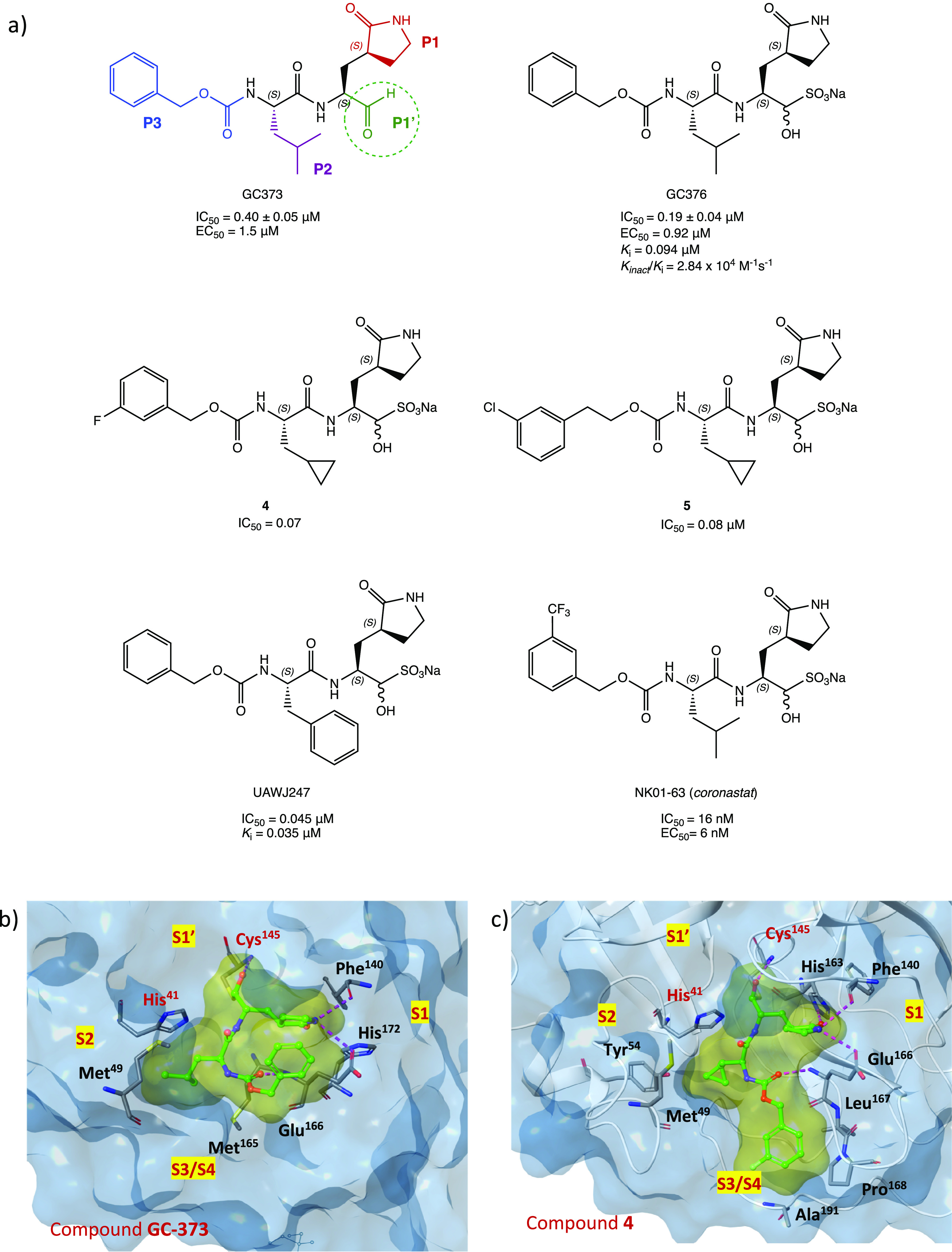
(a) Chemical structures of GC-373 (electrophilic P1′ warheads in green, P1 in red, P2 in purple, and P3 in blue), GC-376, analogues **4** and **5**, UAWJ247, and NK01-63 (coronastat).^28−30^ (b) X-ray structure of SARS-CoV-2 M^PRO^ in complex with GC-373 (PDB code 6WTJ(31)). (c) X-ray structure of SARS-CoV-2 M^PRO^ in complex with **4** (PDB code 7LCO(29)).

Other examples of promising SARS-CoV-2 M^PRO^ covalent inhibitors with an aldehyde warhead include GC-373 and the related prodrug GC-376 in which the aldehyde function is hidden as a bisulfite group to increase the solubility and that can be readily released under physiological conditions (Figure 6a).

These two compounds, initially investigated as veterinary drugs for their capability to inhibit feline coronavirus (FCoV) 3CL protease and proposed for the treatment of feline infectious peritonitis,^28^ were repurposed as new anti-COVID-19 agents, showing potent inhibitory activity on M^PRO^. Specifically, GC-373 exhibited an IC_50_ value against SARS-CoV-2 M^PRO^ of 0.40 ± 0.05 μM, while the prodrug GC-376 produced slightly higher inhibition with an IC_50_ of 0.19 ± 0.04 μM and *k*_i_ = 0.094 μM (*K*_inact_/*k*_i_ = 2.84 × 10^4^ M^–1^ s^–1^).^31,32^ X-ray studies performed on both compounds in complex with SARS-CoV-2 M^PRO^ showed an identical binding mode of the ligands (for GC-373 PDB code 6WTK; for GC-376 PDB code 6WTJ), confirming the ability of the bisulfite adduct of GC-376 to rapidly release the aldehydic function. To gain insight into the mechanism of inhibition and the binding mode of this class of compounds, the crystal structure of GC-376 in complex with the target protein is shown in Figure 6b, highlighting the key structural components responsible for the inhibition: the aldehydic warhead (P1′) forms a covalent hemithioacetal adduct with the cysteinyl −SH within the catalytic cleft in S1′; the leucine P2 inserts itself into the hydrophobic subregion S2 of the enzyme; the recurrent γ-lactame P1 of the substrate is located into the S1 site and forms multiple H bonds with polar amino acids (e.g., His^163^ and Glu^166^); the benzyl group in P3 is projected into the superficial S3/S4 site of the protein, where it forms an H bond with the Glu^166^ backbone.^31^

Further in vitro studies were performed on a model of SARS-CoV-2 infection in Vero E6 cells and confirmed the high inhibitory activity of both GC-373 and GC-376, which exhibited EC_50_ of 1.5 and 0.92 μM, respectively, and low cytotoxicity even at high concentration.^31,32^ The remarkable antiviral effect in the cellular assay (also measured in cells infected with delta and omicron SARS-CoV-2 variants) was associated with the dual effect of inhibition of the host cysteinyl protease cathepsin L, which is involved in the infection process of SARS-CoV-2 into the host cells along with other proteases such as calpains.^33−35^

Inspired by the in vitro results and the previous positive outcomes in animals affected by FCoV, GC-376 was examined in vivo in the K18 hACE2/SARS-CoV-2 transgenic mouse model, which expresses the human angiotensin-converting enzyme type 2 and is affected by SARS-CoV-2. The analysis of the data showed modest benefit in terms of clinical symptoms and survival but an interesting capability to reduce tissue lesions and inflammation in the tested animals.^36^

With the aim of improving the in vitro inhibition effect of GC-376 against M^PRO^, a series of analogues has been designed. From the viewpoint of SAR, the structural modifications have been directed mainly toward the P2 (leucine isopropyl) and the P3 (carboxy-benzyl) moieties; the P1 portion (γ-lactame) and the P1′ aldehyde warhead were retained in view of their pivotal role in the interaction with the target. The most representative compounds were the bisulfite prodrugs **4** and **5** (Figure 6a), which were modified at both P2 and P3 sites (cyclopropyl group and halogen-substituted phenyl, respectively). In vitro inhibition assay proved their better inhibitory activity against M^PRO^ (IC_50_ of 0.07 and 0.08 μM, respectively) and their antiviral effects (EC_50_ of 0.57 and 0.7 μM, respectively) compared to both the lead compound GC-376 and the corresponding parent aldehydes. The interesting potency of compound **4** was confirmed by the analysis of the X-ray complex of M^PRO^ with the desalted form of **4**. As shown in Figure 6c (complex of derivative **4** with SARS-CoV-2 M^PRO^, PDB code 7LCO), introduction of the new structural features contributes to enhance the interaction with the target protein and achieve a better fit into the active site compared to GC-376. The more compact cyclopropyl group on the P2 unit of the ligand is able to penetrate deeper into the S2 pocket of the target; furthermore, introduction of a halogen substituent on the Cbz group (P3 fragment of the ligand) permits one to move this moiety closer to the S3/S4 cleft, while in GC-373 (Figure 6b), the unsubstituted Cbz is mainly directed toward the surface of the protease exposed to the solvent.^29^

To further explore the structural determinants of GC-376 for more selective inhibition of SARS-CoV-2 M^PRO^, UAWJ247 was developed (Figure 6a) by replacing the isopropyl moiety with a phenyl group. The in vitro inhibition assay registered an IC_50_ value of 0.045 μM, very close to that of the parent compound, and a *k*_i_ value of 0.035 μM, confirming a better fitting of the bulkier aromatic portion into the S2 site of the M^PRO^ binding pocket (crystal structure complex with PDB code 6XBH).^30^

Recently, a series of new GC-376 analogues with substantial modifications to the P2 and P3 fragments was synthesized. Among them, compound NK01-63 (coronastat) (Figure 6a) proved to be more active than the lead compound. It showed excellent inhibitory activity against SARS-CoV-2 (IC_50_ = 16 nM), potent antiviral effect in cell assay (EC_50_ = 6 nM in Huh-7^ACE2^ infected cells), and high selectivity against other human proteases. The crystal structure of coronastat in complex with the target protein allowed a better understanding of the drastic improvement in potency compared to the lead GC-376 (PDB code 7TIZ): the trifluoromethyl group on the benzyl moiety provides two additional H bonds with Asn^142^, confirming the importance of the halogen substitution in enhancing the interactions of the ligand–protein complex. These encouraging results prompted the researchers to perform further in vivo tests (mouse models) to evaluate both the activity and the pharmacokinetic profile: coronastat showed no significant toxicity, high metabolic stability, and high concentration in the plasma and lung after both oral and intraperitoneal administration.^37^

**Figure 7:**
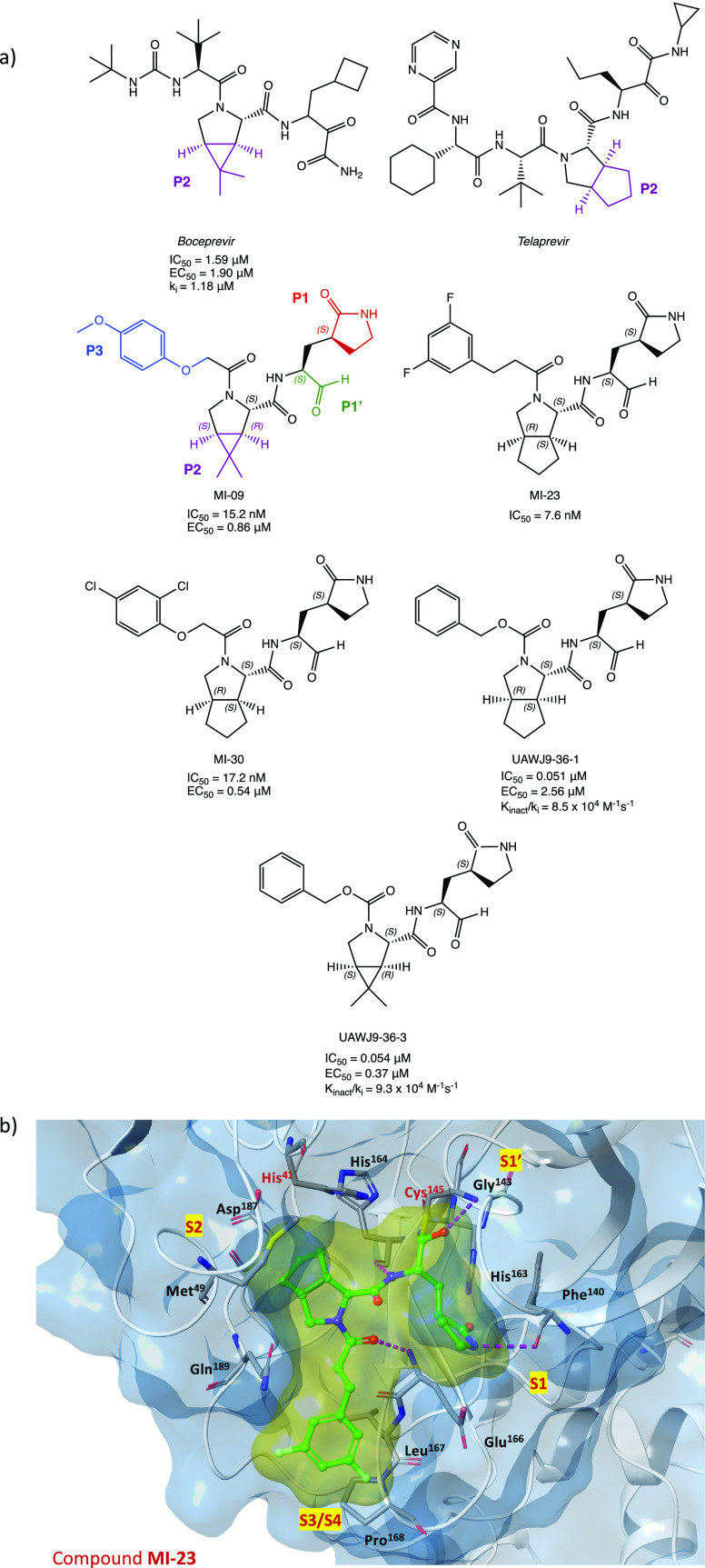
(a) Chemical structures of boceprevir, telaprevir, derivatives MI-09, MI-23, and MI-30, and analogues UAWJ9-36-1 and UAWJ9-36-3. (b) X-ray crystal structure of MI-23 in complex with SARS-CoV-2 M^PRO^ (PDB code 7D3I).^38,40^

Maintaining the focus on the lead compound GC-376, analysis of the crystal structures of M^PRO^ in complex with boceprevir and telaprevir, two drugs with an α-ketoamide group as a warhead (see section 2.2), allowed us to observe the importance of bicyclic proline, which can be suitably accommodated into the S2 pocket of the target protein. The merging of the pharmacophore moieties of GC-376, boceprevir, and telaprevir led to the synthesis of a new series of small molecules with an inhibition effect on SARS-CoV-2 M^PRO^ (Figure 7a). The new derivatives were characterized by the aldehydic P1′ warhead, the γ-lactame as P1 (present in GC-376), (1*R,*2*S,*5*S*)-6,6-dimethyl-3-aza-bicyclo[3.1.0]hexane-2-formamide or (1*S,*3a*R,*6a*S*)-octahydrocyclopenta[*c*]-pyrrole-1-formamide as P2 (from boceprevir and telaprevir, respectively), and a hydrophobic aromatic group as P3. MI-09, MI-23, and MI-30 (Figure 7a), tested in the M^PRO^ biochemical inhibition assay, proved to be the most interesting compounds in the series, exhibiting IC_50_ values in the nanomolar range (15.2, 7.6, and 17.2 nM, respectively). Using X-ray experiments, the most active inhibitor MI-23 was selected to further investigate the binding mode of this class of compounds with SARS-CoV-2 M^PRO^. Figure 7b shows the crystal structure of the complex MI-23/SARS-CoV-2 M^PRO^ (PDB code 7D3I) with the relevant interactions highlighted: the aldehydic carbon interacts with the −SH of the catalytic Cys^145^ at the S1′ site, as expected; P1 is inserted in the S1 region and forms several hydrogen bonds, as observed for the previous compounds; the conformational constraints of the bicyclic proline structure (P2) result in a restricted trans-exo conformation of the system, which determines a deep insertion into the hydrophobic S2 site of the target; the 1-ethyl-3,5-difluorobenzene moiety at P3 occupies the S3/S4 subregion. Further biological studies were conducted on MI-09 and MI-30. Analysis of biological data showed no cytotoxicity against normal cells, remarkable antiviral activity in Vero E6-infected cells (EC_50_ of 0.86 and 0.54 μM, respectively), acceptable PK properties and capability to prevent SARS-CoV-2 infection in vivo (transgenic mice expressing hACE2), and ability to ameliorate lung lesion and inflammation.^38^ Moreover, starting from MI-09 and MI-30, a computational protocol based on QSAR and combined with molecular docking, MD simulations, and free binding energy MM/PBSA was built to design new derivatives with higher inhibition activities on SARS-CoV-2 M^PRO^. The virtual screening highlighted the importance of a bicyclic moiety with a three-membered ring, preferably at P2, and bulky electronegative substituents (ethyl, −CN, −F, and −Br) on the phenyl group at P3.^39^

Similarly, retaining both the aldehyde warhead and the bicyclic fragments, two interesting compounds UAWJ9-36-1 and UAWJ9-36-3 (Figure 7a) were designed and synthesized.^40^

In vitro biological studies of UAWJ9-36 derivatives showed comparable inhibitory activity to GC-376 against both SARS-CoV-2 M^PRO^ (with IC_50_ values of 0.051 and 0.054 μM and *K*_inact_/*k*_i_ = 8.5 × 10^4^ and 9.3 × 10^4^ M^–1^ s^–1^ for UAWJ9-36-1 and UAWJ9-36-3, respectively) and other known human coronaviruses M^PRO^. Antiviral cellular assays performed on Vero E6 and Caco2-ACE2 cell lines infected with SARS-CoV-2 demonstrated the enhanced antiviral activity of UAWJ9-36-3 with EC_50_ = 0.37 and 1.06 μM against the respective cell lines. Moreover, both UAWJ9-36 derivatives showed improved selectivity against host calpains/cathepsins compared to GC-376. X-ray crystal structures of the protein coresolved with both compounds (PDB codes 7LYH and 7LYI for the complexes with UAWJ9-36-1 and UAWJ9-36-3, respectively) confirmed the covalent bonding and the ability of the cyclopentyl-proline and dimethyl-cyclopropyl-proline moieties to insert in the S2 site.^40^

**Figure 8:**
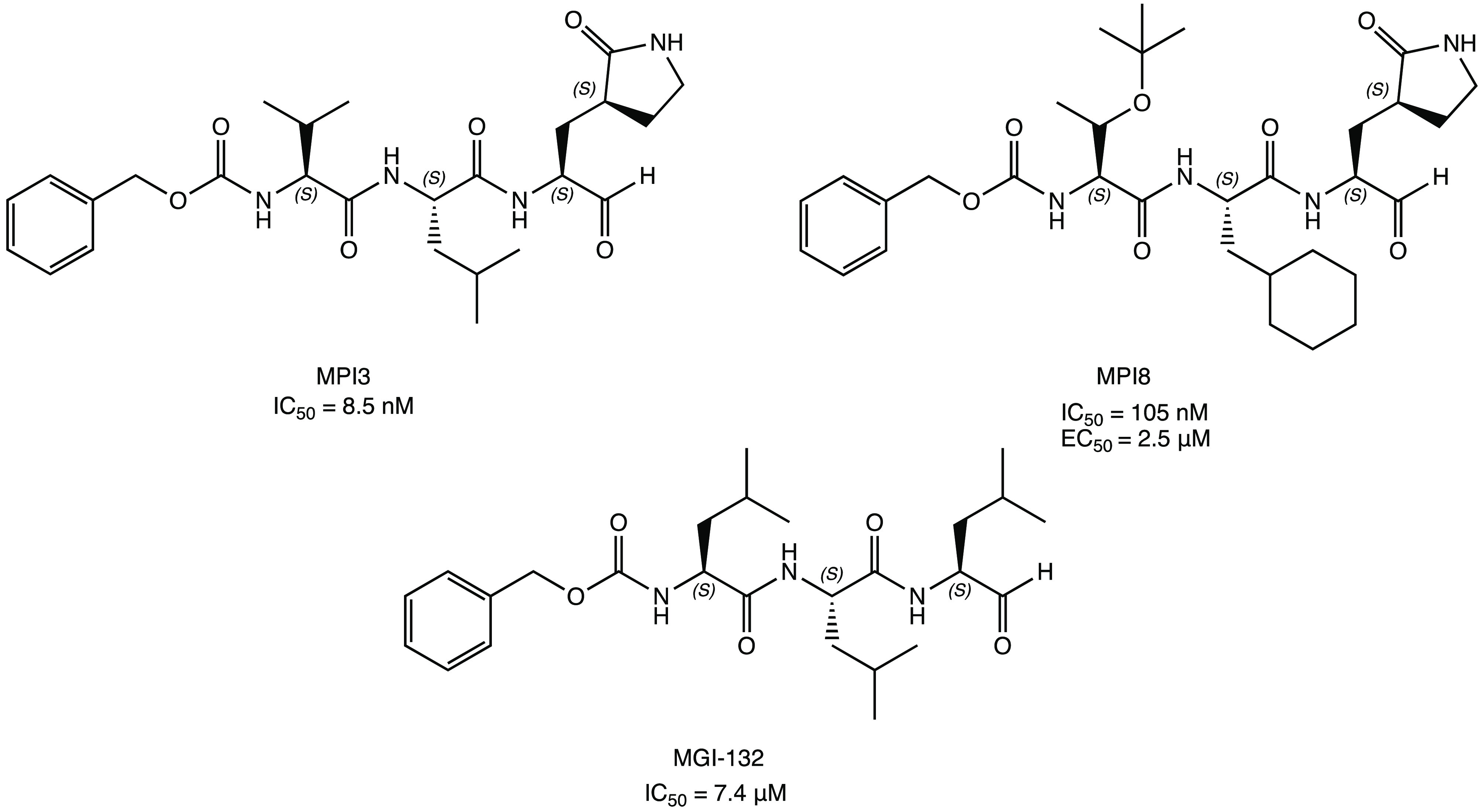
Structures of tripeptidyl inhibitors of SARS-CoV-2 M^PRO^: MPI3, MPI8, and MGI-132.^41,43,44^

Among the SARS-CoV-2 M^PRO^ tripeptidyl inhibitors with an aldehyde moiety as an electrophilic warhead, MPI3 and MPI8 (Figure 8) proved to be the most interesting compounds. These two derivatives feature the recurring 2-oxopyrrolidine side chain at the P1 site and a Cbz group as the N-terminal P3 cap and show remarkable IC_50_ values against SARS-CoV-2 M^PRO^ (8.5 and 105 nM for MPI3 and MPI8, respectively). Despite the higher inhibition by MPI3, its activity in reducing viral infection in in vitro cell models was lower than that of MPI8, which, instead, showed complete inhibition of SARS-CoV-2 infection in Vero E6 and A549/ACE2 cell lines at concentrations of 2.5 and 0.31 μM, respectively. This discrepancy is probably due to the poor stability toward extra/intracellular proteases, which recognize natural amino acids (valine and leucine in the case of MPI3) as substrates.^41^ Further biological studies on MPI8 link the high potency of the tripeptidyl derivative to a dual mechanism of action: inhibition of both SARS-CoV-2 M^PRO^ (IC_50_ = 105 nM) and cathepsin L (IC_50_ = 1.2 nM).^42^

Focusing on the interesting hypothesis of the dual-target inhibition of M^PRO^ and cathepsin L, a well-known proteasome inhibitor, the trileucine peptide MGI-132 (Figure 8), was repurposed. This new compound, structurally similar to MPI3 and MPI8, showed an IC_50_ of 7.4 μM against M^PRO^ and 0.15 nM against cathepsin L as well as a potent antiviral effect on Vero E6 cells infected with SARS-CoV-2.^43,44^

**Figure 9:**
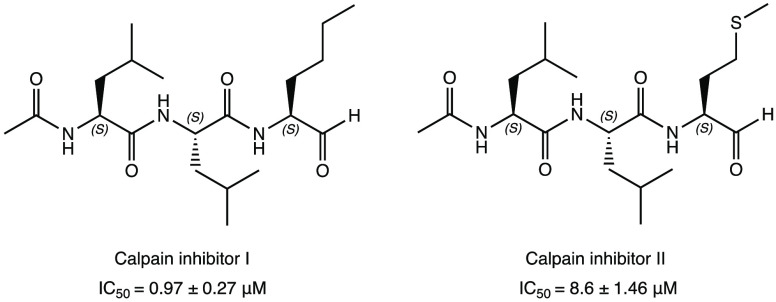
Chemical structures of the repurposed calpain inhibitors I and II as new SARS-CoV-2 M^PRO^ modulators.^30,32,33^

Following the same logic, the calpain/cathepsin L covalent inhibitors I and II were repositioned (Figure 9) as potential SARS-CoV-2 M^PRO^ inhibitors through a comprehensive in vitro screening of investigational/approved inhibitors of the cysteinyl protease. In detail, the tripeptidyl compounds halted the enzymatic activity of SARS-CoV-2 M^PRO^ in vitro at IC_50_ values of 0.97 ± 0.27 and 8.6 ± 1.46 μM, respectively. Calpain inhibitor II was able to block viral progression in the cell infection model (EC_50_ = 2.07 ± 0.76 μM in the primary CPE assay; EC_50_ = 3.70 ± 0.69 μM in the secondary viral yield reduction assay) with no cytotoxicity to normal cells. The analysis of the crystal structure complex of calpain inhibitor II with SARS-CoV-2 M^PRO^ (PDB code 6XA4) confirmed the covalent mechanism of inhibition with the formation of a hemithioacetal adduct. The methionine side chain, deeply inserted into the P1 pocket, could be the starting point for the development of new inhibitors with a dual mechanism of action (M^PRO^ and cathepsins inhibition).^30,32,33^

**Figure 10:**
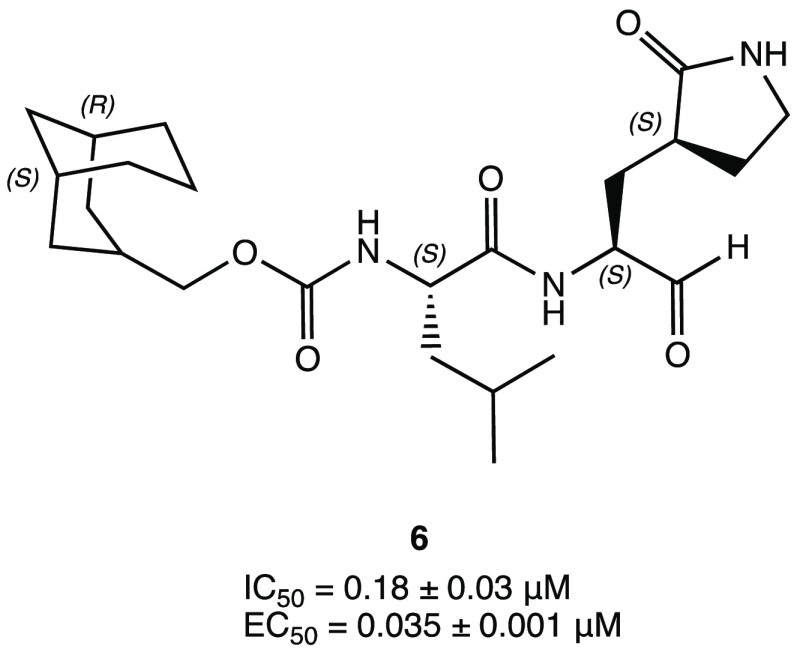
Chemical structure of peptidomimetic compound **6**.^45^

The exploration of the chemical space and structural requirements for the M^PRO^ inhibition has led to the synthesis of a series of dipeptidomimetic compounds with a conformationally constrained cyclohexane moiety as P3. In particular, bicyclic derivative **6** (Figure 10) represented the most interesting compound with an IC_50_ of 0.18 ± 0.03 μM against SARS-CoV-2 M^PRO^ and high antiviral activity (EC_50_ of 0.035 ± 0.001 μM in Vero E6 cells).^45^

#### 2.1.2 Ketone Warhead

The ketone warhead has also been extensively explored in the design of SARS CoV-2 M^PRO^ inhibitors. Among the initial efforts in this direction, the synthesis and biological evaluation of a series of peptidyl acyloxymethyl ketone probes with a covalent inhibition effect toward SARS-CoV-2 M^PRO^ is of significant interest.^46^

**Figure 11:**
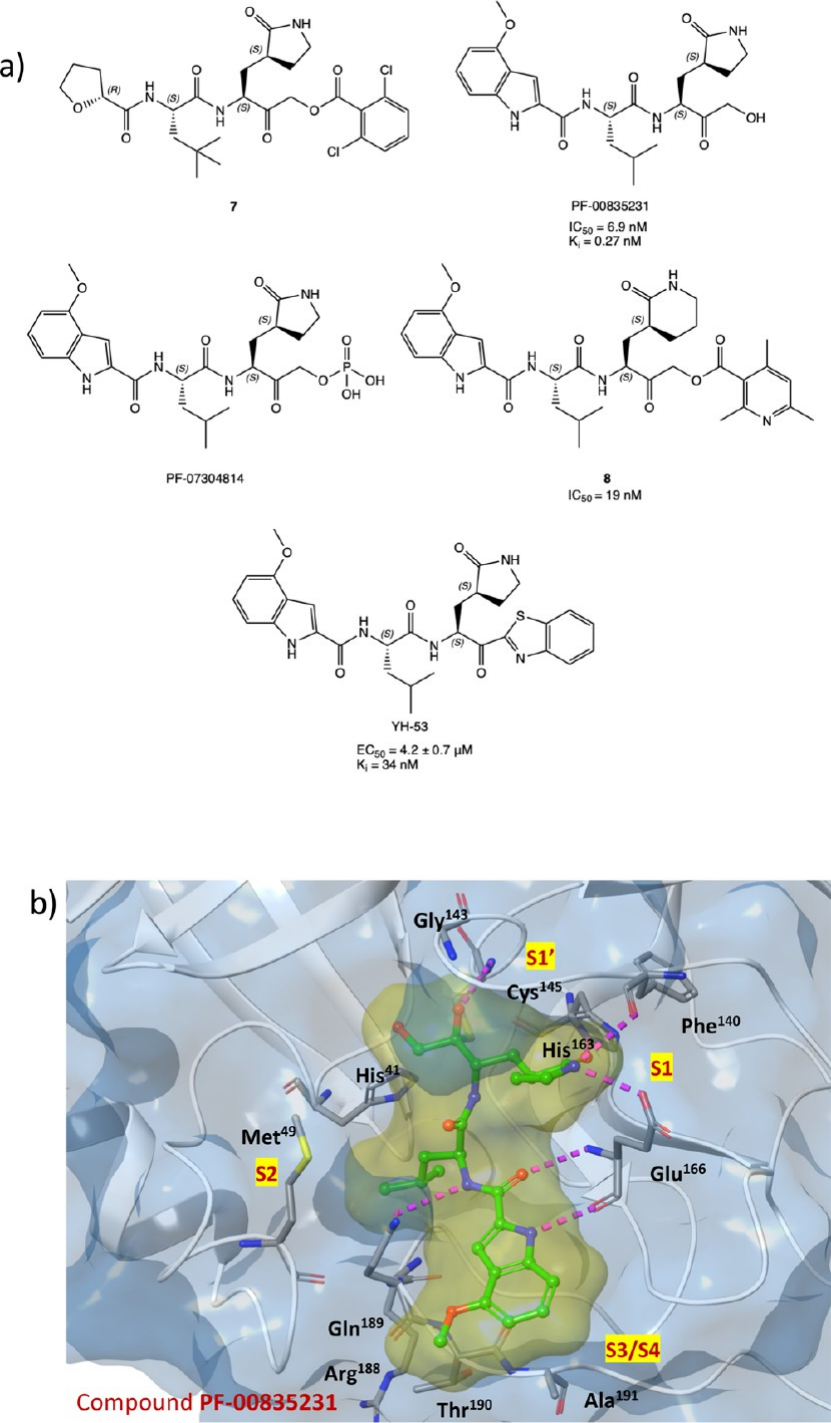
(a) Chemical structures of a series of acyloxymethyl ketone derivatives with SARS-CoV-2 M^PRO^ inhibitory activity. (b) PF-00835231 in complex with SARS-CoV-2 M^PRO^ (PDB code 6XHM).^47,49−52^

Therefore, based on previous findings on a set of M^PRO^ SARS-CoV-1 inhibitors, the α-acyloxymethyl ketone **7** and hydroxymethyl ketone (HMK) PF-00835231 (Figure 11) were synthesized as new M^PRO^ SARS-CoV-2 covalent inhibitors.^47^ In particular, compound PF-00835231 exhibited an interesting *K*_i_ of 0.27 nM and an outstanding IC_50_ of 6.9 nM against the target protein. To gain insight into the binding mode and mechanism of action, PF-00835231 was cocrystallized in a covalent complex with SARS-CoV-2 M^PRO^ (Figure 11b, PDB code 6XHM), highlighting the key contacts with the target: the carbonyl group of the HMK warhead (P1′) is covalently bonded to the Cys^145^ of the S1′ site of the binding pocket, forming a tetrahedral carbinol complex, which together with the primary −OH of the HMK group reinforces the covalent interaction by additional H bonds within the S1 cleft (His^41^, Gly^143^), the lactam P1 is positioned into the S1 pocket (as described earlier), the lipophilic leucine side chain at the P2 site of the ligand is inserted into the S2 region, surrounded by hydrophobic amino acids, and the indole moiety makes the complex more stable through van der Waals interactions with the backbones of the 189–191 amino acid residues.^47^ The mechanism of covalent inhibition of SARS CoV-2 M^PRO^ was investigated by classical and QM/MM simulations, underling the importance of the hydroxymethyl group both in binding free energy increase and in the formation of the hemithioacetal adduct. Analysis of in silico data showed a significant inhibition effective of PF-00835231 against M^PRO^ mutant forms.

PF-00835231 was selected for in vitro/vivo biological evaluation, demonstrating appropriate physicochemical and pharmacokinetic properties for intravenous continuous infusion, similar/higher potency compared with other peptidomimetic SARS CoV-2 M^PRO^ inhibitors (as GC-376) or other anti-SARS CoV-2 agents (remdesivir), and low cytotoxicity and elevated tolerability in an in vitro model of human airway epithelium (HAEC).^47,48^ Further development of PF-00835231 into the phosphate pro-drug PF-07304814 (Figure 11) demonstrated comparable antiviral and M^PRO^ inhibitory activity but better solubility and pharmacokinetic in in vivo animal models; these more favorable features supported the progression of PF-07304814 in clinical trials (ClinicalTrials.gov Identifiers NCT04627532 and NCT04535167).^49^

To explore the range of substitution tolerated for maintaining M^PRO^ inhibitory activity, the α-acycloxymethyl ketones were redesigned by replacing the pentacyclic glutamine mimic lactam with a six-membered one and inserting a heteroaromatic portion as an acyloxy group. The most interesting compound **8** (Figure 11), with a pyridyl moiety, showed a notable inhibition of M^PRO^ (IC_50_ = 19 nM), excellent SARS-CoV-2 replication inhibition, and remarkable plasma and metabolic stability. The capability of **8** to covalently block the target has been confirmed by cocrystallization experiments (PDB code 7MBI).^50^

With a unique P1′-benzothiazolyl ketone moiety, YH-53 (Figure 11a), previously developed as a SARS-CoV-1 M^PRO^ blocker, also proved capable of reversibly inhibiting SARS-CoV-2 M^PRO^ with a *K*_i_ of 34 nM and to halt SARS-CoV-2 infection in vitro (EC_50_ = 4.2 ± 0.7 μM) with a high safety index and low cytotoxicity. X-ray experiments performed with the complex YH-53-SARS-CoV-2 M^PRO^ (PDB code 7E18) confirmed the formation of the covalent adduct and the ability of the heteroatoms of the benzothiazolyl fragment at P1′ to form additional interactions within the catalytic binding site.

In vivo pharmacokinetic studies in animal models highlighted that the introduction of the lipophilic benzothiazole group particularly affected the cLogP value (2.37), suggesting high cell penetration in the GI tract and excellent permeability. In contrast to the almost complete absorption, the recorded bioavailability was very low (3.6%); this issue has been ascribed to the pseudopeptide structure, responsible for high first-pass metabolism in the intestine and liver after oral administration.^51,52^

**Figure 12:**
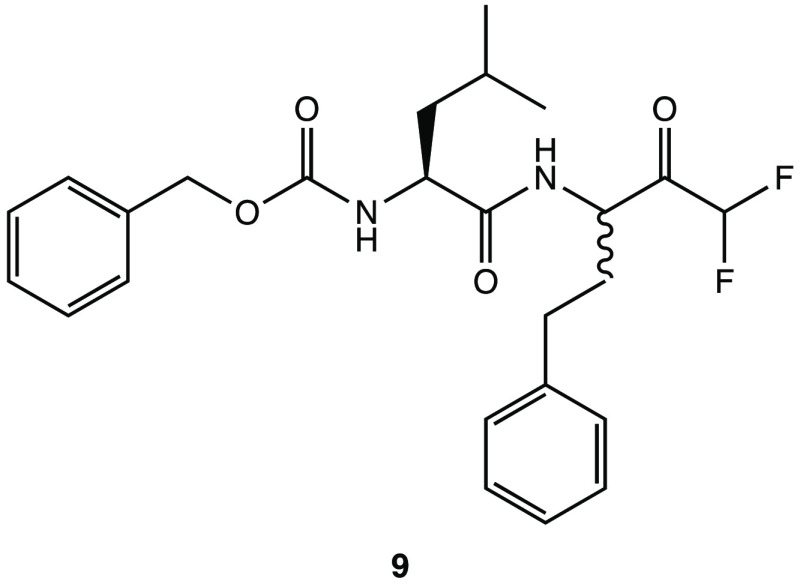
Structure of α,α-difluoromethyl ketone **9**.^53^

Introduction of halogen atoms in the α position of a carbonyl atom also seemed to be an interesting way to increase the reactivity against the −SH catalytic amino acid of the viral protease. In this light, the pseudodipeptide **9** (*Z*-Leu-Homophe-CHF_2_, Figure 12) bearing an α,α-difluoromethyl ketone as an electrophilic warhead showed efficient interactions with SARS-CoV-2 M^PRO^ in in silico simulations. In particular, the two fluoride atoms were capable to form H–halogen bonds with key residues in the catalytic pocket of S1′ (Leu^141^, Ser^144^, and Cys^145^).^53^

**Figure 13:**
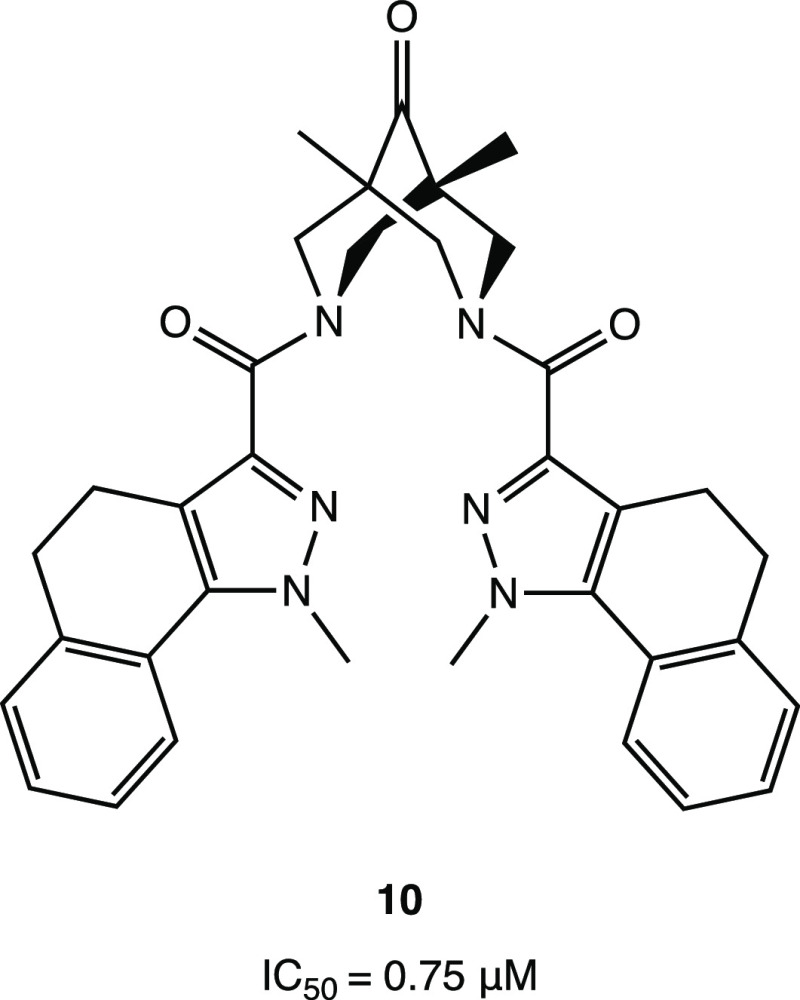
Bispidine compound **10**, a representative example of a nonpeptidomimetic ketone as a SARS-CoV-2 M^PRO^ inhibitor.^54^

Interestingly, an example of a nonpeptidomimetic covalent M^PRO^ inhibitor is represented by the uncommon bispidine-based ketone **10** (Figure 13), which showed noteworthy inhibitory activity against SARS-CoV-2 M^PRO^ (IC_50_ of 0.75 μM). Docking simulations pointed out the central ketone group as the electrophilic center for the covalent bond with the catalytic cysteine.^54^

#### 2.1.3 α-Ketoamide Warhead

**Figure 14:**
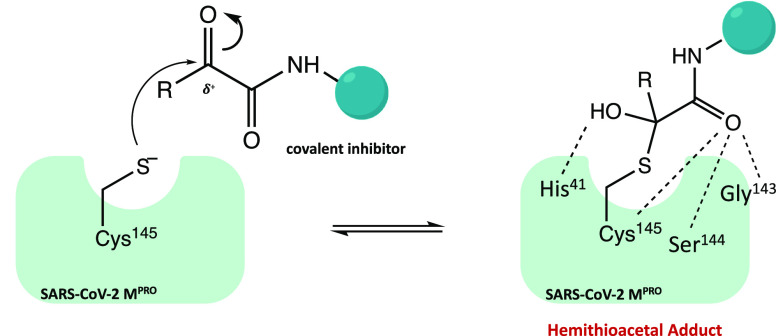
General mechanism of action of SARS-CoV-2 M^PRO^ α-ketoamide inhibitors.

Among the α-ketoamide inhibitors, the carbonyl moiety is also crucial for the formation of a reversible covalent bond with the key cysteine residue of SARS-CoV-2 M^PRO^. In addition to the covalent adduct with the catalytic Cys^145^, the α-ketoamide moiety forms additional noncovalent H bonds with the amino acids of the active site via the carbonyl oxygen and the −OH of the hemithioacetal.^55^ In detail, the nucleophilic attack by the cysteinyl −SH on the α-carbonyl leads to the formation of a hemithioacetal adduct. The hydroxy group of the hemithioacetal gives a hydrogen bond to His^41^, while the oxygen of the carboxamide moiety accepts hydrogen bonds from the main-chain amides of Gly^143^, Cys^145^, and Ser^144^ (Figure 14).^14,56,57^

In accordance with the potential covalent inhibitory effect, the α-ketoamide warhead has been extensively used to develop new SARS-CoV-2 M^PRO^ inhibitors.

**Figure 15:**
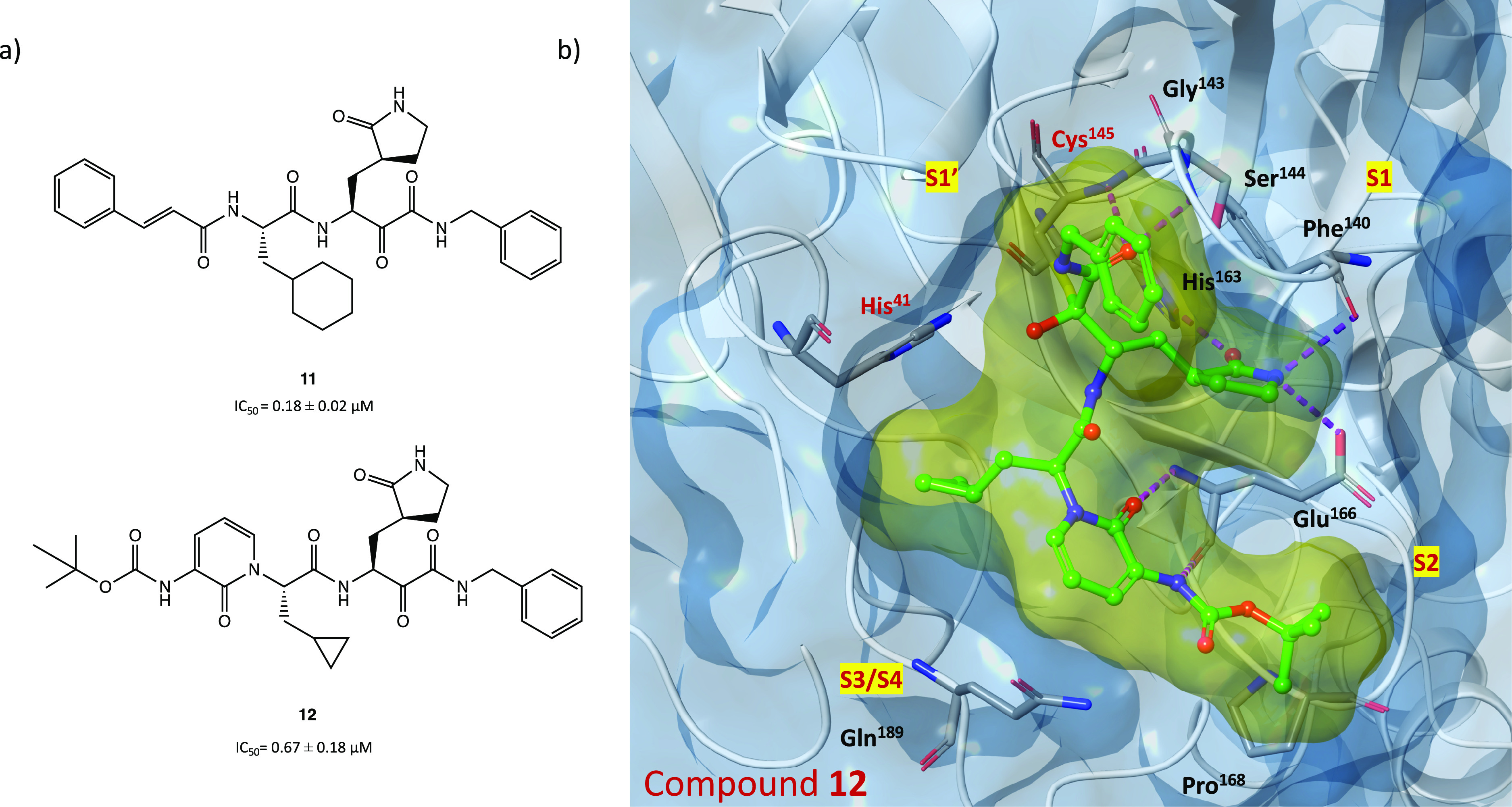
(a) Chemical structures of α-ketoamide derivatives **11** and **12**. (b) X-ray structure of SARS-CoV-2 M^PRO^ in complex with compound **12** (PDB code 6Y2F).^14^

In this context, a new promising series of α-ketoamide compounds with a broad spectrum of activity against the major proteases of enteroviruses, α-coronaviruses, and β-coronaviruses was developed.^14,55^ This class of derivatives is characterized by an α-ketoamide moiety as a warhead, a 5-membered γ-lactam ring at the P1 site (also described for aldehyde and ketone warheads), and a hydrophobic/alkylic group at the P2 site, such as cyclopropyl or cyclohexyl. Studies on viral protease inhibition and antiviral activity have shown that compound **11** (Figure 15a), a previously developed SARS-CoV-1 M^PRO^ inhibitor (IC_50_ = 0.71 ± 0.36 μM), can also effectively inhibit SARS-CoV-2 M^PRO^ (IC_50_ = and 0.18 ± 0.02 μM).^14,55^

Starting from the α-ketoamide **11**, as a lead compound, to optimize the selective inhibition of SARS-CoV-2 M^PRO^, the derivative **12** was obtained (Figure 15a). With the aim of improving the pharmacokinetic parameters, a pyridone ring was inserted and the cinnamoyl and cyclohexyl moieties were replaced by a hydrophobic *tert*-butyloxycarbonyl group and a smaller cyclopropyl group, respectively.^14^ In vitro biochemical assays showed that compound **12** selectively inhibited SARS-CoV-2 M^PRO^ with IC_50_ = 0.67 ± 0.18 μM and blocked SARS-CoV-2 viral infection in human Calu-3 cells with remarkable EC_50_ values in the range of 4–5 μM. Moreover, in vivo studies conducted in CD-1 mice highlighted favorable pharmacokinetic properties and positive tropism of the compound in the lung, the primary target of COVID-19.^14^

Further in silico studies (molecular docking and molecular dynamics simulations) showed that **12** is able to bind efficiently to the catalytic site of the protease. In Figure 15b (crystal structure of SARS-CoV-2 M^PRO^ in complex with compound **12**, PDB code 6Y2F), the X-ray experiment confirms the formation of the covalent adduct (Figure 14): the α-ketoamide group enhances the interaction in the S1′ cleft thanks to the H bonds with His^41^, Gly^143^, Ser^144^, and Cys^145^, the S1 site accommodates the glutamine surrogate γ-lactam ring, the key fragment amidopyridone interacts with Glu^166^ via two H bonds, and the *tert*-butyl moiety is inserted into the S2 pocket.^14,58,59^

**Figure 16:**
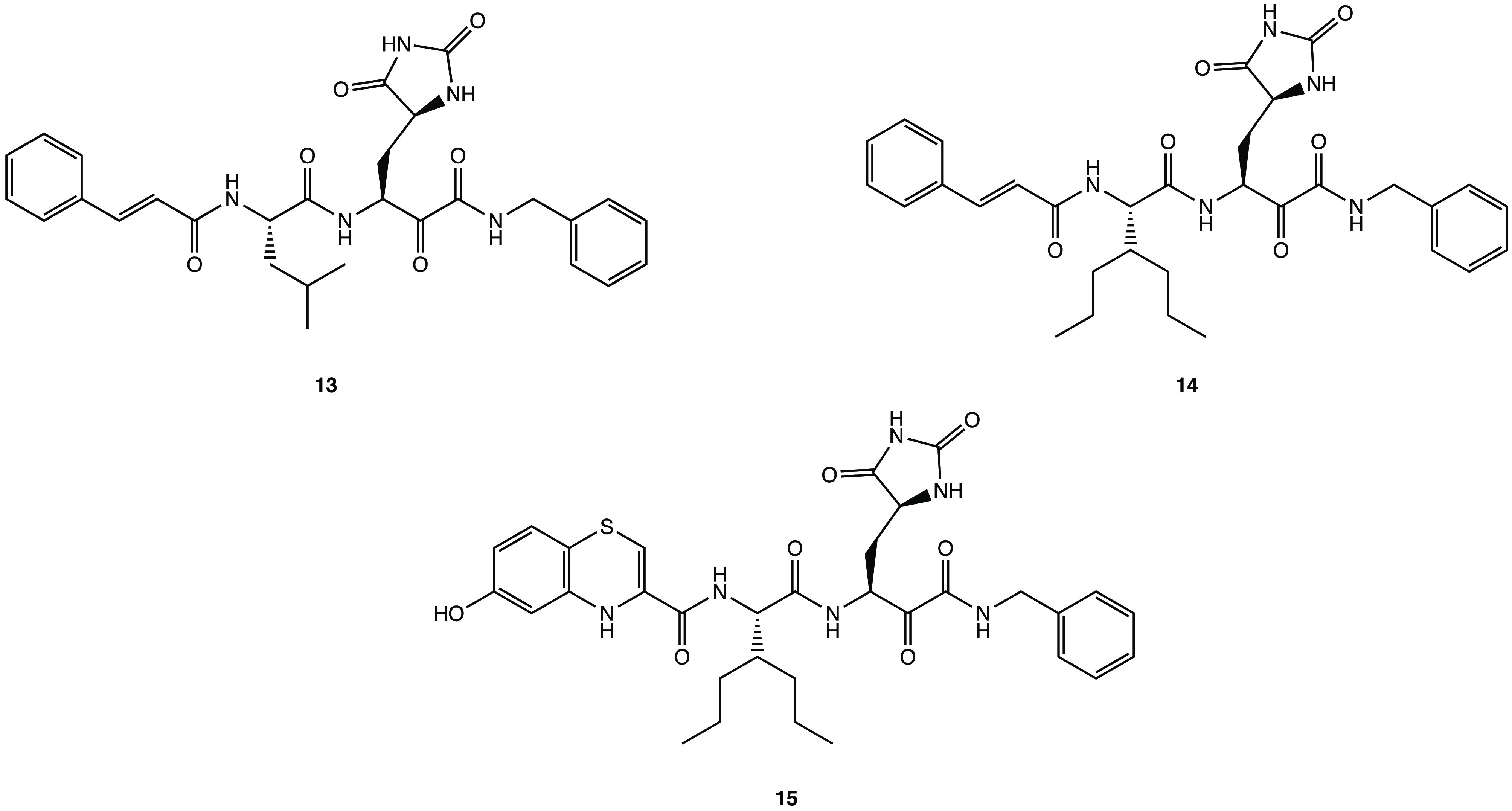
Chemical structures of hydantoin derivatives **13–15.**(60)

In addition, starting from derivative **11**, an in silico structural optimization process was carried out. The obtained compounds **13**–**15** were characterized by replacement of the lactam moiety in P1 by a hydantoin one, which increased the predicted binding affinity with SARS-CoV-2 M^PRO^ through additional H-donor/acceptor bonds (Figure 16).^60^

**Figure 17:**
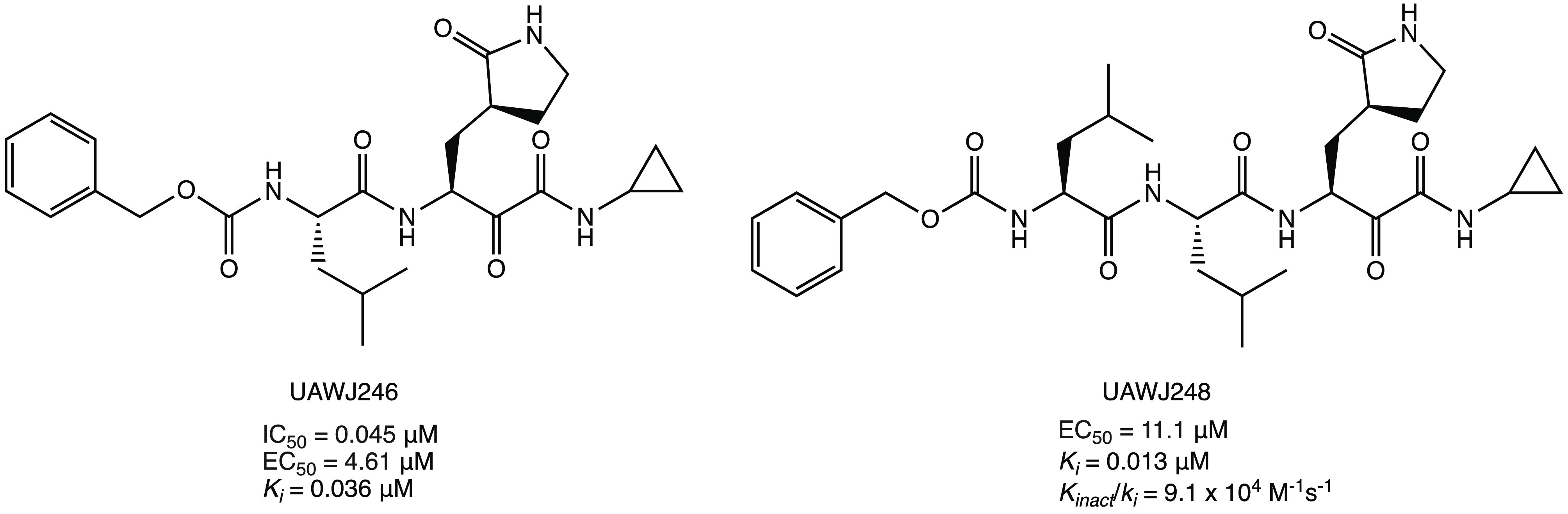
Chemical structures of GC-376 α-ketoamide analogues UAWJ246 and UAWJ248.^30^

Using GC-376 as the lead compound, the two α-ketoamide analogues UAWJ246 and UAWJ248 were developed (Figure 17) to investigate the effects of the aldehyde warhead replacement on the binding affinity with SARS-CoV-2 M^PRO^. Nevertheless, the biological in vitro screening showed comparable inhibitory activity against M^PRO^ with the parent compound (IC_50_ = 0.045 μM and *k*_i_ = 0.036 μM for UAWJ246; EC_50_ = 11.1 μM and *k*_i_ = 0.013 μM for UAWJ248).^30^

**Figure 18:**
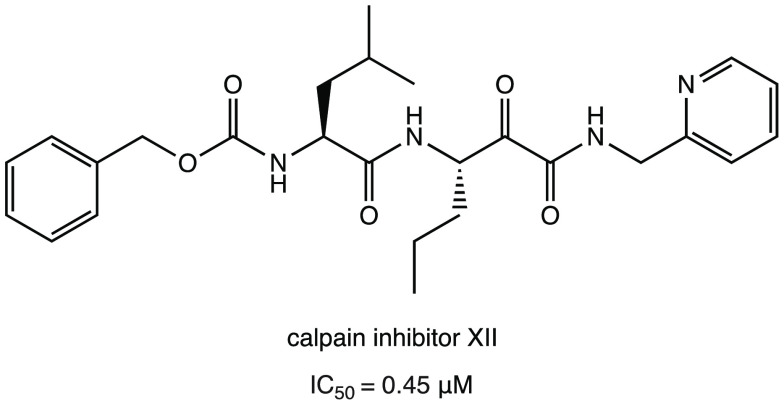
Chemical structure of calpain inhibitor XII.^30,32,40^

As described in the previous paragraph for calpain inhibitors I and II, the calpain inhibitor XII (Figure 18) is another interesting covalent inhibitor with an α-ketoamide warhead. Compared to calpain inhibitors I and II, calpain inhibitor XII showed higher antiviral activity in the cellular model (EC_50_ = 0.49 ± 0.18 μM in the primary CPE assay and EC_50_ = 0.78 ± 0.37 μM in the secondary viral yield reduction assay) and interesting SARS-CoV-2 M^PRO^ inhibitory activity in the submicromolar range (IC_50_ = 0.45 μM).^30,32,40^

**Figure 19:**
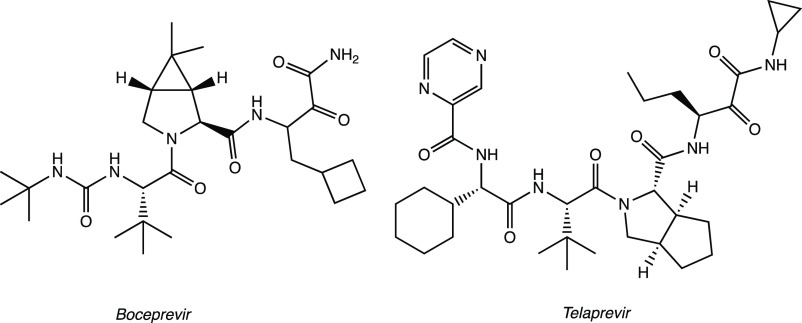
FDA-approved α-ketoamide drugs: boceprevir and telaprevir.

On the other hand, an in silico repurposing study of FDA-approved antiviral drugs as covalent inhibitors of SARS-CoV-2 M^PRO^ led to the identification of boceprevir and telaprevir (Figure 19) as promising drugs with an α-ketoamide warhead. Both X-ray experiments (PDB code 6ZRU and 6ZRT) and docking studies confirmed the ability of the two drugs to form covalent adducts with Cys^145^.^61−63^ In vitro biochemical assays showed significant activity of boceprevir against SARS-CoV-2 M^PRO^ with an IC_50_ of 1.59 μM.^61^

Noteworthy, several in silico analyses have been carried out to design new potential inhibitors that offer intriguing ideas for future developments. The most promising of these are discussed in the following sections.

**Figure 20:**
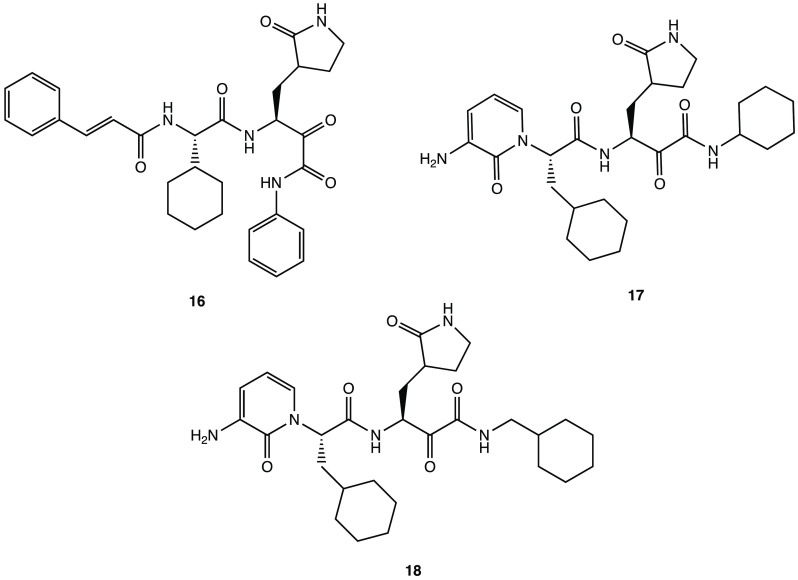
Chemical structures of potential SARS-CoV-2 M^PRO^ inhibitors **16–18.**(59)

The application of various computational approaches (QSAR modeling, pharmacophore modeling, molecular docking, and molecular dynamics simulations) enabled the discovery of a novel series of α-ketoamide compounds such as derivatives **16**–**18** (Figure 20). Analysis of the in silico data revealed that all three molecules interacted efficiently and stably with SARS-CoV-2 M^PRO^ (similar to the reference compound **12**) and also exhibited favorable pharmacokinetic and toxicological profiles.^59^

**Figure 21:**
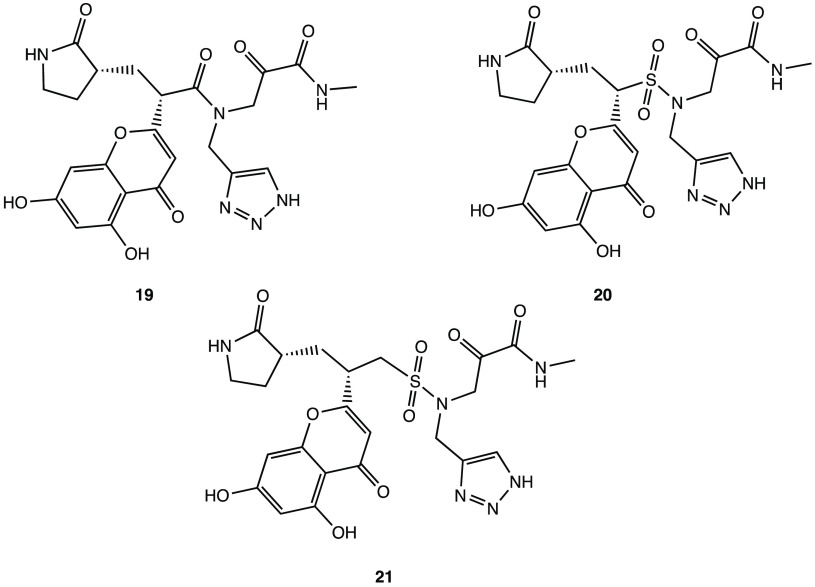
In silico designed SARS-CoV-2 M^PRO^ inhibitors **19–21.**(64)

Using a new advanced computational approach with a molecular covalent docking analysis, derivative **19** (Figure 21) emerged as one of the most promising molecules targeting SARS-CoV-2 M^PRO^. According to the binding pose, the covalent complex **19**/SARS-CoV-2 M^PRO^ was mainly stabilized by hydrogen bonds: the carbonyl group of the α-ketoamide, the triazole ring, and the oxygen in the β-lactam ring formed H bonds with Leu^141^, Gly^143^, Glu^166^, and His^41^. Moreover, to optimize the lead compound, a sulfonic group was also introduced to enhance the polarity, leading to compounds **20** and **21** (Figure 21), with improved in silico affinity for the binding pocket of the target protein.^64^

**Figure 22:**
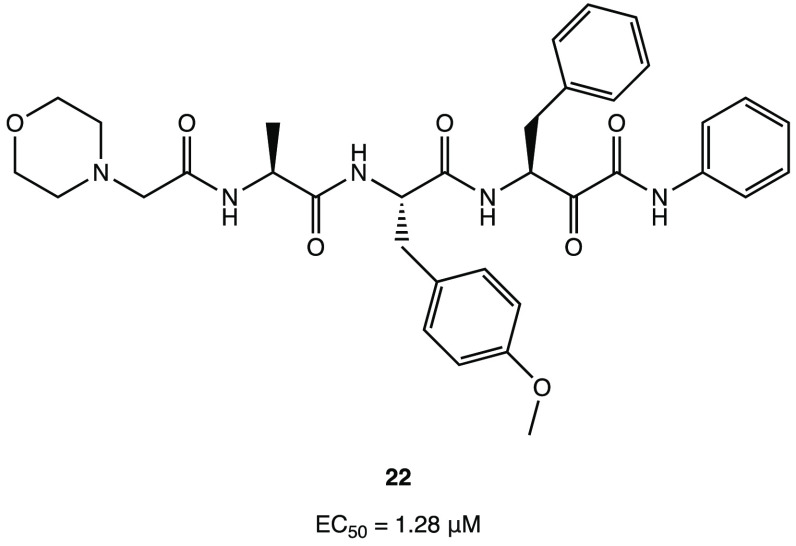
Chemical structure of the anticancer compound **22** with activity on SARS-CoV-2.^65^

Among the series of novel α-ketophenylamide derivatives as anticancer agents, derivative **22** (Figure 22) was selected as a representative compound to be tested in the SARS-CoV-2 cell infection assay, and it was found to actively block viral infection in in vitro models (EC_50_ of 1.28 μM in the plaque reduction assay). Despite the lack of target inhibition data, the antiviral effect exhibited in the cell assay could be attributed to the inhibition of M^PRO^ due to its structural similarity with the above-mentioned α-ketoamide derivatives.^65^

#### 2.1.4 Michael Acceptor Group as a Warhead

**Figure 23:**
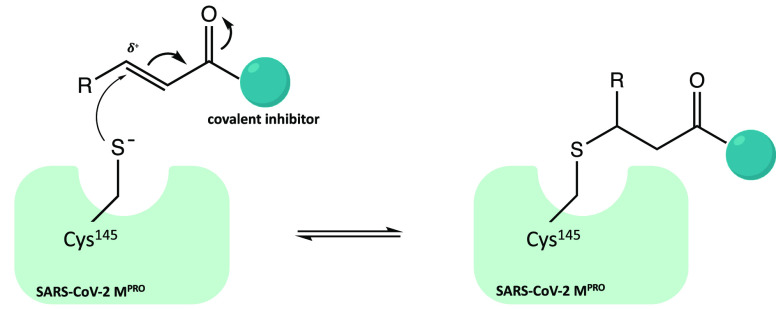
General mechanism of inhibition conjugate systems (α,β-unsaturated carbonyl warhead is reported as an example).

Similar to carbonyl and α-ketoamide warheads, Michael acceptor groups have also been extensively used in the development of covalent inhibitors for cysteinyl enzymes.^16−18^ Michael acceptor groups (such as α,β-unsaturated carbonyl, vinyl nitriles, vinyl sulfonamides, etc.) offer potential advantages for target inhibition compared to other warheads; the Michael acceptor groups inhibit the enzymes via conjugate addition of the nucleophilic cysteinyl −SH to the electrophilic Cβ of the unsaturated system, producing a nearly irreversible and longer lasting adduct. Figure 23 shows the scheme of covalent inhibition by an α,β-unsaturated carbonyl warhead.

**Figure 24:**
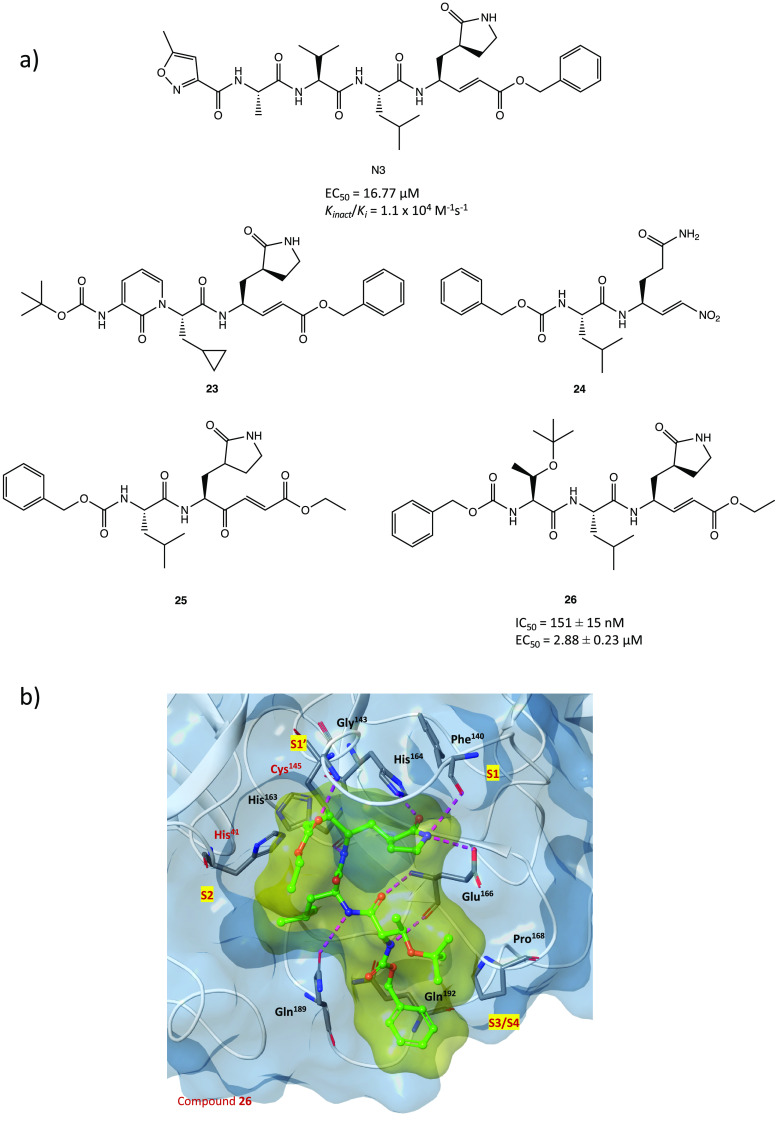
(a) Chemical structures of N3 and analogues **23**–**26**.^15,67−69^ (b) X-ray crystal structure of **26** in complex with SARS-CoV-2 M^PRO^.^69^

N3, a compound previously evaluated for activity against SARS-CoV-1 and MERS-CoV M^PRO^ (Figure 24a), is one of the first peptidomimetic covalent inhibitors of SARS-CoV-2 M^PRO^ with an α,β-unsaturated Michael acceptor group as a warhead. Kinetics and X-ray studies explained the ability of the compound to irreversibly bind the thiol group of Cys^145^ in the M^PRO^ catalytic site, reacting via a conjugate addition mechanism (PDB code 6LU7).^15^ To better understand the irreversible inhibition of N3 at the atomic level, hybrid QM/MM free energy methods were also performed. In detail, the mechanism of action of N3 consists of two steps: formation of the high-energy ion pair Cys^145–^/His^41+^ dyad and subsequent establishment of covalent bonds.^66^

The same in silico approaches were applied to design and computationally test three new N3 analogues **23**–**25** (Figure 24a), obtained by modifying the recognition portion and/or the warhead. Compared to the lead compound N3, they were predicted to possess improved thermodynamic and kinetic properties.^67,68^ Such an approach proves the importance of this type of in silico techniques, especially in the design of new covalent inhibitors.

The N3 analogue **26** (Figure 24a) was identified by in vitro screening of previously described SARS-CoV-1 M^PRO^ inhibitors. It showed promising SARS-CoV-2 M^PRO^ inhibitory activity (IC_50_ = 151 ± 15 nM), ability to block viral infection in Vero E6 cells (EC_50_ = 2.88 ± 0.23 μM), and covalent inhibition of the target protein. As illustrated by the X-ray image of the complex **26**/M^PRO^ (Figure 24b, PDB code 7JT7), the sulfur atom of Cys^145^ forms a covalent bond with the Cβ atom of the vinyl group (Michael addition to the α,β-unsaturated system) while the other portions (the γ-lactam ring, the isoleucine, and the phenyl) are inserted into protein regions S1, S2, and S4, respectively.^69^

**Figure 25:**
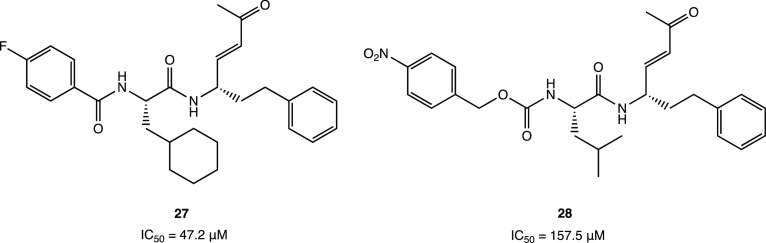
Chemical structures of peptidomimetic irreversible M^PRO^ inhibitors **27** and **28**.^70^

Through a receptor-based virtual screening campaign of an in-house database of cysteine-responsive compounds, two peptidomimetic irreversible M^PRO^ inhibitors **27** and **28** were discovered (Figure 25) with a vinyl-ketone portion as the Michael acceptor group. In vitro biochemical assays revealed promising inhibitory activity with IC_50_ values of 47.2 and 157.5 μM, respectively. Docking and molecular dynamics simulations confirmed the formation of a covalent adduct between the two derivatives and the reactive cysteine, highlighting the importance of the hydrophobic alkyl P2 moieties (cyclohexyl and isobutyl) and the para-substituted aromatic P3 moieties (*p*-F-phenyl and *p*-NO_2_-phenyl) for stable interactions with the target protein.^70^

Recently, however, many nonpeptidomimetic compounds have also been proposed as SARS-CoV-2 M^PRO^ inhibitors, leading to new ideas for exploring new chemical spaces and nuclei.

**Figure 26:**
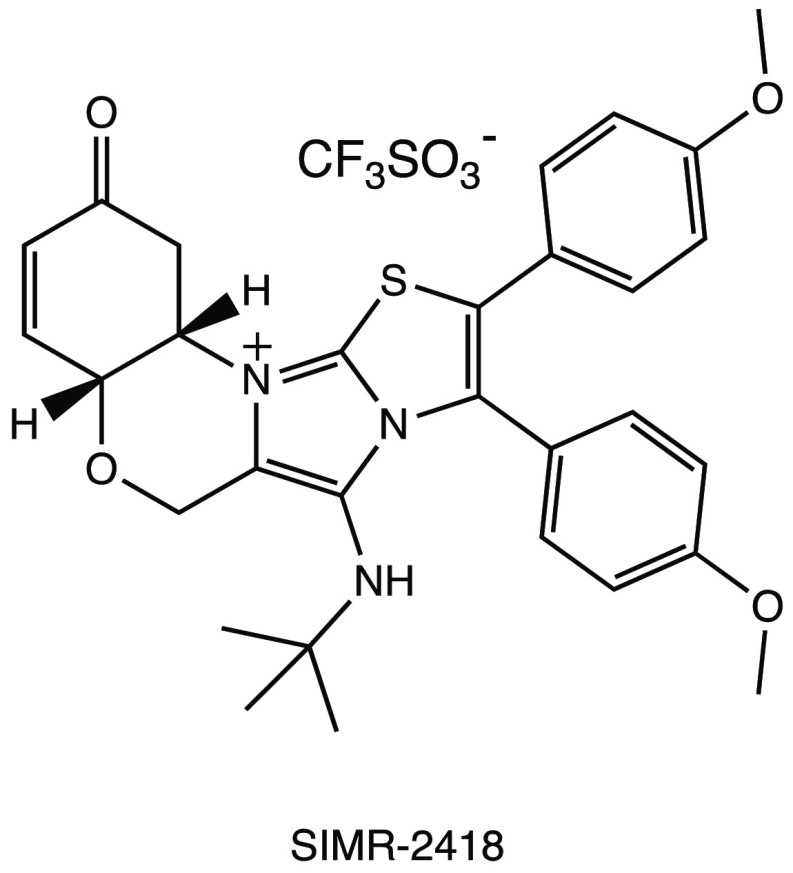
SIMR-2418, a promising nonpeptidomimetic SARS-CoV-2 M^PRO^ inhibitor.^71^

In an in vitro pilot screening of a highly focused compound library to identify new nonpeptidomimetic scaffolds for covalent inhibition of M^PRO^, the derivative SIMR-2418 emerged as one of the most active inhibitors (Figure 26) with an IC_50_ of 20.7 μM against SARS-CoV-2 M^PRO^ and the most favorable ADME properties. Molecular docking and dynamics analyses showed that the unexplored core, based on the fusion between a benzo[*b*][1,4]oxazin-6(5*H*)-one and an imidazo[2,1-*b*]thiazole system, is crucial to achieve the desired orientation in the active site of the target protein. The presence of a cyclohexenedione fragment is essential for the covalent inhibition, while the *tert*-butyl group fits deeply into a hydrophobic cleft.^71^

**Figure 27:**
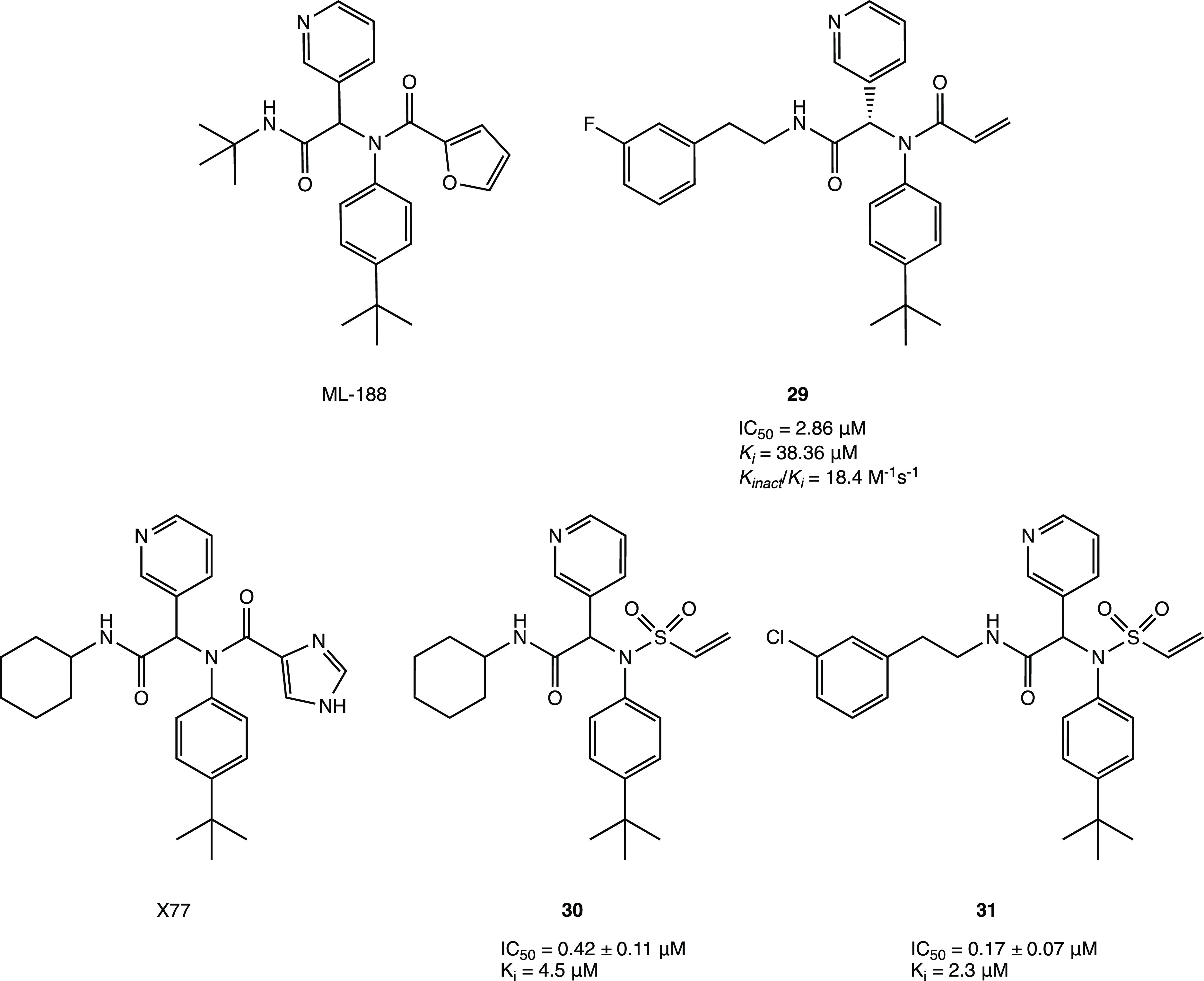
Examples of nonpeptidomimetic SARS-CoV-2 M^PRO^ inhibitors with a covalent mechanism of action.^72,73^

Interestingly, covalentizer, an automated pipeline, could be an effective tool to discover new covalent SARS CoV-2 M^PRO^ inhibitors with a Michael acceptor group. This innovative computational protocol enabled the development of a series of irreversible acrylamide agents starting from the reversible nonpeptidomimetic SARS CoV-1/2 M^PRO^ inhibitor ML188. The most interesting compound, **29** (*S* enantiomer, Figure 27), showed a significant IC_50_ of 2.86 μM (the *R* enantiomer was nearly inactive with an IC_50_ of 86.32 μM). The X-ray crystal structure (PDB code 7NW2) provided important details about the binding mode of **29** in the catalytic pocket of SARS CoV-2 M^PRO^: the conjugate acrylamide moiety, which replaces the furan one, is reactive to the nucleophilic conjugate addition of the SH; the *p*-*tert*-butylphenyl moiety is projected deeply into the hydrophobic S2 pocket; the 3-F-phenetylamide fragment, absent in the lead ML-118, is essential to establish key contacts with the S4 cleft.^72^

Similarly, starting from the analysis of a previously developed noncovalent SARS CoV-2 M^PRO^ inhibitor X77, it was possible to design new covalent agents (Figure 27). The imidazole moiety of X77, located near the catalytic Cys^145^ (PDB code 6W63), was replaced with several conjugated warheads. One of the most interesting compounds, **30** (Figure 27), with an unusual vinyl sulfone moiety, showed an IC_50_ of 0.42 ± 0.11 μM against SARS CoV-2 M^PRO^, an order of magnitude stronger than X77 (IC_50_ = 4.1 μM). The covalent inhibition mechanism was confirmed by both kinetic (ITC, isothermal titration calorimetry) and crystallographic analyses (PDB code 7MLG). Further SAR studies performed with the designed lead compounds allowed the optimization of the interaction with the target; in particular, it was found that the substitution of the cyclohexyl portion with longer chains led to compounds with higher potency compared to **30** (compound **31** showed an IC_50_ of 0.17 ± 0.07 μM, Figure 27).^73^

**Figure 28:**
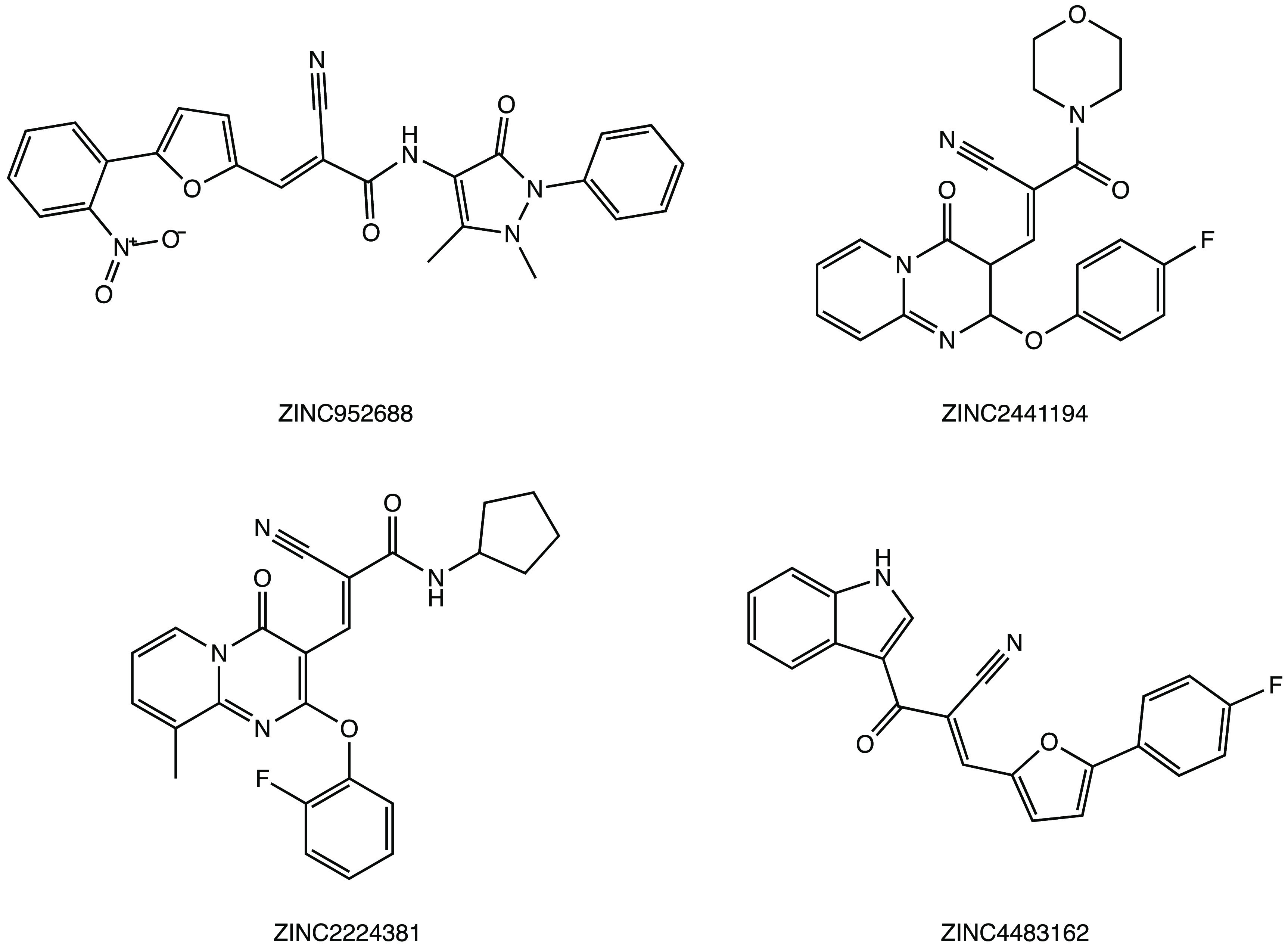
Chemical structures of ZINC database inhibitors of SARS CoV-2 M^PRO^.^74^

Among the unusual Michael acceptor warheads, the acrylonitrile moiety shows great potential. A library of more than 1400 cysteine-focused ligands from the ZINC database was investigated using covalent docking, molecular dynamics studies, and ADMET predictions. The ligands ZINC952688, ZINC2441194, ZINC2224381, and ZINC4483162 (Figure 28) were identified as the most interesting compounds, forming a covalent adduct with the M^PRO^ via conjugate addition at the acrylic double bond.^74^

In addition to the above examples, several nonpeptidomimetic natural products, such as the conjugated curcuminoid and quinonoid/flavonoid systems, have also emerged as interesting lead compounds for the development of novel covalent SARS CoV-2 M^PRO^ inhibitors in extensive in silico/in vitro screening campaigns.

**Figure 29:**
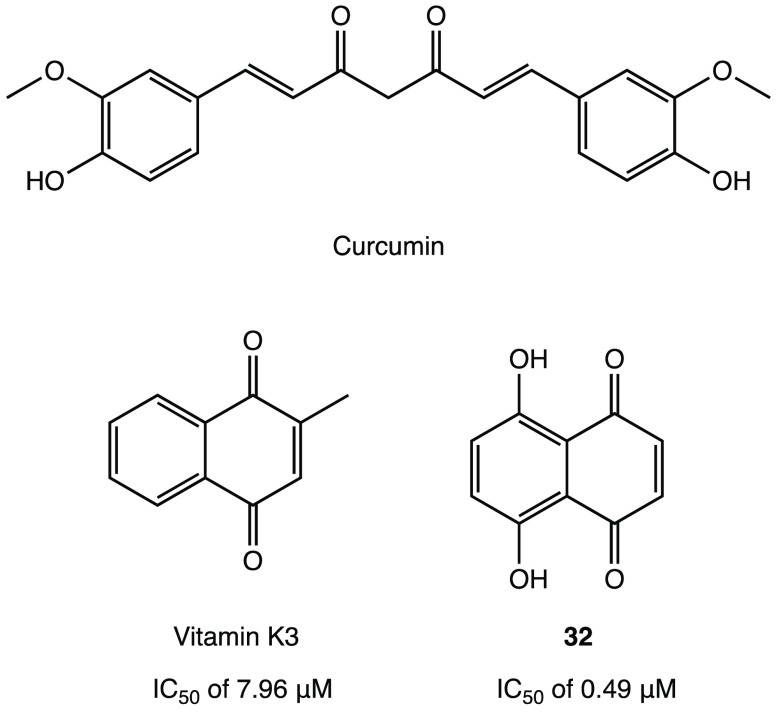
Chemical structures of natural products and analogues as potential SARS-CoV-2 M^PRO^ inhibitors.^75−77^

Thus, curcumin (Figure 29), a natural compound known for its countless biological activities, was found to be a potential covalent inhibitor of SARS-CoV-2 M^PRO^ in an in silico study.^75^ Indeed, thanks to its α,β-unsaturated carbonyl moiety, it could react with Cys^145^ through a Michael addition, as predicted by molecular docking and dynamics studies.^75^

As an example of quinonoid analogues, in a preliminary in vitro study, vitamin K3 (menadione, a synthetic form of vitamin K, Figure 29) proved to inhibit SARS-CoV-2 M^PRO^ with an IC_50_ of 7.96 μM.^76^ Further optimization led to the development of vitamin K3 analogue **32** (5,8-dihydroxy-1,4-naphthoquinone, Figure 29), which showed a 10-fold increase in inhibitory activity compared to the parent compound (IC_50_ of 0.49 μM against SARS-CoV-2 M^PRO^). In silico and kinetic studies with both vitamin K and **32** confirmed the capability to irreversibly inhibit M^PRO^ by forming a covalent bond via conjugate addition at the quinone core.^77^

**Figure 30:**
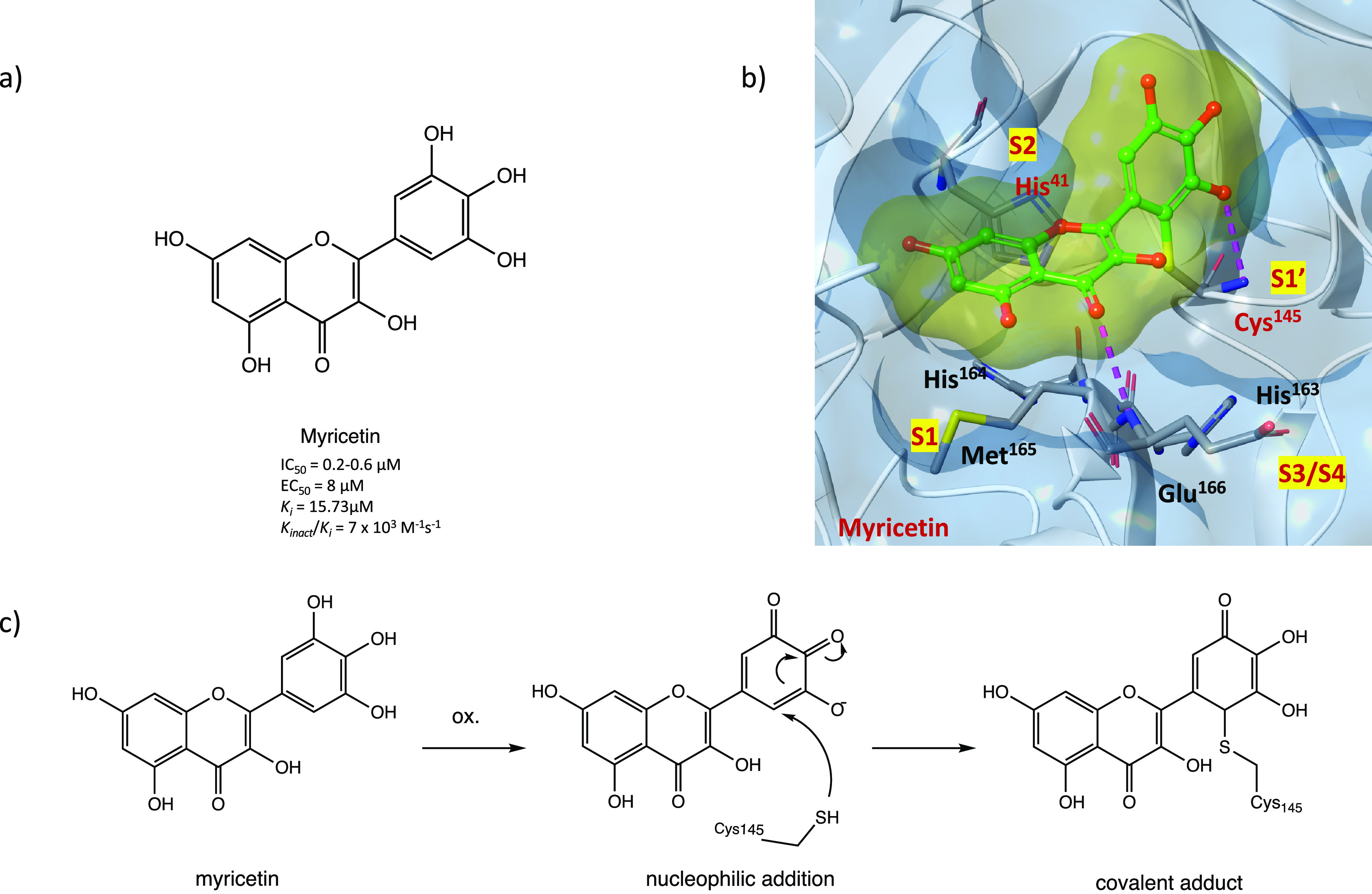
(a) Chemical structure of the flavonoid compound myricetin. (b) X-ray crystal structure of the covalent complex between myricetin and SARS-CoV-2 M^PRO^ (PDB code 7B3E). (c) Hypothetical mechanism of action of myricetin in inhibiting SARS-CoV-2 M^PRO^.^44,79^

Other examples of natural compounds containing Michael acceptor fragments include the flavonoids. Indeed, many virtual and in vitro screens have identified flavonoid compounds as potential covalent SARS-CoV-2 M^PRO^ inhibitors. Myricetin, with its novel hidden electrophilic pyrogallic portion (Figure 30a), is one of the best characterized.^78^ The enzymatic inhibition assay confirmed its great potency with an IC_50_ of 0.2–0.6 μM against M^PRO^. The solved X-ray crystal structure in complex with SARS-CoV-2 M^PRO^ showed that the C6′ of myricetin is covalently linked to the catalytic Cys^145^ (Figure 30b, PDB code 7B3E).^44,79^

Considering the structure of myricetin, the irreversible inhibition could be unexplained. However, a preliminary in vivo oxidative step was proposed to explain the biological activity (Figure 30c): after the oxidation, the pyrogallol fragment is converted to an *o*-quinone, which could function as an α,β-unsaturated carbonyl group (as in the *p*-quinone group of vitamin K3, see above). The sulfur atom of Cys^145^ can attack *o*-quinone, and the resulting prototropic equilibrium could lead to the formation of the covalent adduct.^79^

Further in vitro cellular assays highlighted the capability of myricetin to block SARS-CoV-2 infection in Vero E6 cells with an EC_50_ value of 8 μM.^79^ Moreover, in an in vivo model of lung injury in mice it was shown to inhibit the infiltration of inflammatory cells and the production of pro-inflammatory cytokines (e.g., IL-6, TNF-α), thereby alleviating the overall inflammation.^80^ These findings prompted the scientific community to develop new inhibitors based on the myricetin scaffold.

**Figure 31:**
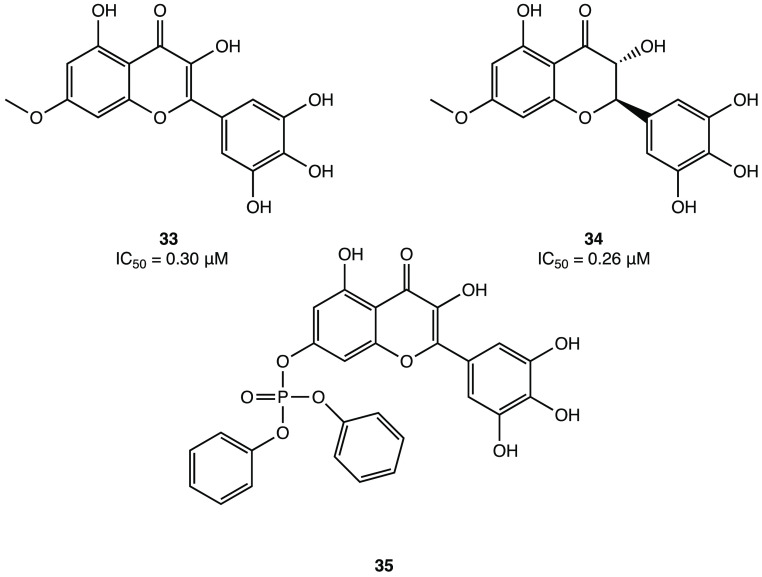
Chemical structures of promising myricetin analogues as covalent SARS-CoV-2 M^PRO^ inhibitors.^79^

Indeed, by inserting a *p*-OCH_3_ at the C7 of myricetin core, derivatives **33** and **34** (Figure 31) were obtained with improved potency compared with the parent compound (IC_50_ = 0.30 and 0.26 μM, respectively). In addition, insertion of a phosphonate group to the 7-OH of myricetin led to compound **35** (Figure 31), which exhibited the highest inhibitory activity against SARS-CoV-2 M^PRO^, demonstrating the consistency of the prodrug strategy for further development.^79^

### 2.2 α-Haloacetamide Warhead

**Figure 32:**
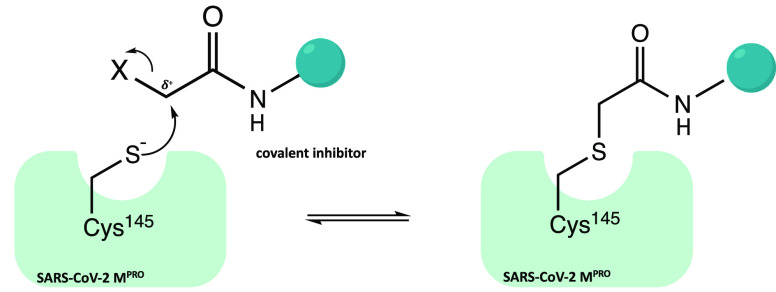
General mechanism of action of an α-haloacetamide warhead.

Alkyl halogens, especially when in the α position to a carbonyl group, have been extensively exploited to design covalent inhibitors because of their intrinsic reactivity toward nucleophiles. In this light, we report here recent efforts to develop new covalent SARS-CoV-2 M^PRO^ inhibitors with α-haloacetamide, α,α-dihaloacetamide, and α,α,α-trihaloacetamide as reactive groups.^73,81^ As shown in Figure 32, the general mechanism of inhibition for this class of compounds consists of a nucleophilic attack of the cysteinyl −SH on the C–X and subsequent formation of the irreversible S–C bond.

**Figure 33:**
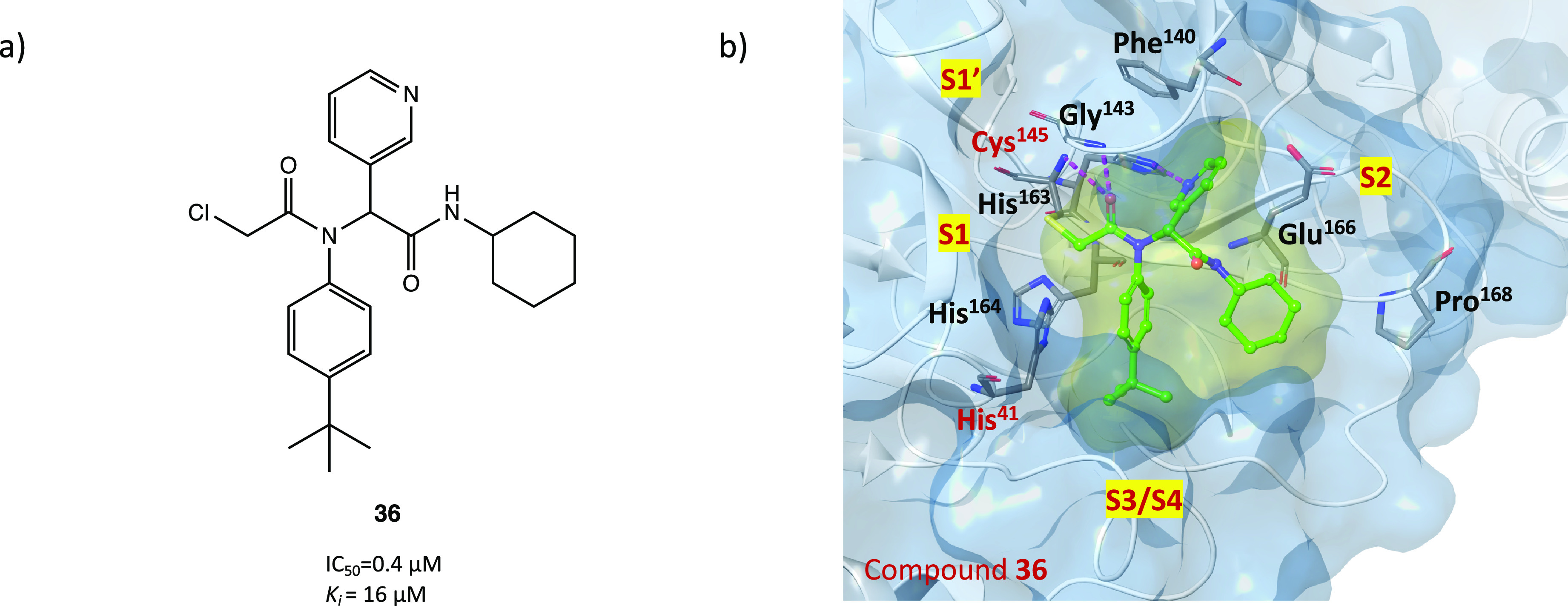
(a) Chemical structure of the α-chloroacetamide derivative **36**, developed as a SARS-CoV-2 M^PRO^ covalent inhibitor. (b) X-ray crystal structure of the covalent complex between derivative **36** and SARS-CoV-2 M^PRO^ (PDB code 7MLF).^73^

In a comprehensive in silico and in vitro screening of newly designed covalent inhibitors with different electrophilic warheads, the α-chloroacetamide derivative **36** (Figure 33a) proved to be one of the most promising lead compounds and showed a significant inhibitory effect on SARS-CoV-2 M^PRO^ (IC_50_ = 0.4 μM and *k*_i_ = 16 μM) in the biochemical assay. SAR analysis highlighted that the α-chloro substitution resulted in more effective derivatives than the α-fluoro substitution due to the higher electrophilicity of C–Cl, *tert*-butyl had a positive effect on the activity, and replacement of the heterocyclic pyridine moiety with a carbocyclic one resulted in nearly inactive compounds. Moreover, kinetic studies and the analysis of the cocrystal structure of SARS-CoV-2 M^PRO^ in complex with **36** (PDB code 7MLF) confirmed the binding mode of the proposed lead compound (Figure 33b): the cysteinyl −SH displaces the Cl through the covalent mechanism illustrated in Figure 32; the pyridine is inserted into the S2 pocket and forms multiple H bonds; the *tert*-butylphenyl is enclosed into the S4 region.^73^

**Figure 34:**
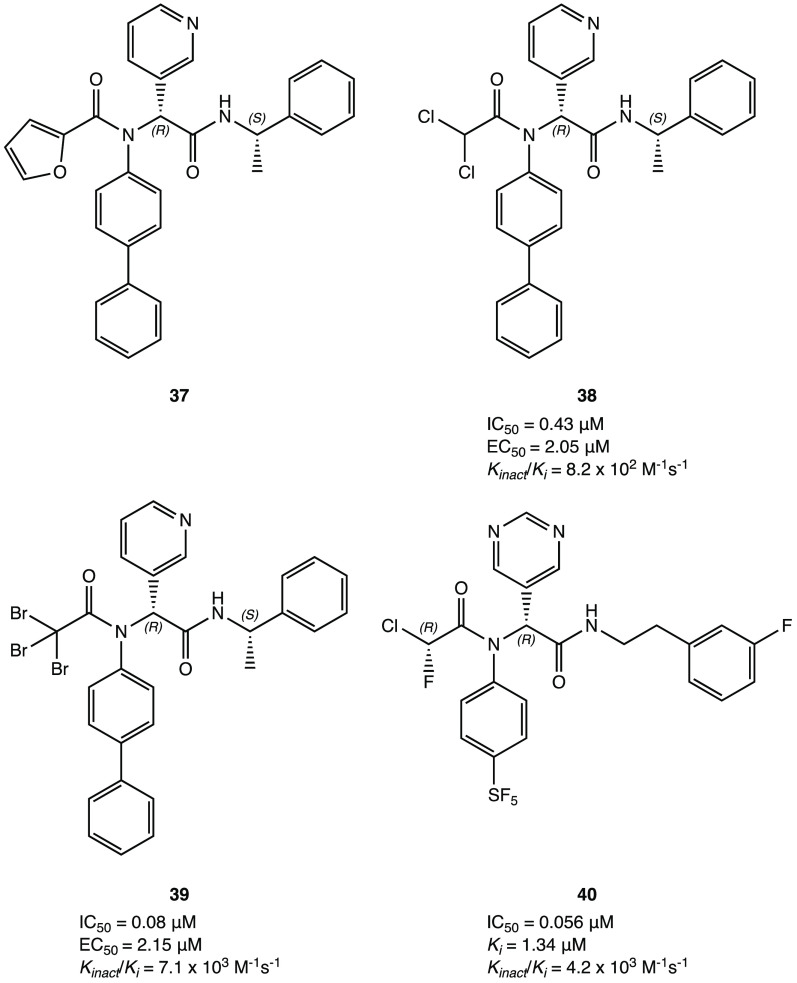
Chemical structures of the dichloroacetamide, tribromoacetamide, and chloro-fluoro-acetamide derivatives **38** and **40** developed as SARS-CoV-2 M^PRO^ covalent inhibitors.^81^

Similarly, starting from the noncovalent inhibitor **37** (Figure 34), a new series of selective SARS-CoV-2 M^PRO^ peptidomimetic covalent inhibitors was developed. The new compounds were characterized by the replacement of the furyl ring with dichloroacetamide, dibromoacetamide, tribromoacetamide, 2-bromo-2,2-dichloroacetamide, or 2-chloro-2,2-dibromoacetamide as warheads. Among all of them, derivatives **38** and **39**, shown in Figure 34, were the most promising lead structures with potent and selective M^PRO^ inhibition (IC_50_ of 0.43 and 0.08 μM, respectively) and outstanding efficacy in the cellular model of Caco2-hACE2 viral infection (EC_50_ of 2.05 and 2.15 μM, respectively). The X-ray crystal structure of the complex SARS-CoV-2 M^PRO^**38** (PDB code 7RN1) confirmed the capability of the designed compound to covalently bind to the target protein; furthermore, the absolute *R* configuration of the pyridyl P1 portion appeared essential for the deep insertion into the S1 pocket.^81^

Using these derivatives as lead compounds, a new series of SARS-CoV-2 M^PRO^ inhibitors was proposed with an interesting α-chloro-fluoroacetamide (CFA) as a warhead. Thanks to its steric and electronic features, this moiety appeared to be intrinsically selective toward cysteinyl residues and to form a pseudoreversible adduct, which could reduce the off-target labeling frequently observed for covalent inhibitors. The derivative **40** (Figure 34), with a phenyl-pentafluorosulfanyl group as P2 and a pyrimidinyl portion at P1, was the most interesting compound of the series. Remarkably, biological inhibition assays showed that the stereochemistry of the two asymmetric carbons plays a crucial role: of the four possible stereoisomers, (*R,R*)-*cis*-**40** was the only one with strong inhibitory activity against SARS-CoV-2 M^PRO^, with an outstanding IC_50_ of 0.056 μM and a remarkable *K*_inact_/*k*_i_ value of 4.2 × 10^3^ M^–1^ s^–1^. In silico simulation ascribed the higher activity of this stereoisomer to its more stable and reactive conformation within the active site.^82^

**Figure 35:**
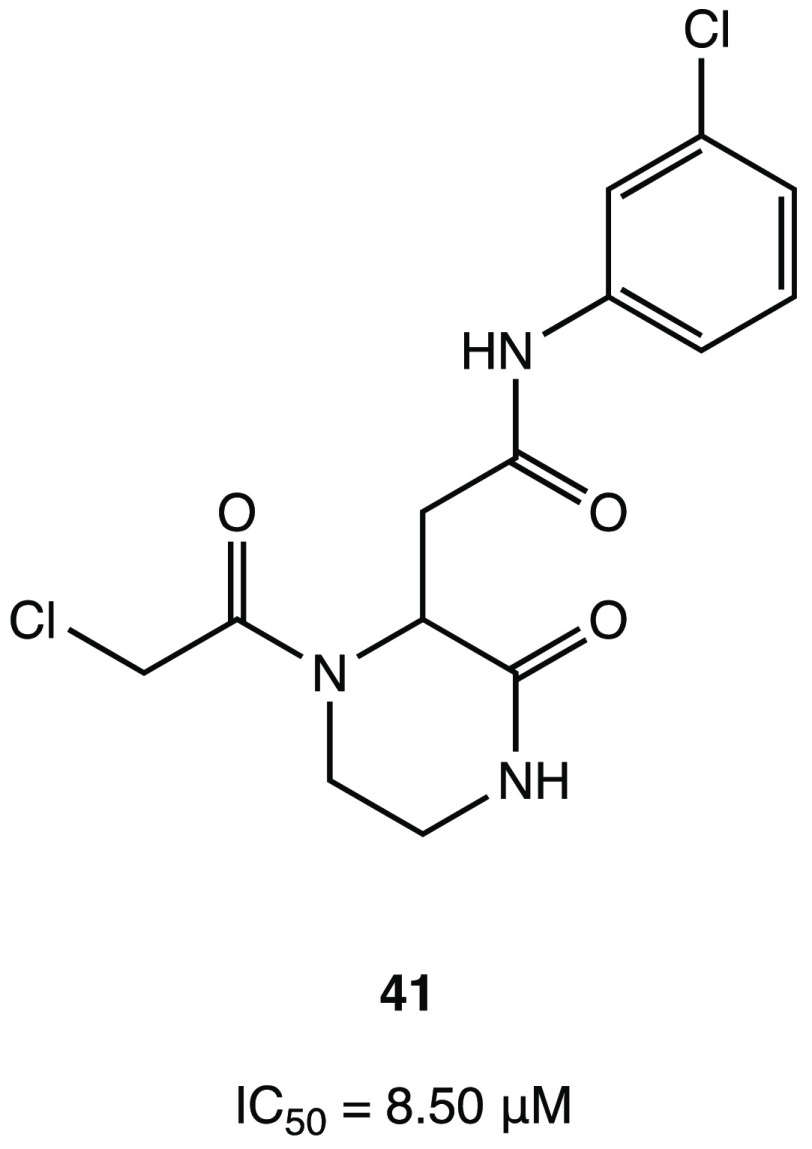
Chemical structure of α-chloroacetamide SARS-CoV-2 M^PRO^ inhibitor **41**.^83^

To discover new covalent inhibitors of SARS-CoV-2 M^PRO^, a virtual in silico screening of a set of commercial compounds without a peptidomimetic skeleton was performed. The computational protocol was based on a hierarchical workflow involving a first phase of filtering and large-scale noncovalent docking followed by the covalent docking of the best-ranked compounds. The most promising compounds were selected for in vitro enzyme inhibition assay, and among all of them, compound **41** (Figure 35) was the most active, with an IC_50_ of 8.50 μM against SARS-CoV-2 M^PRO^. Given the interesting and innovative nonpeptidomimetic core, the crystal structure of the ligand–protein complex was resolved to support the exploration of more potent analogues (PDB code 7VVT). In addition to the covalent bond formed between the cysteine and the chloroacetamide warhead, the other important noncovalent binding interactions involved the amino acid residues in the S2 site (His^41^ and Gln^189^) and the deeply inserted *m*-chlorophenyl moiety.^83^

**Figure 36:**
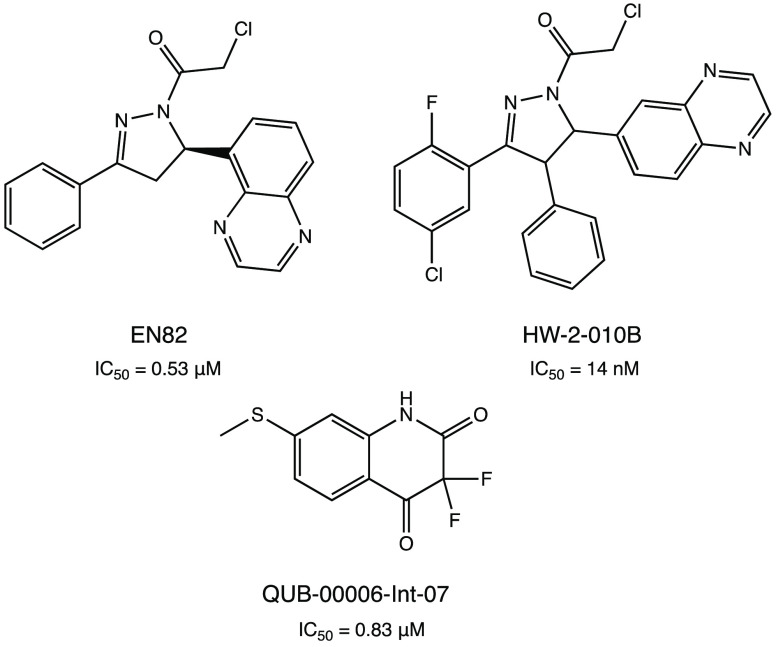
Chemical structures of pyrazoline-based compounds (*R*)-EN82, HW-2-010B, and QUB-00006-Int-07 as SARS-CoV-2 M^PRO^ inhibitors.^84,85^

By means of activity-based protein profiling (ABPP) screening, several pyrazoline-based SARS-CoV-2 M^PRO^ inhibitors with an α-chloroacetamide as a reactive group were identified. The crystal structure analysis of (*R*)-EN82 (IC_50_ value of 0.53 μM) (Figure 36) highlighted the discovery of a small protein pocket with the accommodation of the 4-phenyl substituent. Extensive SAR analyses on C-4-substituted compounds led to the isolation of the optimized trisubstituted cis derivative HW-2-010B (IC_50_ against the target protein in the nanomolar range, 14 nM) (Figure 36).^84^

The application of advanced in silico techniques, such as high-resolution all-atom molecular dynamics simulations and absolute binding free energy calculations applied on SARS-CoV-2 M^PRO^, enabled the design of the unusual cyclic α,α-difluoro-amide QUB-00006-Int-07 as a covalent inhibitor, which showed an IC_50_ of 0.83 μM (Figure 36).^85^

### 2.3 Nitrile Warhead

**Figure 37:**
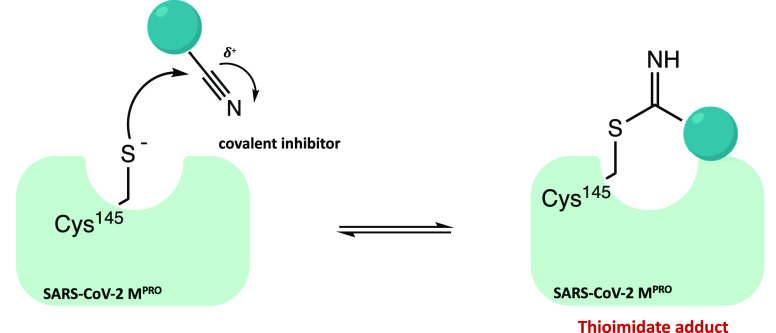
General mechanism of SARS-CoV-2 M^PRO^ inhibition with the nitrile warhead.

Interestingly, the nitrile group has also been proposed as a remarkable warhead for the development of SARS-CoV-2 M^PRO^ covalent inhibitors. Due to the difference in the electronegativity with the nitrogen atom, the carbon of nitrile is susceptible to nucleophilic addition by the catalytic cysteine of the protease with subsequent formation of the reversible thioimidate covalent adduct, as shown in Figure 37.

**Figure 38:**
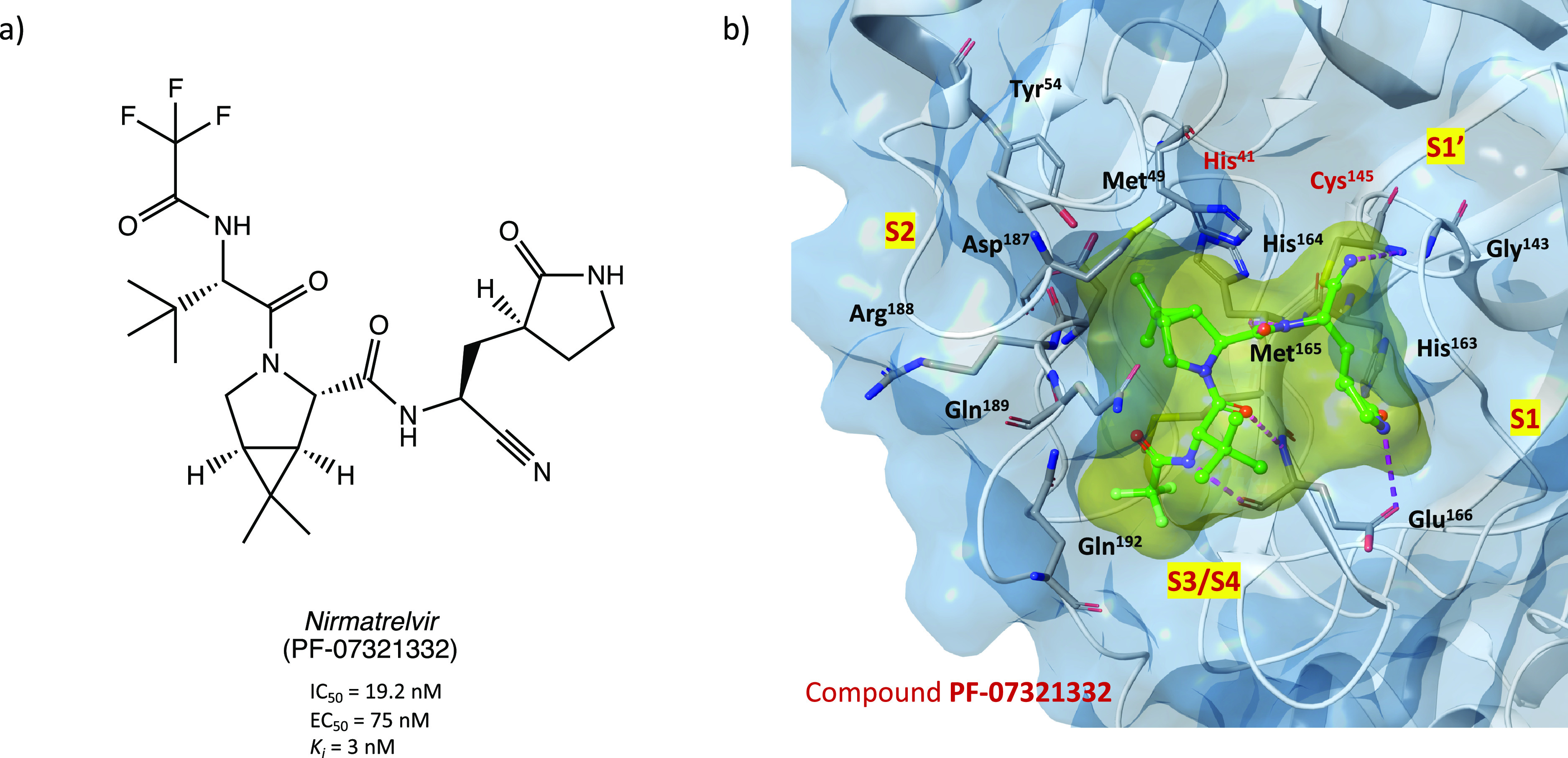
(a) Chemical structure of the orally bioavailable compound PF-07321332. (b) X-ray crystal structure of PF-07321332 in complex with SARS-CoV-2 M^PRO^ (PDB code 7VH8).^87^

In this light, Pfizer has undertaken a comprehensive drug discovery campaign to develop new SARS-CoV-2 M^PRO^ inhibitors for oral administration. Starting with the previously developed carbonyl compound PF-00835231 (Figure 11), which exhibited potent M^PRO^ inhibition and anti-SARS-CoV-2 activity but no intestinal absorption, Pfizer researchers developed the first orally bioavailable anti-SARS-CoV-2 compound PF-07321332 (nirmatrelvir) (Figure 38a) with a nitrile group as the reactive warhead. In preliminary biological assays, nirmatrelvir showed potent SARS-CoV-2 M^PRO^ inhibitory activity (*K*_i_ = 3 nM and IC_50_ = 19.2 nM) and anti-SARS-CoV-2 activity in cell-based assays (EC_50_ = 75 nM). Its less peptidomimetic structure (fewer H-bond donors and lower polarity compared to the parent compound) and the insertion of a trifluoroacetamide moiety guaranteed excellent intestinal barrier permeation and excellent oral bioavailability compared to the first in vivo evaluations.^21,86^

Thanks to its remarkable results obtained in the preclinical evaluation, it is currently under phase 3 of clinical trials in combination with ritonavir (PAXLOVID, see ClinicalTrials.gov Identifier NCT04960202), an already approved anti-HIV agent inserted in the formulation as a pharmacokinetic enhancer (it increases the systemic exposure and the half-life of nirmatrelvir thanks to its inhibitory activity on the cytochrome metabolizing enzymes). The good results already obtained prompted the European Medicines Agency (EMA) and the corresponding authorities of United States and United Kingdom to grant a conditional marketing authorization for the treatment of COVID-19.^23,88,89^

From a medicinal chemistry point of view, the crystal structure of SARS-CoV-2 M^PRO^ in complex with PF-07321332 (PDB codes 7VH8 and 7VLQ) has been resolved to clarify the mechanism of action of this compound. As shown in Figure 38b, the nitrile warhead is able to form a reversible covalent thioimidate adduct with the sulfur atom of the Cys^145^ at S1′; furthermore, the formed imine nitrogen enhances the reversible adduct by interacting with the Gly^143^ and Cys^145^ residues of the oxyanion hole. The usual γ-lactamic ring fits into the S1 pocket and interacts with His^163^ and Glu^166^, while the dimethyl-bicycloproline group is collocated into the hydrophobic S2 pocket and is surrounded by the side chains of His^41^, Met^49^, Tyr^54^, Met^165^, and Gln^189^, resulting in extensive van der Waals interactions. The trifluoromethyl group, instead, is important to anchor the inhibitor at the S4 subpocket by forming a stabilizing contact with Gln^192^ and two ordered small molecules positioned in this site.^87,90^

Furthermore, given the importance of PF-07321332 as the first SARS-CoV-2 M^PRO^ inhibitor approved for clinical use, a lot of advanced computational studies, such as steered molecular dynamics and classical-hybrid QM/MM simulations, have been conducted with the aim to clarify the mechanism of binding/inhibition and thus to guide the design of new analogues. It has been demonstrated that P1 (γ-lactamic ring) and the P2 (dimethyl-cyclopropylproline group) are essential in the ligand-binding process, contributing positively to the total binding free energy (substitution with other groups determined a drastic reduction of binding affinity). On the other hand, it has been evidenced that the P3 and P4 groups (isobutyl and trifluoromethyl) made a favorable but small contribution to the binding free energy, suggesting the possibility of modification on these sites to increase the binding strength.^91,92^

In addition, given the widespread distribution of SARS-CoV-2 variants of concern (beta, delta, and the rapidly spreading omicron variant), several studies are currently being conducted to investigate the efficacy of PF-07321332, providing very encouraging results. Indeed, nirmatrelvir showed capability to inhibit the most prevalent M^PRO^ variants expressed by the most diffused SARS-CoV-2 mutant lineages in vitro^93^ and capability to potently block the infection of beta, delta, and omicron SARS-CoV-2 variants in both in vitro and in vivo animal models.^94−98^

**Figure 39:**
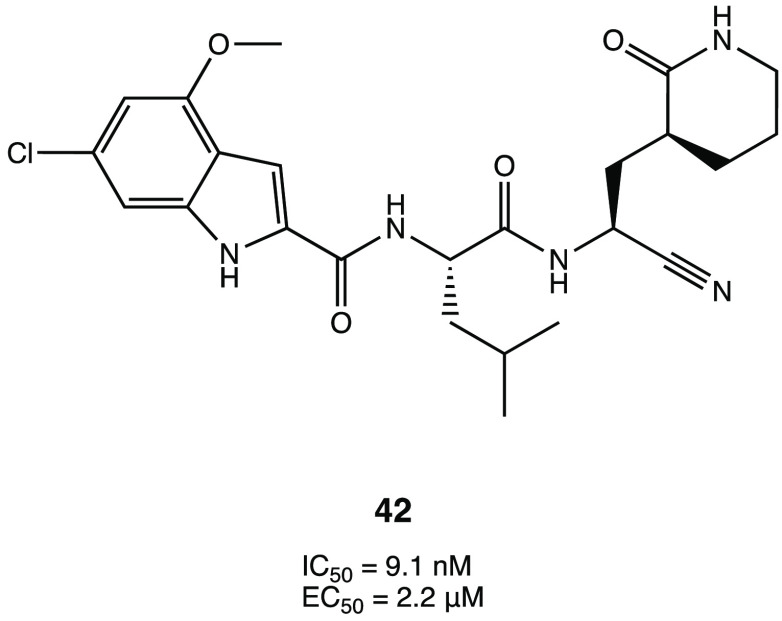
Chemical structure of the nitrile derivative **42**.^99^

Starting from the carbonyl derivatives GC-376 or PF-07304814, several analogues were designed with a nitrile warhead to explore the impact of this one on the inhibition activity. Among all of them, compound **42** (Figure 39) was the most active in the inhibition assay with an IC_50_ of 9.1 nM against the target protein and an EC_50_ of 2.2 μM in a plaque reduction assay conducted in Vero E6-infected cells. In general, by analyzing the results for the whole series, it appears that substitution of the carbonyl warhead with a nitrile one positively affects the activity, leading to more active and selective compounds.^99^

### 2.4 Ester Warhead

**Figure 40:**
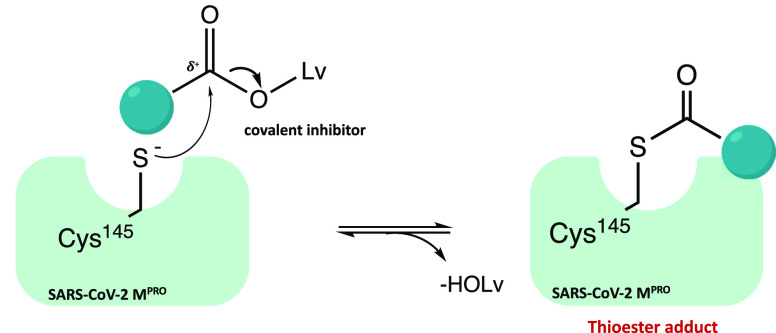
Mechanism of acylation of SARS-CoV-2 M^PRO^ mediated by ester derivatives.

Figure 40 shows the general mechanism of action of the class of inhibitor molecules with an ester warhead, which involves a nucleophilic attack of the Cys^145^ on the electrophilic carbonyl of the ester group followed by cleavage of the alkoxy group (−OLv) and irreversible acylation of the enzyme.

**Figure 41:**
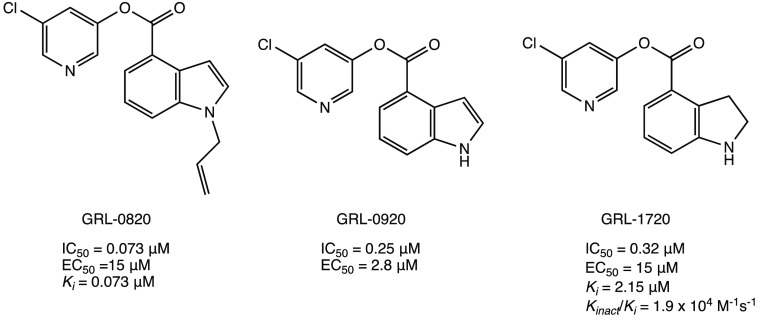
Chemical structures of chloropyridinyl esters GRL-0820, GRL-0920, and GRL-1720 repurposed as SARS-CoV-2 M^PRO^ inhibitors.^52,100,101^

The class of indole/indoline chloropyridinyl esters is one of the most frequently described in the literature as SARS CoV-2 M^PRO^ inhibitors.^52,100−102^ In particular, three indole/indoline-chloropyridinyl-ester derivatives (GRL-0820, GRL-0920, and GRL-1720 in Figure 41), which have already been evaluated against SARS-CoV-1, were investigated as promising lead compounds for the design of new SARS-CoV-2 M^PRO^ inhibitors. All compounds showed potent inhibitory activity against SARS-CoV-2 M^PRO^ (IC_50_ values of 0.073, 0.25, and 0.32 μM, respectively) and anti-SARS-CoV-2 activity in Vero E6-infected cells with EC_50_ values of 15, 2.8, and 15 μM, respectively.^52,100,101^

In silico docking analyses and kinetic studies proved that all three derivatives are able to covalently bind the catalytic Cys^145^ through an acyl substitution (as described in the general mechanism of action in Figure 40).^100,101^

**Figure 42:**
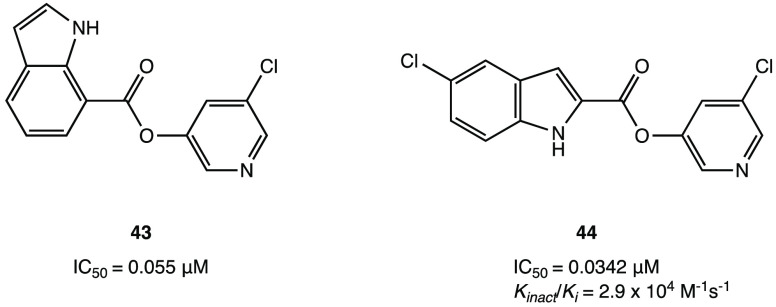
Chemical structures of indole derivatives **43** and **44**.^102^

Considering that the chloro-substituted pyridinyl group is a common pharmacophoric moiety, a new series of indole esters was studied (Figure 42). Compounds **43** and **44** proved to be the most potent inhibitors with IC_50_ values of 0.055 and 0.0342 μM, respectively. Further biological assays showed that both compounds could inhibit M^PRO^ in HEK and A549 human lung epithelial cell lysates.^102^

**Figure 43:**
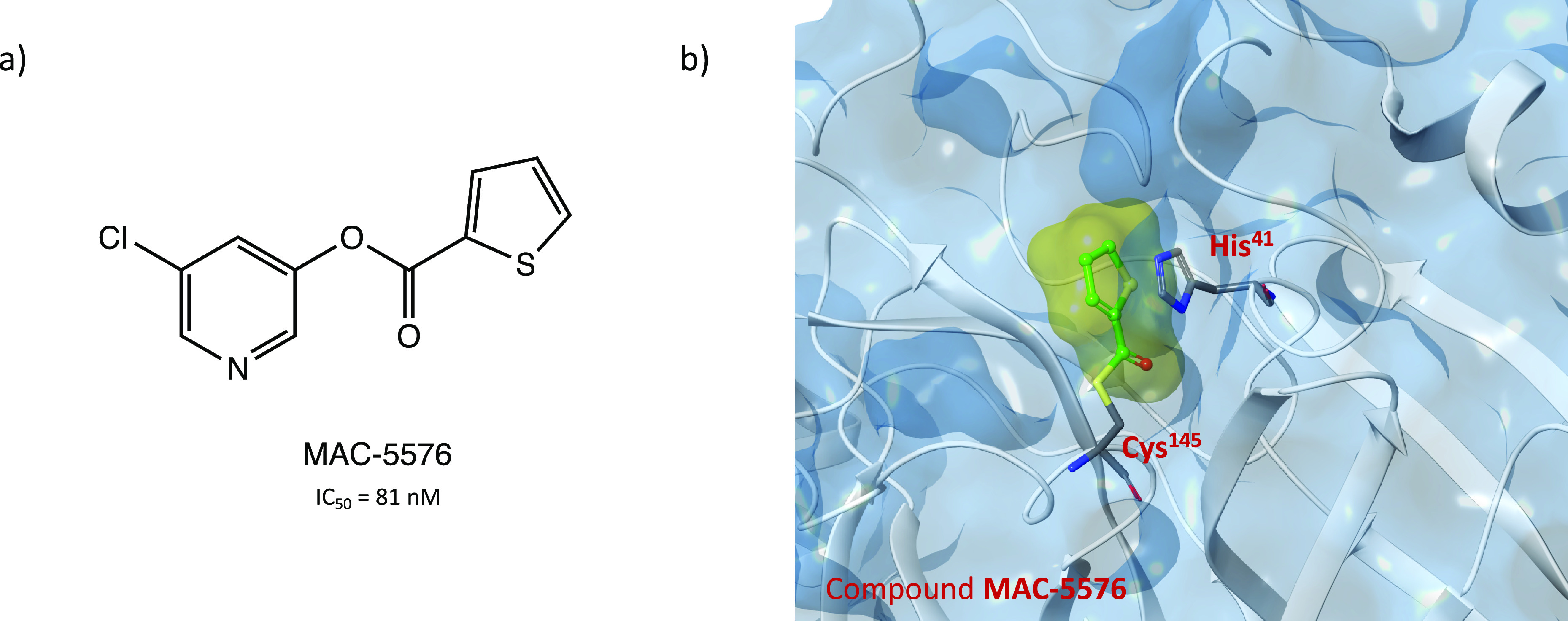
(a) Chemical structure of MAC-5576, a chloropyridinyl ester SARS-CoV-2 M^PRO^ inhibitor. (b) Crystal structure of the covalent complex SARS-CoV-2 M^PRO^ with MAC-5576 (PDB code 7JT0).^69^

Interestingly, through an extensive in vitro screening of previously developed SARS-CoV-1 M^PRO^ inhibitors, the chloropyridinyl ester MAC-5576 (Figure 43a) was selected as new covalent inhibitor of SARS-CoV-2 M^PRO^, exhibiting an interesting IC_50_ value of 81 nM. However, in the cytopathic reduction assay performed in Vero E6 cells, it did not show the desired inhibition of viral infection. Nevertheless, the X-ray crystal structure of MAC-5576 in complex with the target protein has been resolved to highlight the importance of this new nonpeptidomimetic scaffold for the design of new covalent inhibitors: as shown in Figure 43b (PDB code 7JT0), the compound is able to acylate the protease.^69^

**Figure 44:**
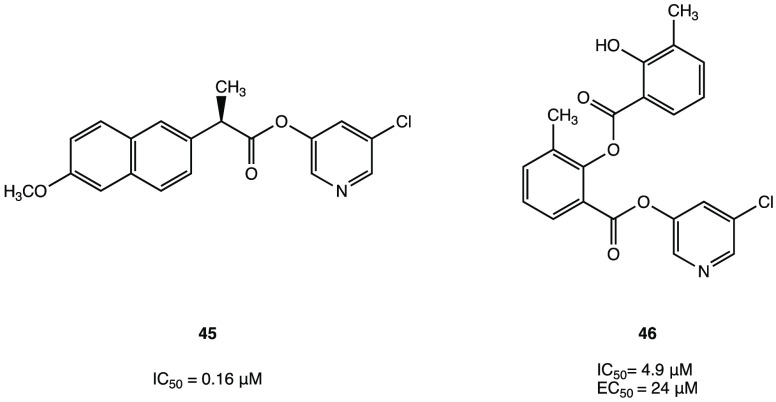
Chemical structures of 5-chloropyridinyl ester derivatives of nonsteroidal anti-inflammatory agents **45** and **46**.^103^

As part of the SARS-CoV-2 M^PRO^ inhibitors with an ester warhead, a series of new 5-chloropyridinyl esters of nonsteroidal anti-inflammatory drugs (NSAIDs, such as salicylic acid, ibuprofen, naproxen, and indomethacin) is of particular interest. Among all of them, (*R*)-naproxen derivative **45** (Figure 44) with an IC_50_ value of 0.16 μM was the most interesting compound. In addition, compound **46** (Figure 44), which proved less efficient in the inhibition assay (IC_50_ = 4.9 μM), exhibited a potent antiviral activity in cellular assays (EC_50_ = 24 μM in Vero E6 cells). MALDI-TOF analysis was used to demonstrate the ability of the two compounds to covalently bind SARS-CoV-2 M^PRO^, leading to the irreversible acylation of the enzyme.^103^

### 2.5 Selenium/Sulfur as Electrophilic Warheads: The Case of Bselen/Ebsulfur and Analogues

The possibility of using drugs approved in therapy (well-known substances with proved efficacy and safety in humans) in the treatment of COVID-19 allows one to reduce the time and costs associated with the development of a new molecule.

**Figure 45:**
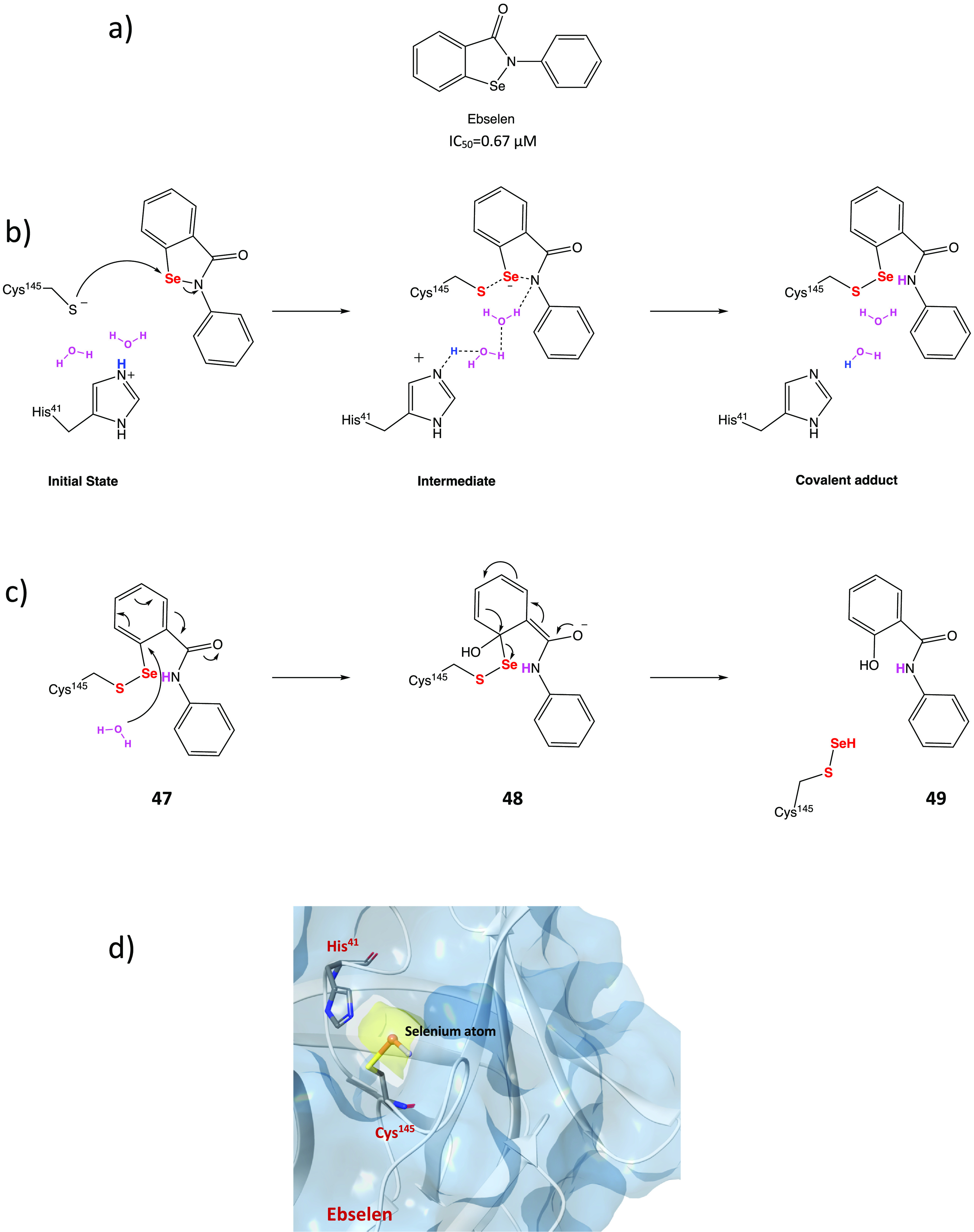
(a) Chemical structure of ebselen. (b) Proposed mechanism of action of ebselen with SARS-CoV-2 M^PRO^. (c) Proposed mechanism for selenylation of the catalytic site of SARS-CoV-2 M^PRO^. (d) Crystal structure of the selenylated cysteine^145^ in the SARS-CoV-2 M^PRO^ binding site (PDB code 7BAK).^107−109^

Ebselen (2-phenyl-1,2-benzisoselenazol-3(2*H*)-one, in Figure 45a), a heterocyclic structure with a selenium atom that has been studied as an antioxidant/anti-inflammatory agent,^104,105^ was one of the most interesting examples of repurposing of investigational drugs against SARS-CoV-2.

Analysis of in silico and in vitro data demonstrated the strong antiviral activity of ebselen (IC_50_ = 0.67 μM against SARS-CoV-2 M^PRO^) and the capability to covalently bind the catalytic Cys^145^ of SARS-CoV-2 M^PRO^.^15,106^

To further explore the mechanism of covalent inhibition of this compound, a combination of docking and density functional theory (DFT) protocols clarified the ebselen ability to form a covalent adduct with Cys^145^ by the formation of a selenyl sulfide bond.^107^ In Figure 45b the mechanism of action of ebselen is shown: the first step is the activation of the thiol group of Cys^145^ through deprotonation mediated by His^41^; subsequently, the activated thiolate performs the nucleophilic attack on the electrophilic selenium atom, determining the opening of the 5-membered ring and the formation of selenyl sulfide bond, responsible for the covalent inhibition of the target. The process is mediated by a molecule of water that acts as a proton transporter.^108^ A mass spectrometry study suggested an additional rearrangement of the covalent adduct (Figure 45c) consisting in the hydrolysis of the ebselen-SARS-CoV-2 M^PRO^ adduct (**47**) by a conserved molecule of water, forming an intermediate state (adduct **48**), with subsequent selenylation of the cysteine and release of a secondary phenolic product **49**. The proposed mechanism of action was confirmed by the X-ray structure shown in Figure 45d (PDB code 7BAK), where the selenium atom is covalently bonded to the cysteinyl sulfur.^109^

Moreover, ebselen was found to bind an allosteric site between the I and the II domains of M^PRO^ (which is essential for the dimerization process).^109,110^

In view of the aforementioned data, ebselen and its analogues could represent promising lead compounds for the future development of new and more effective covalent inhibitors. In this light, many efforts have been made to analyze the SAR of the substituted *N*-phenyl ring.

**Figure 46:**
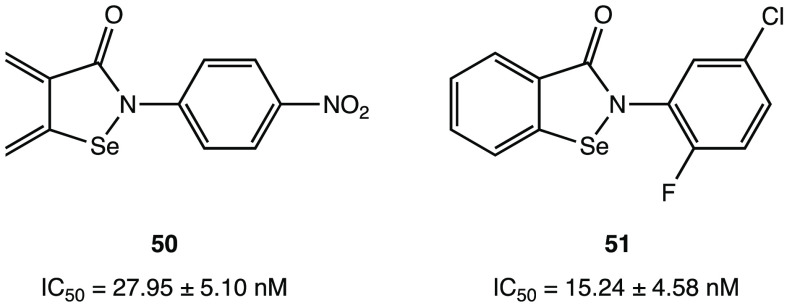
Ebselen derivatives **50** and **51**.^111^

Indeed, a collection of ebselen-related compounds has been extensively studied. Among them, **50** and **51** (4-nitro- and 5-chloro-2-fluoro derivatives, respectively; Figure 46) displayed inhibitory activity superior to that of ebselen with IC_50_ values of 27.95 ± 5.10 and 15.24 ± 4.58 nM, respectively.^111^ According to SAR analysis, the authors demonstrated that the insertion of one or two substituents on the phenyl ring can improve the inhibition activity against SARS-CoV-2 M^PRO^.^111^

Similarly, four ebselen-based compounds **52**–**55** (Table 1) exhibited greater effectiveness than ebselen both against M^PRO^ (IC_50_ in the submicromolar range) as well as in the viral cell model of SARS-CoV-2 replication.^109^

**Table 1:**
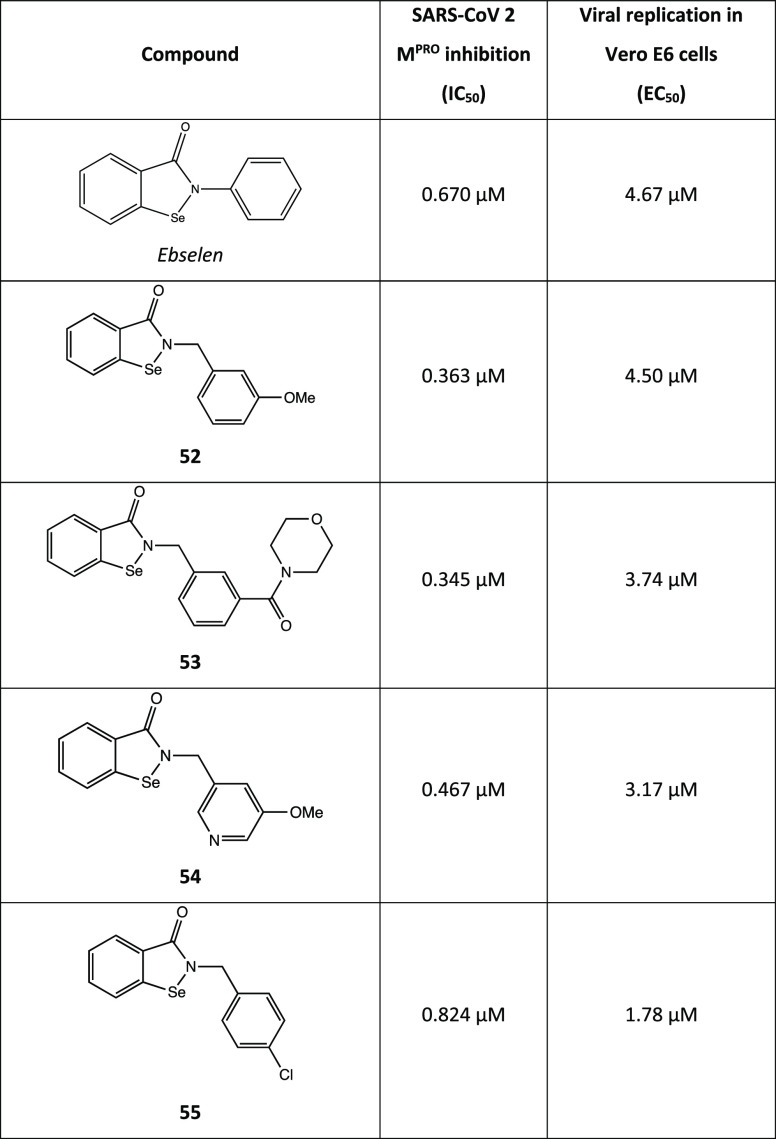
Chemical Structures, SARS-CoV-2 M^PRO^ Inhibitory Activity, and Inhibition of Viral Replication Data of Ebselen Derivatives **52–55**(109)

**Figure 47:**
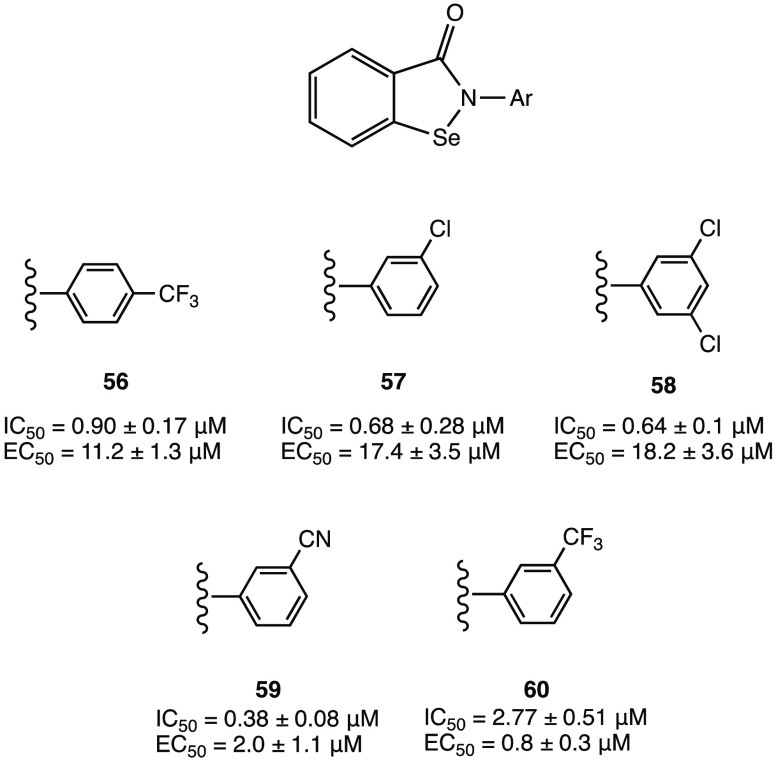
Chemical structures of phenyl-substituted ebselen derivatives **56**–**60**.^112^

To further explore the SAR for this class of compounds, a series of *N*-phenyl-substituted ebselen derivatives was synthesized. Compounds **56**–**60** (Figure 47) exhibited submicromolar IC_50_ values against SARS-CoV-2 M^PRO^ (in the range 0.38–2.77 μM), suggesting that the substitution is favorable in the meta position. As for derivative **59**, the most potent compound, the presence of a cyano group in the meta position improves the interaction network with an additional favorable hydrogen bond. Nevertheless, biological assays conducted in an in vitro cellular model of SARS-CoV-2 replication (Vero E6-infected cells) showed that compound **60**, which was the least active in the enzymatic inhibition assay, was surprisingly the most effective in blocking viral progression (EC_50_ = 0.8 μM). For this reason it was selected for further studies in lung organoids, which confirmed the lack of toxicity and its ability to block viral replication.^112^

**Figure 48:**
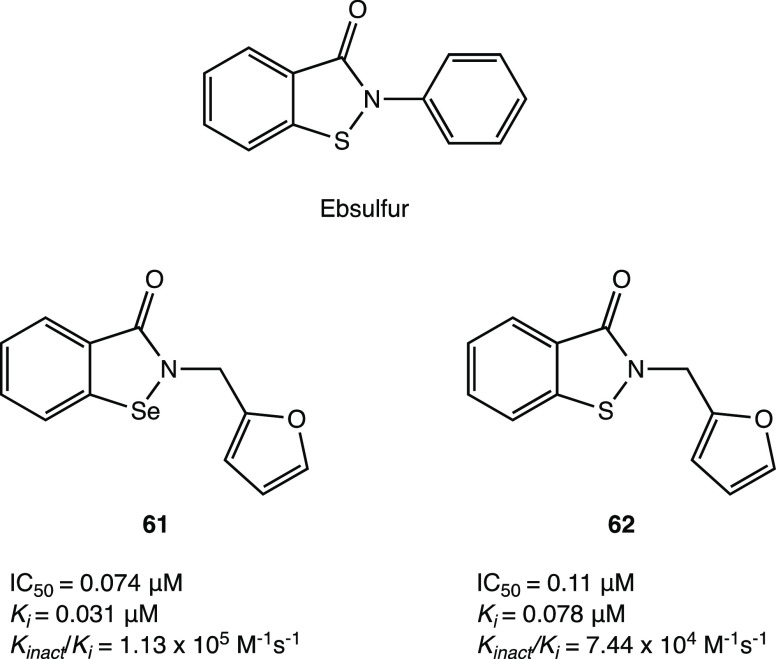
Chemical structures of ebsulfur and ebselen/ebsulfur analogues **61** and **62**.^113^

The isosteric analogue of ebselen, ebsulfur, with a sulfur atom in place of selenium (Figure 48), also attracted considerable interest as a covalent SARS-CoV-2 M^PRO^ inhibitor. Docking studies have shown that ebsulfur, in the same way as ebselen, can form a covalent S–S bond with the cysteinyl −SH of SARS-CoV-2 M^PRO^. On the basis of this assumption, numerous ebselen/ebsulfur analogues as SARS-CoV-2 M^PRO^ inhibitors were designed and biologically evaluated. Among all of them, **61** and **62** were the most interesting with IC_50_ values of 0.074 and 0.11 μM, respectively. Docking studies proved that the furan group is essential to form additional hydrophobic interactions with Met^165^, Arg^188^, Asp^187^, and Met^49^. From this study it emerged that ebsulfur as well as ebselen could be a potential lead compound for the development of novel, broad-spectrum anticoronaviral drugs.^113^

**Figure 49:**
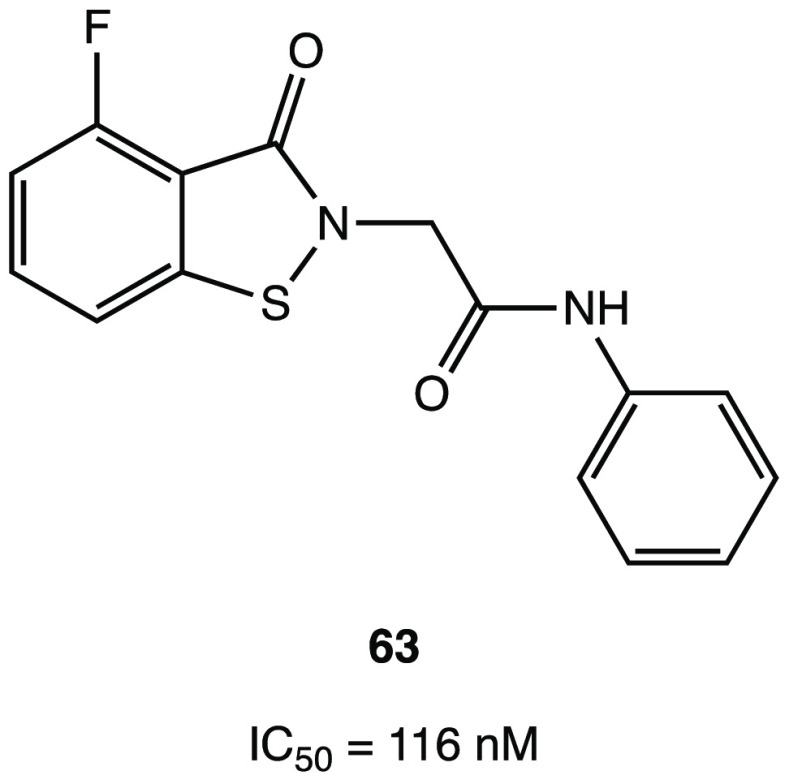
Chemical structure of ebsulfur analogue **63**.^114^

The structure–activity relationships of ebsulfur were investigated focusing on three recurring components: the phenyl ring, the linker, and the benzoisothiazolone core. In particular, compound **63** (Figure 49) was found to be the best ebsulfur analogue with covalent inhibitory activity on SARS-CoV-2 M^PRO^. The phenyl and benzoisothiazolone rings were retained in view of their importance in the interaction with the target protein; instead, introduction of an acetamide group in the linker had a positive effect on the activity, which was confirmed by the remarkable IC_50_ value of 116 nM against SARS-CoV-2 M^PRO^.^114^

**Figure 50:**
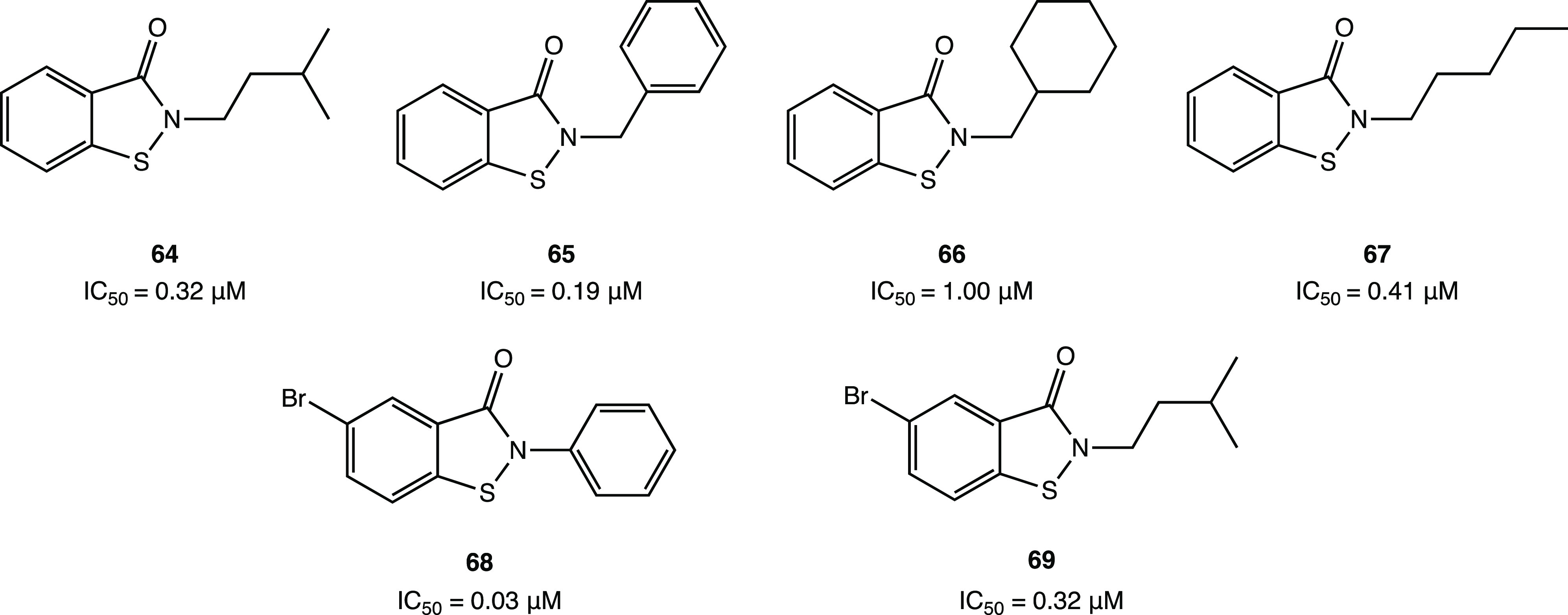
Chemical structures of the promising ebsulfur derivatives **64**–**69**.^115^

In the same vein, SAR investigation led to the identification of other notable ebsulfur analogues **64**, **65**, **66**, and **67** (Figure 50) with significant IC_50_ values of 0.32, 0.19, 1.00, and 0.41 μM, respectively, against SARS-CoV-2 M^PRO^. Further structural optimization involved the introduction of a −Br in both ebsulfur and **59** to obtain the derivatives **68** and **69** (Figure 50), which exhibited an improved inhibitory activity against SARS-CoV-2 M^PRO^ (IC_50_ = 0.03 and 0.32 μM, respectively) compared to ebsulfur (IC_50_ = 0.13 μM).^115^

## 3 Conclusions and Perspectives

SARS-CoV-2 M^PRO^ is a key cysteinyl enzyme for virus replication and consequently human infection. Considering the previous findings on SARS-CoV-2 M^PRO^-selective inhibitors, covalent inhibition represents one of the most interesting strategies in drug discovery projects. The possibility of linking the nucleophilic cysteinyl −SH to a reactive electrophilic group (warhead) may allow more effective and durable inhibition of the targeted protein.

Given the large number of studies conducted in this field, we decided in the present review to analyze the most relevant and informative examples of covalent SARS-CoV-2 M^PRO^ inhibitors. Specifically, our approach consisted of a primary classification of the studied compounds into eight classes depending on the nature of their electrophilic warheads: aldehyde, ketone, α-ketoamide, Michael acceptor, α-haloacetamide, nitrile, activated ester, and molecules containing an electrophilic selenium/sulfur atom. Particular attention was paid to the description of the mechanism of the catalytic inhibition, highlighting the main features and the reversibility/irreversibility of the covalent adduct formed by each warhead.

Within each class, SAR analyses evaluating the peptidomimetic and nonpeptidomimetic aspects were presented. Among these, the peptidomimetic compounds represent the largest class described in the literature to date due to their intrinsic ability to mimic the natural substrates of SARS-CoV-2 M^PRO^. The wide number of SARS-CoV-2 M^PRO^ crystal structures in complex with the inhibitors (some of which have been shown in detail as examples) allowed us to highlight the most recurrent structural features and interactions with the target protein.

Table 2 lists the SARS-CoV-2 M^PRO^ inhibitory activity and the main structural moieties (P fragments) of the most interesting compounds. Furthermore, special attention was paid to the stereochemistry of the chiral carbons, which is crucial for the arrangement of the ligands to achieve a better fit to the SARS-CoV-2 M^PRO^ binding pocket. Following the Schechter–Berger notation, the essential structural moieties (P1′ = electrophilic warhead, P1, P2, P3) of each compound were correlated with the SARS-CoV-2 M^PRO^ subregions (S1′, S1, S2, S3/S4). All of the analyzed inhibitors are able to form a covalent adduct with the Cys^145^ catalytic residue in the S1′ subregion, and several additional H bonds stabilize the ligand–protein complex with the Cys^145^ and Gly^143^ backbones and/or the His^41^ side chain.

**Table 2:**
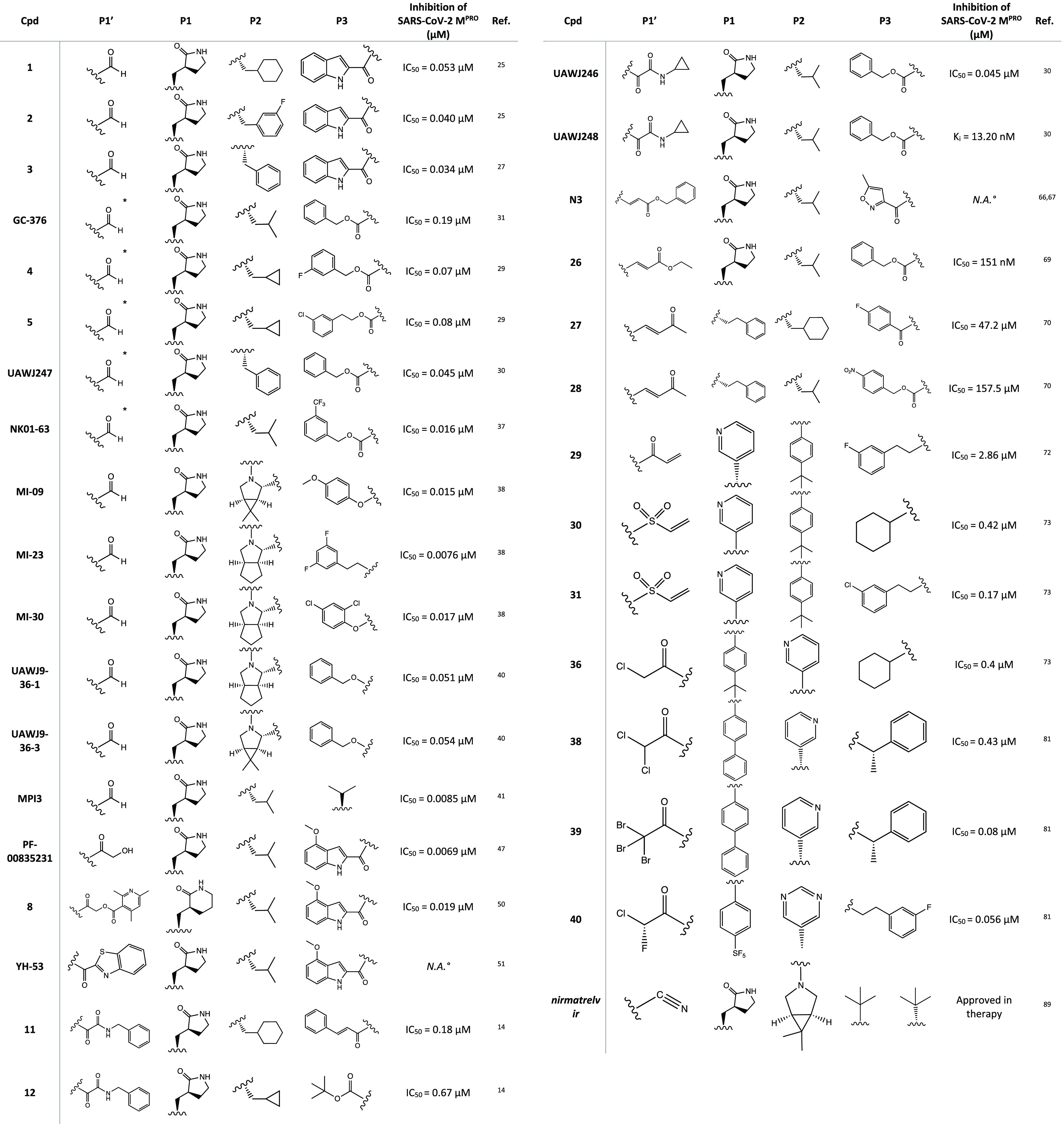
Analysis of Structural Fragments and Inhibition Data of the Most Active Covalent SARS-CoV-2 M^PRO^ Inhibitors by Following the Schechter–Berger Notation

a Warhead masked as prodrug.

b N.A.: Biological data not available.

Regarding the adjacent S1 site, which accepts the Gln residue of the native substrate, the most recurrent fragment in P1 is the five-membered γ-lactamic ring. Indeed, thanks to its capability to mimic the natural amino acid glutamine, it is capable of penetrating deeply into the pocket and forming a stabilizing interaction with key residues (Phe^140^, His^163^, His^164^, Glu^166^). The stereochemistry of the P1 five-membered γ-lactamic moiety deserves special attention: the two asymmetric carbons have generally the same configurations (*S, S*). By observing the X-ray structures, it is evident that this orientation within the S1 pocket guarantees an optimal fit and conformation, to be maintained in the design of new inhibitors.

The SARS-CoV-2 M^PRO^ S2 cleft, comprising mainly hydrophobic amino acids (Met^49^, Tyr^54^, Met^165^, Pro^168^, Val^186^), appears to be very flexible, allowing it to bind both small and bulky aromatic/alkylic portions (P2), such as cyclohexyl,^14,25,70^ cyclopropyl,^14,29^ isoleucine,^30,31,37,41,47,50,51,69,70^ phenyl,^25,27,30,72,73,81^ and bicycloalkyl^38,40,87^ moieties (such as cyclopentylproline and dimethylcyclopropylproline in the analogs of boceprevir and telaprevir). As in the case of the P1 fragment, the *S* configuration at the asymmetric carbon is pivotal to allow a more favorable orientation within the pocket.

To interact with the hydrophobic pocket of the S3/S4 subregion, most of the covalent SARS-CoV-2 M^PRO^ inhibitors have P3 aromatic moieties, such as indole,^116^ substituted phenyl (especially with halogens (fluoride,^29,38,70,72,81^ chloride,^29,38,73^ or trifluoromethyl groups^37^)), and an aliphatic portion, such as a *tert*-butyloxycarbonyl group.^14^ The most representative interactions occur with the amino acids Gln^189^, Leu^167^, Pro^168^, Gly^251^, and Asp^187^ and with the conserved water molecules on the surface of the S4 site.

Moreover, in Table 2 the biological data of the reviewed compounds are reported. Most of the SARS-CoV-2 M^PRO^ inhibitors show IC_50_ values in the low micromolar/nanomolar range against M^PRO^ and interesting antiviral activity in the cellular infection model. Among all GC-376 (with an aldehydic warhead), PF-00835231 and the corresponding phosphate prodrug PF-07304814 (ketone warheads) have already reached clinical in vivo studies and/or trials.

In particular, nirmatrelvir (PF-07321332), the first covalent SARS-CoV-2 M^PRO^ inhibitor currently approved in the therapy for the COVID-19 treatment with a conditional marketing authorization, was obtained thanks to a lead optimization study starting from the carbonyl compound PF-00835231. Nirmatrelvir is an orally bioavailable peptidomimetic derivative with a nitrile group as an electrophilic warhead P1′, a γ-lactamic ring in P1, a dimethylbicycloproline group in P2, and a trifluoromethyl group in P3, which plays a leading role in stabilizing the covalent thioimidate adduct.

Regarding the nonpeptidomimetic covalent SARS-CoV-2 M^PRO^ inhibitors, they are less explored in the literature but are beginning to emerge, such as the ester, natural, and ebselen/ebsulfur derivatives reported in this review.

Among the natural covalent inhibitors, the quinonoid/flavonoid systems have been extensively studied in silico/in vitro. In particular, myricetin, which exhibits high inhibitory activity (with an IC_50_ of 0.2–0.6 μM against M^PRO^ and an EC_50_ value of 8 μM in infected Vero E6 cells) and the ability to alleviate the overall inflammation in the in vivo mice lung injury model acts as a Michael acceptor through its hidden electrophilic pyrogallic moiety. The α,β-unsaturated carbonyl group is released following the pyrogallol oxidation step. The proposed irreversible mechanism of inhibition is confirmed by the solved X-ray crystal structure in complex with SARS-CoV-2 M^PRO^, which shows the covalent bond between the C-6′ of myricetin and the catalytic Cys^145^.

Another interesting class of nonpeptidomimetic derivatives is the ester SARS-CoV-2 M^PRO^ inhibitors, whose mechanism of action involves irreversible acylation of the enzyme following nucleophilic attack of Cys^145^ on the electrophilic carbonyl of the ester.

The presence of the chloro-substituted pyridinyl group is observed in all of the analyzed compounds and represents an essential pharmacophoric moiety; furthermore, maintaining the chloro-pyridinyl portion, the class of indole esters has been extensively examined in the literature as SARS CoV-2 M^PRO^ inhibitors, Table 3.

**Table 3:**
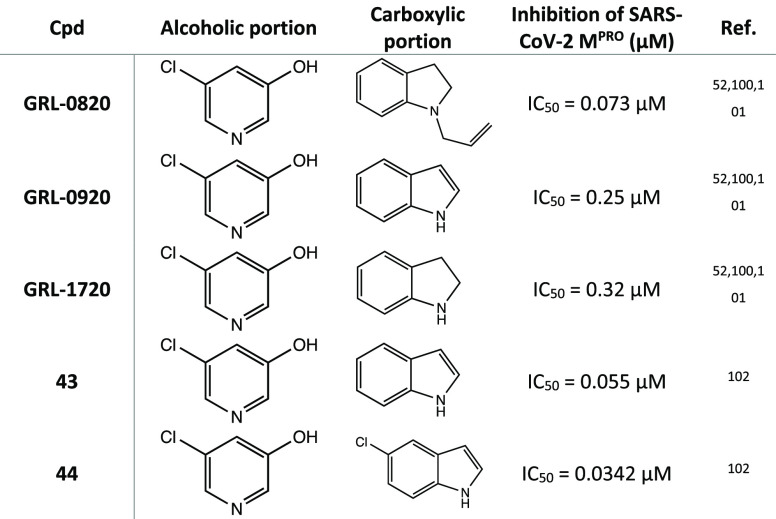
Analysis of Structural Fragments and Inhibition Data of the Most Active Ester SARS-CoV-2 M^PRO^ Inhibitors

Finally, ebselen and ebsulfur, heterocyclic structures containing a selenium/sulfur atom, represent one of the most interesting examples of repurposing of investigational compounds against SARS-CoV-2 with potent antiviral activity and capability to covalently bind the catalytic Cys^145^ of SARS-CoV-2 M^PRO^.

In view of these considerations, ebselen, ebsulfur, and their analogues could represent promising lead compounds for the future development of new and more effective covalent inhibitors.

In particular, *N*-phenyl ring substitutions improve the inhibition activity against SARS-CoV-2 M^PRO^ and lead to compounds with submicromolar IC_50_ values against SARS-COV-2 M^PRO^ (in the range 0.38–2.77 μM).

As highlighted in the previous sections, we reported some examples of ligand/structure-based, hybrid or nonhybrid, virtual screenings, and new computational approaches that led to biologically interesting compounds. In some cases, the integration of noncovalent and covalent docking protocols led to compounds with optimal interaction with both the catalytic cysteine and the other amino acids residues in the clefts of the binding site.^117,118^ Furthermore, QSAR and/or pharmacophore modeling approaches combined with molecular docking, MD simulations, and free binding energy MM/PBSA enabled the development of new derivatives with promising covalent SARS-CoV-2 M^PRO^ inhibitory activity.^26,67,68,70−72,83^

In addition to the common techniques, quantum mechanics/molecular mechanics (QM/MM) calculations, although less common, are gradually becoming reliable computational approaches in the design of covalent inhibitors.^24^ These techniques, capable of simulating the chemical reactions occurring in enzymes at the atomistic/molecular level, are suitable for elucidating the mechanism of action, kinetics, and thermodynamics of covalent modification in a target protein.^119^ In particular, their application helped to clarify the covalent inhibition mechanism of SARS-COV-2 M^PRO^ and the proton transfer process during the nucleophilic attack of the catalytic dyad.^120−122^

Furthermore, innovative techniques have been developed, such as the combined protocol of Advanced Deep Q-learning Network and Fragment-Based Drug Design (ADQN-FBDD), artificial intelligence (AI) with structure-based drug design (SBDD),^64^ and the new computational pipeline covalentizer,^72^ which aims to accelerate the generation of potential lead compounds and the identification of irreversible inhibitors.

In light of these considerations, we believe that an optimal integration and optimization of all of these available in silico methods could represent a crucial challenge to increasing the hit/lead compound identification rate in the current pandemic.^118,123^

### 3.1 Kinetic Covalent Inhibition

*K*_i_ values describe the exact binding affinity between the inhibitor and enzyme and provide information about the formation of the covalent complex (E–I). Determination of *K*_i_ and/or *K*_inact_/*K*_i_ values, which reflect the binding affinity of drugs, is a prerequisite for the prediction and evaluation of drug–enzyme interactions. An important factor to consider in the development of a therapeutic protease inhibitor is the reversibility of the binding E–I complex. Irreversible protease inhibitors can achieve long-lasting effects by permanently blocking proteases and covalently modified proteins.

Table 4 lists the enzyme inhibition data (*K*_i_ or/and *K*_inact_/*K*_i_) of covalent SARS-CoV-2 M^PRO^ inhibitors reported in the literature and their SARS-CoV-2 M^PRO^ inhibitory and antiviral activity (IC_50_ (μM), EC_50_ (μM) and ratio of EC_50_/IC_50_).

**Table 4:** Inhibition (IC_50_), Antiviral (EC_50_) Activity, Ratio of EC_50_/IC_50_, and Equilibrium-Binding Constants *K*_i_ and *K*_inact_/*K*_i_ (if available) of SARS-CoV-2 M^PRO^ Covalent Inhibitors^a^

compound	IC_50_ (μM)	EC_50_ (μM)	EC_50_/IC_50_	*K*_i_ (μM) or/and *K*_inact_/*K*_i_ (M^–1^ s^–1^)	ref
aldehyde warhead					
GC-376*	0.19	0.92	4.84	0.094 μM	(30,31)
				2.84 × 10^4^ M^–1^ s^–1^	
UAWJ247*	0.045	2.06	45.78	0.035 μM	(30)
UAWJ9-36-1	0.051	2.56	50.2	8.5 × 10^4^ M^–1^ s^–1^	(40)
UAWJ9-36-3	0.054	0.37	6.85	9.3 × 10^4^ M^–1^ s^–1^	(40)
ketone warhead					
PF-00835231	0.0069			2.7 × 10^–4^ μM	(47)
YH-53		4.2		0.034 μM	(51)
α-ketoamide warhead					
*boceprevir*	1.59	1.90	1.19	1.18 μM	(32)
UAWJ246	0.045	4.61	102.44	0.036 μM	(30)
UAWJ248		11.1		0.013 μM	(30)
				9.1 × 10^4^ M^–1^ s^–1^	
Michael acceptor group as warhead					
N3		16.77		1.1 × 10^4^ M^–1^ s^–1^	(5,15,66)
**29**	2.86			38.36 μM	(72)
				18 × 10^4^ M^–1^ s^–1^	
**30**	0.42			4.5 μM	(73)
**31**	0.17			2.3 μM	(73)
myricetin	0.2–0.6	8.0	20	15.73 μM	(79)
				7.0 × 10^2^ M^–1^ s^–1^	
α-haloacetamide warhead					
**36**	0.4			16 μM	(73)
**38**	0.43	2.05	4.76	8.2 × 10^2^ M^–1^ s^–1^	(81)
**39**	0.08	2.15	26.87	7.1 × 10^3^ M^–1^ s^–1^	(81)
**40**	0.056			1.34 μM	(81,82)
				4.2 × 10^3^ M^–1^ s^–1^	
nitrile warhead					
nirmatrelvir	0.019	0.075	3.94	0.003 μM	(88,89)
ester warhead					
GRL-1720	0.32	15	46.87	2.15 μM	(52,100,101)
				1.9 × 10^4^ M^–1^ s^–1^	
GRL-0820	0.073	15	205.47	0.073 μM	(52,101)
**44**	0.0342			2.9 × 10^4^ M^–1^ s^–1^	(102)
selenium/sulfur as electrophilic warhead					
**61**	0.074			0.031 μM	(113)
				1.13 × 10^5^ M^–1^ s^–1^	
**62**	0.11			0.078 μM	(113)
				7.44 × 10^4^ M^–1^ s^–1^	

a Warhead masked as prodrug.

Considering α-ketoamide and carbonyl warheads, such as aldehydes and ketones, which can act as both reversible and irreversible derivatives, the enzymatic kinetic studies showed that compounds UAWJ246 (IC_50_ = 0.045 μM, EC_50_ = 4.61 μM, and ratio of EC_50_/IC_50_ = 102.44) and UAWJ247 (IC_50_ = 0.045 μM, EC_50_ = 2.06 μM, and ratio of EC_50_/IC_50_ = 45.78) reversibly bound the catalytic cysteine of SARS-CoV-2 M^PRO^ with inhibition constant *k*_i_ values of 0.036 ± 0.007 and 0.035 ± 0.008 μM, respectively. On the other hand, compounds UAWJ248 (EC_50_ = 11.1 μM) and GC-376 (IC_50_ = 0.19 μM, EC_50_ = 0.92 μM, and ratio of EC_50_/IC_50_ = 4.84), after an initial reversible binding complex with the enzyme, determined an irreversible inactivation with *K*_inact_/*K*_i_ values of 9.04 × 10^4^ and 2.84 × 10^4^ M^–1^ s^–1^, respectively, indicating a notable potency of UAWJ248.^30^

Derivatives with a Michael acceptor group as a warhead are generally irreversible SARS-CoV-2 M^PRO^ inhibitors. Kinetics data for compounds N3 (EC_50_ = 16.77 μM) and myricetin (IC_50_ = 0.2–0.6 μM, EC_50_ = 8.0 μM, and ratio of EC_50_/IC_50_ = 20) demonstrated an irreversible two-step mechanism for the inhibition of the SARS-CoV-2 M^PRO^. N3 exhibits a very potent inhibitory effect, making unachievable the *K*_i_ and *K*_inact_ measurement. Subsequently, a pseudo-second-order rate constant was determined, showing that N3 acts as a time-dependent irreversible inhibitor with *K*_inact_/*K*_i_ values of 1.1 × 10^4^ M^–1^ s^–1^.^5,15,66^ The covalent inhibitor myricetin shows a *K*_i_ value of 15.73 μM and a *K*_inact_ value of 0.011 s^–1^. The *K*_i_ value indicates that myricetin binds selectively to the binding pocket of the protease, providing the basis for irreversible covalent binding between myricetin and Cys^145^. The experimental *K*_inact_ value suggests that myricetin could react rapidly with the Cys^145^. Overall, considering the small size of myricetin, it is an efficient covalent binder of SARS-CoV-2 M^PRO^ with a *K*_inact_ /*K*_i_ of 701.88 M^–1^ s^–1^.^79^

α-Haloacetamide derivatives are also irreversible SARS-CoV-2 M^PRO^ inhibitors. In particular, enzymatic kinetic studies for compounds **38** (IC_50_ = 0.43 μM, EC_50_ = 2.05 μM, and ratio of EC_50_/IC_50_ = 2.76) and **39** (IC_50_ = 0.08 μM, EC_50_ = 2.15 μM, and ratio of EC_50_/IC_50_ = 26.87) proposed a two-step process for inhibition of SARS-CoV-2 M^PRO^: the first step is a reversible binding adduct, and the second step is an irreversible binding complex; these results were in agreement with the expected mechanism of action in which compounds **38** and **39** form a covalent bond with the catalytic Cys^145^.^81,102^ Compound **40** (IC_50_ = 0.056 μM) inhibited M^PRO^ in a time-dependent manner, indicating an irreversible mode of action with a *k*_i_ value of 1.34 μM and *K*_inact_/*k*_i_ value of 4167 M^–1^ s^–1^.^82^

Among the major covalent inhibitors of SARS-CoV-2 M^PRO^, nirmatrelvir, with a nitrile as the electrophilic warhead, showed remarkable biological data (IC_50_ = 0.019 μM, EC_50_ = 0.075 μM, and ratio of EC_50_/IC_50_ = 3.94) related to a notable *k*_i_ value of 0.003 μM.^88,89^

Compounds with an electrophilic ester warhead provide an irreversible inhibitory mechanism of SARS-CoV-2 M^PRO^. GRL-1720 and GRL-0820 (*k*_i_ values of 2.15 and 0.073 μM, respectively) exhibit potent M^PRO^ inhibitory activity, have second-order rate inactivation constants, and demonstrate a time-dependent inhibition. The mode of action involves acylation of Cys^145^ in the active site, supported by the catalytic dyad.^52,102,124^

Ebselen and ebsulfur derivatives exhibited a concentration- and time-dependent inhibition pattern against M^PRO^ with a biphasic character, suggesting that the rate of inactivation follows pseudo-first-order rate kinetics, implying irreversible covalent inhibition;^113^ among them, compounds **61** and **62** show *k*_i_ values of 0.031 and 0.078 μM and *K*_inact_/*K*_i_ values of 1.13 × 10^5^ and 7.44 × 10^4^ M^–1^ s^–1^, respectively.

In conclusion, in the present work, we provided a detailed analysis of the recent articles published in the literature in the last 3 years (2020–2022), focusing on the peptidomimetic and nonpeptidomimetic aspects of the covalent inhibitors against the SARS-CoV-2 main protease. As an update of previously published articles, we report only the in silico, in vitro, and in vivo biological data of the latest generation of inhibitors that have shown potent and high selectivity against the viral SARS-CoV-2 Main protease with a specific covalent inhibitory mechanism. Concentrating the attention on the electrophilic warheads, which are the pivotal moieties for the formation of the covalent adduct and the consequent reversible/irreversible mechanism of inhibition, we classified the studied molecules into eight different categories based on their reactive electrophilic groups and showed for each class the kinetic mechanism involved in the formation of the covalent adduct between the inhibitor and the catalytic Cys^145^ of the SARS-CoV-2 main protease. In particular, to better understand the 3D binding orientations of the ligands into the enzymatic binding pocket, we reported several X-ray crystallographic images and the corresponding list of PDB codes of the complexes. In addition, the analyses of the most recurrent pharmacophoric moieties and stereochemistry of the chiral carbons were reported to identify the most suitable molecular fragments (P1′, P1, P2, P3) for interaction with the crucial subregions (S1′, S1, S2, S3/S4) of the M^PRO^ catalytic cleft. In Tables 2 and 3, according to the Schechter–Berger notation, we analyzed and compared the pharmacophoric moieties that are crucial for the formation of the ligand/enzyme complex during the first reversible step of the inhibition mechanism. To find a correlation with the biological data IC_50_ and EC_50_ of the most active covalent inhibitors, the available values of the equilibrium binding constants *K*_i_ and *K*_inact_/*K*_i_ were listed in Table 4.

## Abbreviations Used

3CL^PRO^: 3C-like protease
ABPP: activity-based protein profiling
ADME: absorption, distribution, metabolism, and excretion
ADQN-FBDD: advanced deep Q-learning network and fragment-based drug design
AI: artificial intelligence
Cbz: benzyloxycarbonyl
CFA: α-chloro-fluoroacetamide
cLogP: calculated LogP
COVID-19: coronavirus disease-19
CPE: cytopathic effect
DFT: density functional theory
EC_50_: half-maximal effective concentration
EMA: European Medicines Agency
FCoV: feline coronavirus
FDA: Food and Drug Administration
GI: gastrointestinal
HMK: hydroxymethylketone
IC_50_: half-maximum inhibitory concentration
IL-6: interleukin 6
*K*_i_: inhibition constant
M^PRO^: main protease
MALDI-TOF: matrix-assisted laser desorption ionization-time of flight
MD: molecular dynamics
MERS-CoV: Middle East respiratory syndrome coronavirus
MM/PBSA: molecular mechanics Poisson–Boltzmann surface area
nM: nanomolar
Nsp: nonstructural proteins
PDB: Protein Data Bank
QM/MM: quantum mechanics/molecular mechanics
QSAR: quantitative structure–activity relationship
SAR: structure–activity relationship
SARS-CoV-1: severe acute respiratory syndrome coronavirus 1
SARS-CoV-2: severe acute respiratory syndrome coronavirus 2
SBDD: structure-based drug design
TNF-α: tumor necrosis factor α
